# The Multicriteria Decision Analysis for Extended Reality (MCDA-XR) Governance Framework for Health Care Adoption: Mixed Methods Development Study

**DOI:** 10.2196/89801

**Published:** 2026-07-31

**Authors:** José Ferrer Costa, Manuel Armayones Ruiz, Pierre Bourdin-Kreitz

**Affiliations:** 1 Behavioural Design Lab (BDLab), Doctoral Programme in Health and Psychology, Universitat Oberta de Catalunya, Pl Pau Casals 1, Badalona, Catalonia, 08911, Spain, 34 937407482; 2 Research and Innovation, Badalona Serveis Assistencials, Badalona, Catalonia, Spain; 3 Grup de Recerca Multidisciplinar en Salut i Societat (GREMSAS), IDIAP Jordi Gol, Institut Universitari d'Investigació en Atenció Primària Jordi Gol, Barcelona, Catalonia, Spain; 4 Computer Sciences Department (EIMT), Universitat Oberta de Catalunya, Barcelona, Catalonia, Spain; 5 XR-Lab of the Interdisciplinary Research and Innovation Hub, Open University of Catalonia, Barcelona, Catalonia, Spain

**Keywords:** extended reality, digital health governance, implementation science, multicriteria decision analysis, organizational readiness, strategic planning, decision support systems, health services administration

## Abstract

**Background:**

Health services increasingly face decisions about how to integrate immersive technologies into routine practice. International guidance highlights the need for structured governance in digital health, yet extended reality (XR) initiatives are often launched through isolated pilots without a clear assessment of organizational readiness or implementation risk. Although factors influencing XR adoption are well documented, health care organizations and system-level decision-makers still lack practical, governance-oriented tools to translate these determinants into structured strategic decisions made before implementation.

**Objective:**

This study aims to develop multicriteria decision analysis for extended reality (MCDA-XR), a strategic governance framework that translates behavioral, organizational, and technical implementation determinants into a structured decision-support process for health care organizations.

**Methods:**

The study followed a sequential mixed methods design covering the first 2 phases of a 3-stage framework development and validation project. Phase 1 (identification) defined strategic criteria by integrating theoretical perspectives on organizational complexity, behavior change, technology acceptance, and immersive safety, together with a targeted review of XR implementation evidence. Phase 2 (construction) refined the framework through participatory sessions. A multidisciplinary group of 33 stakeholders, including professionals and managers from hospital and primary care settings, and postgraduate students, evaluated the proposed criteria for strategic relevance and operational clarity. This process resulted in a refined 10-criterion structure and the establishment of a dual-score assessment logic. Phase 3 (validation), planned as a subsequent step, will examine how the framework performs when applied prospectively in clinical settings.

**Results:**

The development process yielded a framework comprising 10 operational criteria grouped into 3 conceptual domains (human, organizational, and technical). Stakeholder ratings indicated high strategic relevance across all criteria, with mean scores ranging from 4.03 (SD 0.95) for workflow integration to 4.61 (SD 0.56) for safety and comfort. The final instrument applies a dual-assessment approach in which each criterion is rated separately for strategic importance and organizational readiness. Mapping these dimensions enables organizations to identify priority gaps, particularly areas of high importance and low readiness, and to distinguish between manageable constraints and critical barriers requiring targeted preparatory action prior to implementation.

**Conclusions:**

MCDA-XR addresses a key governance gap in XR implementation by providing a structured way to align adoption decisions with institutional priorities and operational constraints. Rather than relying on descriptive feasibility assessments, the framework is intended to support explicit prioritization and action-oriented decision-making at the organizational level. MCDA-XR is positioned for Phase 3 evaluation, which will examine the practical utility, interpretability, and implementation relevance of the framework when applied prospectively in real-world clinical deployments.

## Introduction

### Background

Health systems are increasingly aware that emerging technologies cannot be integrated through intuition or isolated initiatives. International guidance has moved in the same direction. World Health Organization (WHO) 2019 Guidelines on Digital Interventions [1] stress that digital tools should only be introduced following structured assessment and a clear understanding of the health needs they aim to address. The Global Strategy on Digital Health 2020‐2025 [2] reinforces this idea by calling for national planning processes that align digital priorities with institutional capacity and continuous evaluation. Recent European policy work on virtual worlds and health further emphasizes that adoption is shaped by organizational readiness, governance arrangements, and structural barriers that extend beyond technical performance [3]. Together, these documents point to a straightforward conclusion. Sustainable digital health requires methods that help organizations define priorities, assess feasibility, and prepare for implementation before technologies are deployed.

Immersive technologies complicate this picture. Extended reality (XR), which includes virtual reality (VR), augmented reality (AR), and mixed reality (MR), introduces sensory and spatial characteristics that differ from conventional digital tools, including immersive perceptual environments and embodied interaction mechanisms [4,5]. Evidence from experimental and clinical VR research shows that mechanisms such as presence and embodiment can shape attention, emotional responses, and body perception in ways linked to therapeutic effect [6,7]. Variables including presence, body ownership, and immersion are therefore not peripheral features of the user experience; they are part of the mechanism through which many XR interventions act. Immersive systems also alter cognitive and sensory demands. Spatial processing requirements and interface complexity can influence task performance and learning, as described in the cognitive affective model of immersive learning [8], while sensory conflict between visual and vestibular input can lead to cybersickness and discomfort, affecting tolerability [9]. Because these perceptual and cognitive dimensions fall outside the assumptions underpinning conventional digital health evaluation models, XR adoption requires a structured multicriteria approach, yet no clear operational governance tool currently supports this kind of preimplementation decision-making in health care.

Although research across clinical and educational fields reports benefits in pain and anxiety management, rehabilitation, and mental health care [4,10,11], evidence alone has not guaranteed systematic integration into health care services. Reviews show that routine uptake remains uncommon despite promising results [12]. These findings indicate that immersive technologies hold meaningful clinical and pedagogical value, yet translation into routine practice is constrained by organizational, professional, and governance factors that are not addressed by efficacy evidence alone. This perspective aligns with recent digital health policy work highlighting the need for governance approaches, understood here as the institutional processes used to evaluate, prioritize, and guide adoption, that help organizations relate implementation evidence to strategic prioritization and readiness decisions, particularly when choices must be made about which innovations to support and when [2,3].

This study addresses the lack of an XR-specific preimplementation framework that translates known adoption determinants into operational criteria for governance, prioritization, and readiness assessment. To address this gap, this article introduces multicriteria decision analysis for extended reality (MCDA-XR), a governance-oriented multicriteria decision-support framework designed to integrate dispersed implementation evidence and support health care organizations evaluating, prioritizing, and planning XR initiatives within their broader digital health strategy. Although MCDA-XR draws on the logic of multicriteria decision analysis, it is not yet presented here as a fully formal multicriteria decision analysis (MCDA) model with established weighting, aggregation methods, or decision rules. The dual-score structure proposed in this article provides the initial operational basis of the framework, while formal refinement of its weighting and aggregation logic, together with prospective validation, is being undertaken in Phase 3.

### Evaluation of XR Implementation: Evidence, Attempts, and Gaps

#### Real-World Implementation Evidence: A Fragmented Landscape

When immersive systems leave controlled settings and enter clinical practice, the frictions become visible. Spatial layout, device handling, hygiene routines, and tracking stability repeatedly appear as determinants that shape feasibility during deployment [12-14]. Recent reports from hospital-based XR programs add further texture, noting the need for clear protocols on headset management, staffing, patient selection, and environmental safety [15]. Rehabilitation, primary care, and mental health studies echo this pattern. Many show therapeutic potential, yet routine adoption remains uncommon [12,14,16]. Organizational constraints, workflow misalignment, limited training, and inconsistent technical support continue to slow implementation. Work in chronic pain paints a similar picture [17].

Across settings, the pattern is similar. Many implementation problems only become visible once XR is used in routine care, which leaves teams reacting to issues that could have been anticipated earlier. This gap makes it harder to evaluate feasibility, coordinate staff and equipment, or clarify how XR fits within existing care pathways. A more structured way of assessing readiness may help shift this work to the planning stage, when organizations still have room to adjust. To substantiate this landscape, Multimedia Appendix 1 provides a detailed comparative overview of the methodological, behavioral, and organizational determinants reported across the primary studies reviewed.

#### Determinant Mapping Attempts: What Reviews Have Identified

Attempts to explain how XR becomes embedded in clinical and educational settings have expanded quickly. Kouijzer et al [12] offer one of the most extensive syntheses and show how similar determinants recur across contexts, including clinician capability, workflow fit, organizational backing, and patient acceptability. They also note substantial variation in methodological quality, which complicates comparison.

In chronic pain, Elser et al [17] used the theoretical domains framework to highlight the weight of beliefs about consequences, emotional responses, and self-efficacy, yet their findings also imply that behavioral determinants alone cannot explain organizational pressures or service-level constraints. Work in education reaches similar conclusions. Lie et al [14] describe how training structures, institutional support, and professional identity shape the use of immersive tools.

Taken together, these scoping reviews show that the most relevant determinants have been identified, although they remain scattered across studies and have not yet been arranged into a structure that helps with planning or priority-setting.

#### Digital Health Implementation Frameworks: Useful but Partial

In digital health, evaluation typically relies on established implementation science models. These frameworks illustrate how individuals, organizations, and technologies interact, building on behavioral determinants described by Michie et al [18], the organizational complexity mapped by Greenhalgh and Abimbola [19], and the processes synthesized in the consolidated framework for implementation research (CFIR) [20]. Broader overviews classify these models into determinant frameworks, process models, and theories, each covering a different part of the implementation landscape [21]. More recent work stresses the importance of understanding mechanisms of action, noting that evaluation depends on clarifying how behavioral, organizational, and contextual influences shape implementation outcomes [22].

These perspectives are valuable, although none was designed to address the experiential or embodied features of immersive systems. While specific guidelines such as the Reporting of Applications, Technologies, and Experiments in Extended Reality (RATE-XR) guideline [23] contribute essential descriptors for safety and reporting quality, they focus on technical transparency rather than organizational strategy. To place XR within this broader space, Table 1 summarizes the implementation and behavioral frameworks most relevant to the field, detailing their core purpose, main strengths, limitations in immersive contexts, and specific conceptual contributions to the MCDA-XR framework.

**Table 1.** Analytical comparison of the implementation and behavioral frameworks used to derive the MCDA-XR^a^ framework.

Framework	Core purpose	Strengths for XR^b^ adoption evaluation	Limitations in real-world XR contexts	Relevance to MCDA-XR
CFIR^c^ [20]	Diagnostic meta-framework for implementation contexts.	Provides detailed insight into organizational climate, leadership, workflow, and resource constraints; widely used in health services research.	High conceptual complexity, difficult to translate into concise scoring, no XR-specific constructs.	Informs assessment of organizational readiness and implementation context.
NASSS^d^ [19,24]	Explains complexity and adoption patterns across 7 domains.	Strong systemic and organizational perspective, well suited to digital health evaluation.	Broad and descriptive, limited behavioral granularity, requires adaptation for immersive technologies.	Serves as the organizational and systemic backbone of MCDA-XR.
TAM^e^ [25]	Predicts technology acceptance through perceived usefulness and ease of use.	Simple, validated, and effective for capturing user perceptions of XR.	Narrow cognitive focus, does not address organizational, experiential, or behavioral determinants.	Its core acceptance-related constructs inform user-perception and value-related criteria.
UTAUT^f^ and UTAUT2 [26,27]	Predicts behavioral intention based on performance expectancy, effort expectancy, and social influence.	Captures social and cognitive determinants of acceptance with strong explanatory capacity.	Relies on multi-item questionnaires with overlapping constructs, limiting feasibility in clinical contexts.	Relevant acceptance-related determinants are represented indirectly through TAM and COM-B^g^; the full UTAUT models were not used for scoring.
TDF^h^ [28]	Synthesizes behavioral determinants across 14 domains and more than 80 constructs.	Enables detailed behavioral diagnosis and identification of individual-level barriers.	Highly granular, not designed for scoring, and not XR-specific.	Provides detailed behavioral background, but was not used directly for scoring.
COM-B [18]	Models behavior through capability, opportunity, and motivation.	Parsimonious, explanatory, and easy to operationalize; integrates individual and contextual factors.	Less detailed than TDF and requires organizational complement.	Serves as the primary behavioral foundation for operationalization in MCDA-XR.
BCW^i^ [18]	Framework for designing behavior-change interventions based on COM-B.	Useful for structuring training and support strategies related to XR use.	Intervention design tool rather than an evaluation framework; unsuitable for concise scoring.	Conceptually relevant for implementation support strategies, but not directly operationalized in the framework.
LHS^j^ [29]	Promotes iterative, data-driven learning cycles in healthcare.	Supports continuous evaluation, feedback, and sustainability.	High-level framework lacking explicit evaluative constructs.	Justifies iterative application of MCDA-XR across project phases.
RATE-XR^k^ [23]	Reporting guideline for early-phase XR clinical research focused on safety, technical fidelity, and methodological transparency.	Provides XR-specific descriptors for safety, cybersickness, tolerability, sensory exposure, and technical detail.	Not an implementation framework and does not address organizational or behavioral determinants.	Ensures methodological and safety alignment within XR-specific criteria of MCDA-XR.

a MCDA-XR: multicriteria decision analysis for extended reality.

b XR: extended reality.

c CFIR: consolidated framework for implementation research.

d NASSS: non-adoption, abandonment, scale-up, spread, sustainability.

e TAM: technology acceptance model.

f UTAUT: unified theory of acceptance and use of technology.

g COM-B: capability, opportunity, motivation-behaviour.

h TDF: theoretical domains framework.

i BCW: behaviour change wheel.

j LHS: Learning Health Systems.

k RATE-XR: Reporting of Applications, Technologies, and Experiments in Extended Reality.

#### Precedents for Integrative and Multicriteria Evaluation

A smaller body of work has begun to move beyond single-theory evaluations by explicitly linking behavioral, organizational, and technological perspectives. Within the immersive field, Chung et al [30] combined the theoretical domains framework; capability, opportunity, motivation-behaviour (COM-B); the behaviour change wheel, and implementation strategy mapping to inform the implementation of VR in psychiatry. This work represents one of the first structured applications of behavioral implementation frameworks to XR. Its scope, however, remains largely confined to mental health settings and centers mainly on clinician behavior, with limited attention to organizational governance, resource allocation, or system-level decision-making.

Beyond XR, recent digital health research offers relevant methodological precedents for the integrative structure proposed here. Pereira Guerreiro et al [31] showed that non-adoption, abandonment, scale-up, spread, sustainability (NASSS) and COM-B can be applied in parallel to assess complex medical devices, suggesting that behavioral and organizational lenses can be meaningfully combined within a single evaluation process. In a complementary direction, Deason et al [32] demonstrated the feasibility of using multicriteria decision analysis to support virtual care selection, shifting the focus from narrative appraisal to structured and transparent scoring. Taken together, these studies point to a clear opportunity. While integrative and multicriteria approaches are already being used in broader digital health, they have not yet been translated into a unified governance framework tailored to the specific characteristics and risks of XR.

#### Why Existing Approaches Remain Insufficient for XR

Despite their contributions, existing frameworks fall short when applied to immersive technologies. Behavioral models such as COM-B and the theoretical domains framework [18,28] clarify capability, opportunity, and motivation, but do not engage with the sensory, ergonomic, or embodied features that shape XR use [4-9,23]. Organizational frameworks such as CFIR and NASSS describe complexity and system dynamics [19,20], yet offer little guidance on perceptual mechanisms or user experience. Usability approaches assess interaction quality [13], but do not address institutional support or sustainability [12,14-17]. RATE-XR provides safety and reporting descriptors [23] rather than readiness or strategic planning criteria.

Each model captures a relevant part of the picture. What is missing is a way to integrate behavioral, organizational, experiential, and contextual determinants into a structure that supports comparison, prioritization, and planning. In practice, decisions about XR adoption require weighing clinical value, safety, workflow compatibility, organizational capacity, regulatory constraints, and cost. These dimensions rarely move in the same direction, and no single variable captures the trade-offs that determine whether an initiative is viable within a health care organization.

#### The Governance Gap: Need for an Operational Strategic Framework

Across the literature, there is agreement that the determinants influencing XR adoption are broadly known, yet they remain dispersed across frameworks with different aims [12,14,17,30]. No current model captures behavioral, organizational, and experiential determinants within a single governance structure that can be applied explicitly to strategic portfolio management in digital health. Existing tools describe barriers, but they do not translate them into assessable criteria or link them to resource allocation, which limits their usefulness for planning.

A multicriteria decision approach offers a way to address this gap. By allowing organizations to weigh criteria according to local priorities, such approaches support comparison between initiatives that differ in purpose, maturity, or resource demand. An organization may prioritize safety during early piloting, focus on workflow integration during scale-up, or emphasize evidence requirements when projects compete for investment. Rather than relying on intuition, multicriteria methods make these trade-offs explicit and transparent.

To address this governance gap, this study describes the development and formative refinement of MCDA-XR, a multicriteria governance framework designed to support feasibility assessment, readiness evaluation, and strategic prioritization of XR initiatives within healthcare organizations.

## Methods

### Study Design

This study follows a sequential mixed methods design and forms part of a broader doctoral project structured around 3 developmental phases: identification, construction, and validation of a strategic governance framework for XR adoption in health care. This paper reports the methods and findings from Phases 1 and 2, which define the conceptual structure and operational logic of the MCDA-XR framework. Phase 3, focused on longitudinal validation in real-world settings, falls outside the scope of this paper.

**Figure 1.**
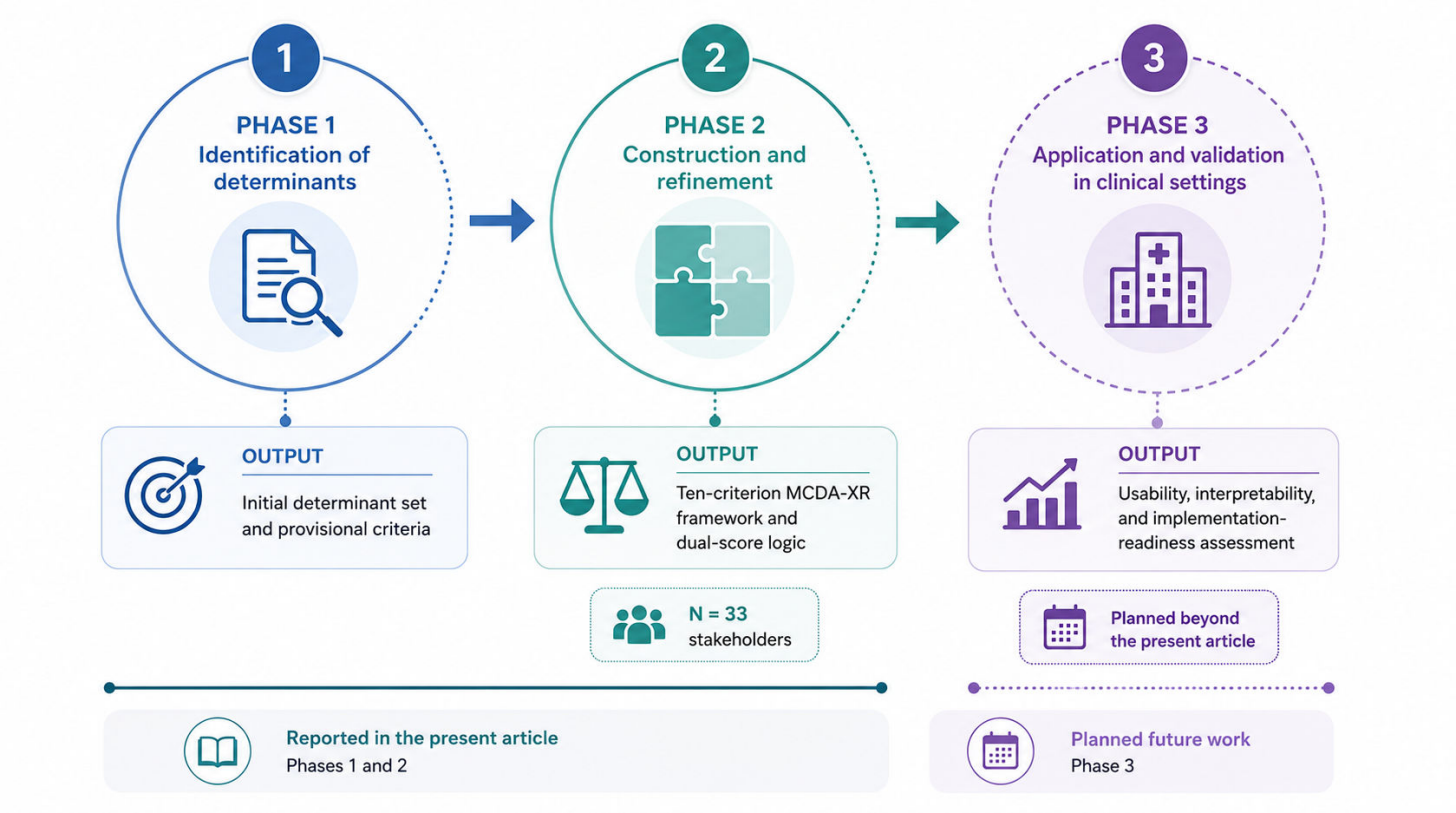
Sequential mixed methods design for the development and validation of the multicriteria decision analysis for extended reality (MCDA-XR) framework. MCDA-XR: multicriteria decision analysis for extended reality framework.

Across all phases, the study adopts a pragmatic implementation perspective. Established theories of digital health governance and behavior change were combined with empirical evidence and stakeholder input to ensure that the framework remains theoretically grounded while reflecting the operational realities of health care organizations. Figure 1 summarizes this sequence, showing how determinant identification informed the construction and refinement of MCDA-XR, and how the resulting framework will be applied prospectively in Phase 3. Detailed procedures for each phase are described below.

### Setting and Context

The study was conducted within the Doctoral Program in Health and Psychology at the Open University of Catalonia (UOC), which provided methodological oversight. Framework development was anchored at Badalona Serveis Assistencials (BSA), an integrated health care organization functioning as a living lab for applied innovation. Evidence reviewed in Phase 1 and stakeholder contributions in Phase 2 extended beyond this setting, drawing on experience from hospital and primary care services as well as academic research environments. For the formative activities in Phase 2, participants were drawn from the BSA living lab environment and collaborating academic and clinical settings in order to capture perspectives from both routine care and training contexts relevant to early XR appraisal. This approach allowed the framework to be informed by diverse XR implementation contexts while remaining closely aligned with the governance and organizational constraints of routine clinical practice.

### Phase 1: Identification of Strategic Determinants

#### Overview

Phase 1 aimed to identify the behavioral, organizational, and technical variables that define the strategic feasibility of XR initiatives. The work unfolded in 3 sequential steps that moved from theoretical framework selection to empirical evidence validation and final criterion expansion.

#### Step 1: Strategic Framework Selection and Synthesis

In the first step, theoretical models suitable for strategic decision-making were identified through an analytical literature review (Table 1). Four frameworks were selected for their complementary coverage of system-level, behavioral, perceptual, and XR-specific considerations. Their joint use followed established precedents for integrating behavioral, organizational, and decision-analytic perspectives in digital health (Table 2). Core domains from the selected frameworks were intersected to derive an initial set of provisional evaluative dimensions.

**Table 2.** Methodological precedents informing the development of the MCDA-XR^a^ framework.

Study	Scope	Methods	Strengths	Limitations	Relevance to MCDA-XR
Chung et al (2022) [30]	XR^b^ implementation in mental health.	Integration of TDF^c^, COM-B^d^, BCW^e^, and ERIC^f^.	Provides the first structured behavioral toolkit for XR.	Limited to psychiatric settings; minimal organizational or systemic modeling.	Offers behavioral precedent for multiframework integration.
Pereira Guerreiro et al (2025) [31]	Implementation of a non-XR medical device.	Parallel use of NASSS^g^ and COM-B.	Shows that behavioral and organizational perspectives are complementary.	Conceptual integration only; no unified scoring or weighting.	Supports the rationale for multilevel integration in MCDA-XR.
Deason et al (2025) [32]	Technology selection in virtual care.	Application of MCDA^h^ to digital health.	Demonstrates the feasibility of structured multicriteria decision-making.	Not XR-specific; lacks behavioral and organizational determinants.	Reinforces MCDA as an appropriate analytical approach for XR adoption.

a MCDA-XR: multicriteria decision analysis for extended reality.

b XR: extended reality.

c TDF: theoretical domains framework.

d COM-B: capability, opportunity, motivation-behaviour.

e BCW: behaviour change wheel.

f ERIC: Expert Recommendations for Implementing Change.

g NASSS: non-adoption, abandonment, scale-up, spread, sustainability.

h MCDA: multicriteria decision analysis for extended reality.

#### Step 2: Evidence Validation via Literature Review

To test the deductive structure derived in Step 1, a targeted review of XR implementation studies was conducted. Multimedia Appendix 1 compiles the literature informing framework development, including both the foundational and methodological precedents used to support the conceptual structure of MCDA-XR [12,13,15-17,30-32] and the targeted empirical corpus of XR implementation studies used for determinant extraction in Step 2 [33-45]. Determinants were defined as explicit barriers, facilitators, or requirements related to XR implementation and were extracted exclusively from the “Results” and “Discussion sections to preserve analytical fidelity.

This process used a large language model (LLM)–assisted workflow using ChatGPT (OpenAI GPT-family models) within a constrained extraction protocol designed to identify source-grounded verbatim segments. All extracted items generated with the final prompt were subsequently checked manually against the source documents to confirm attribution accuracy and verbatim fidelity. The resulting extraction matrix, together with the full extraction protocol and prompt, is provided in Multimedia Appendix 2.

#### Step 3: Strategic Gap Analysis and Expansion

Following extraction and verification, a thematic synthesis was performed within each evaluative dimension using a second constrained LLM-assisted workflow. Extracted determinants were examined to identify recurring patterns and higher-order themes, with conceptually overlapping determinants grouped through rule-guided semantic clustering. All outputs were manually reviewed to confirm attribution accuracy, conceptual consistency, and alignment with the original determinant set and study context. Themes were classified according to their frequency across studies and their potential strategic impact, with particular attention to safety, ethical, and regulatory issues.

This synthesis informed the refinement and expansion of the analytical structure. Multimedia Appendix 3 documents the thematic synthesis procedure, including the analytical prompt, clustering logic, and resulting evidence matrix by criterion. Multimedia Appendix 4 then presents the final theoretical-empirical alignment used to define each criterion operationally and to support the participatory appraisal and refinement activities conducted in Phase 2.

### Phase 2: Construction and Refinement

#### Overview

Phase 2 examined how the refined evaluative dimensions functioned when translated into a practical decision-support instrument. The focus was on interpretability, perceived strategic relevance, and operational usability across professional profiles and care settings.

Participants were recruited purposively to capture a range of perspectives relevant to early appraisal of XR implementation. Eligibility for this phase required attendance at one of the structured group sessions and completion of the prototype MCDA-XR exercise. Four structured group sessions were conducted within the BSA living lab environment and collaborating institutions. Each session followed a common format. Before completing the appraisal exercise, participants received a brief introduction to XR use in health care and a live demonstration of immersive applications used in clinical and educational projects. They were also introduced to the 10 criteria through their guiding questions and illustrative implementation examples, such as cybersickness, workflow compatibility, and data privacy, derived from the operational definitions developed in Phase 1 and documented in Multimedia Appendix 4. This introductory segment was intended to provide a common experiential reference point for the subsequent evaluation part, but it did not constitute formal XR training or extended hands-on use.

Participants then completed a prototype version of the MCDA-XR instrument, rating the importance of each criterion using a 5-point scale and providing brief qualitative comments. These comments were used to identify ambiguities, assess interpretability, and indicate whether any relevant implementation criterion appeared to be missing from the proposed structure.

A total of 33 participants contributed to the Phase 2 construction and rating process. The sample comprised practicing health professionals (eg, medicine, nursing, physiotherapy, and psychology), operational and managerial staff (eg, service leadership and administration), alongside postgraduate psychology students. This composition allowed the criteria to be examined from both an applied organizational perspective and a behavioral science perspective during this formative stage. Detailed participant characteristics are reported in the “Results” section.

This phase was intentionally formative. Its objective was to refine clarity and usability rather than to establish psychometric validity. The combined dataset of quantitative ratings and qualitative feedback informed iterative adjustments to wording and structure, preparing the instrument for empirical testing in Phase 3.

#### Data Collection and Analysis

Qualitative material consisted of narrative comments generated during the construction activities and additional documentary sources from exploratory stakeholder work. These data reflected how participants interpreted each criterion and related it to their organizational context. Analysis followed Braun and Clarke’s [46] reflexive thematic approach, combining inductive identification of emergent insights with deductive mapping informed by NASSS and COM-B. The aim in this phase was to refine conceptual wording and identify structural ambiguities requiring clarification.

Quantitative data consisted solely of importance ratings for the 10 criteria. Descriptive statistics, including means and SDs, were calculated for the full sample and for subgroups comparing practicing professionals versus postgraduate psychology students, and participants with no prior XR experience versus those with any prior XR experience. These summaries were used to describe rating patterns across participant profiles and to inform iterative refinement of the instrument, but were not used for inferential analysis. Hypothesis testing and validation of scoring behavior are planned for Phase 3, where the MCDA-XR protocol will be applied in real implementation scenarios.

#### Ethical Considerations

The study was approved by the UOC Research Ethics Committee (CE25-TE78). Participation is voluntary and all contributors provide informed consent. Information is pseudonymized before analysis and no patient-level data are included. Experiential insights from XR projects are handled in line with the approved protocol and comply with General Data Protection Regulation (GDPR) and Organic Law on Protection of Personal Data and Guarantee of Digital Rights (LOPDGDD) requirements.

Reflexive notes were maintained throughout to monitor the potential influence of the researcher’s embedded role within the organization, consistent with guidance for reflexivity in applied health research.

## Results

### Phase 1: Evolution of the MCDA-XR Structure

#### Overview

Phase 1 resulted in the transformation of an initial deductive structure into a governance framework composed of 10 operational criteria. This evolution reflects how theoretical assumptions were reshaped when tested against empirical evidence from XR implementation studies.

The evolution of the framework clarified how different theoretical perspectives held up under empirical testing. NASSS primarily structured system- and organization-level considerations, while COM-B and technology acceptance model (TAM) informed criteria related to adoption, usability, and behavioral feasibility. RATE-XR contributed safeguards linked to safety, transparency, and technical integrity. Empirical mapping confirmed the relevance of these contributions, but also showed that governance, evidence legitimacy, and regulatory alignment could not remain implicit within organizational domains and required explicit representation as independent criteria.

#### From 7 Provisional Criteria to a 10-Criterion Structure

The initial synthesis produced 7 provisional criteria addressing relevance, safety and comfort, usability, workflow integration, resources and cost, training requirements, and patient acceptability. Together, these criteria captured core condition, technology, organizational, and adopter-related considerations relevant to XR adoption.

When empirical determinants from the literature were mapped onto this structure, clear limitations became apparent. A substantial proportion of extracted determinants could not be meaningfully accommodated within the original criteria without stretching their scope. In particular, issues related to evidence quality, institutional commitment, and regulatory or ethical compliance repeatedly emerged as distinct considerations rather than secondary aspects of organizational readiness.

To resolve these structural mismatches, the framework was expanded to include 3 additional criteria: evidence and credibility, institutional support, and legal and ethical alignment. Their inclusion marked a shift from an implicitly organizational interpretation of system-level factors to an explicit recognition of governance, legitimacy, and accountability as independent strategic determinants. The resulting 10-criterion structure provided a more faithful representation of the empirical landscape observed across XR implementation studies. The final allocation of determinants to criteria is documented in the extraction matrix (Multimedia Appendix 2).

#### Thematic Refinement and Operational Definition

Following the establishment of the 10-criterion structure, determinants within each domain were examined to identify recurring patterns and higher-order themes. Themes were classified as major when supported by 3 or more independent studies and as critical when directly related to safety, ethical, or regulatory requirements, regardless of frequency.

This synthesis informed the refinement of the scope and wording of each criterion, ensuring conceptual clarity and practical interpretability. Multimedia Appendix 3 presents the thematic structure by criterion, while Multimedia Appendix 4 provides the final operational definitions and example indicators that guided the framework construction activities in Phase 2.

#### The MCDA-XR Framework and Governance Matrix

Phase 1 concluded with the definition of the MCDA-XR framework, comprising 10 operational criteria intended to guide strategic decision-making on XR adoption in health care.

The resulting structure is formalized as a governance matrix that brings behavioral, organizational, and technical considerations into a single analytical space. Rather than treating these dimensions as parallel or sequential factors, the matrix reflects how they interact when XR initiatives are assessed, prioritized, and implemented in real-world settings.

**Figure 2.**
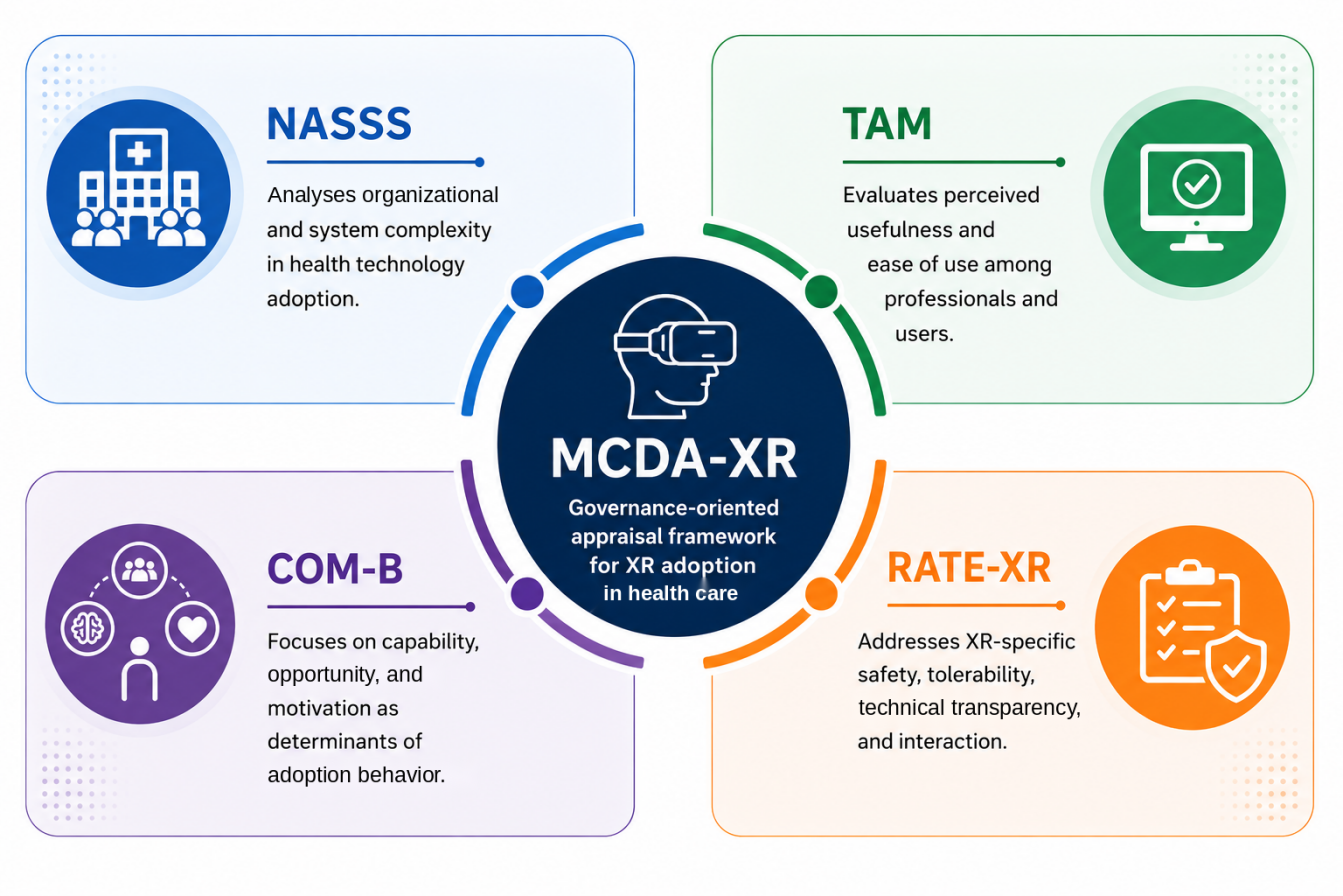
Conceptual integration of theoretical perspectives within the multicriteria decision analysis for extended reality (MCDA-XR) governance matrix. COM-B: capability, opportunity, motivation-behaviour; NASSS: non-adoption, abandonment, scale-up, spread, sustainability; RATE-XR: Reporting of Applications, Technologies, and Experiments in Extended Reality; TAM: technology acceptance model; XR: extended reality.

This synthesis does not seek to replace existing models. Its purpose is to translate their combined insights into a pragmatic diagnostic tool that makes misalignments visible, particularly those between technological readiness, workforce capability, and organizational or policy constraints. Figure 2 illustrates the structure and logic of the MCDA-XR governance matrix.

#### The 10 Strategic Criteria

The 10 MCDA-XR criteria fall into 3 broad layers of implementation. One layer concerns people, their capability to use XR, and the conditions that make its use realistic in daily care. Another relates to organizational structures, workflows, and resource constraints. A third concerns the technology, its safety, usability, and alignment with sector standards such as RATE-XR.

These layers are not treated as isolated components. They help identify where tensions or enablers usually appear as teams move from conceptual ideas to operational deployment.

Table 3 summarizes the 10 criteria and shows how each one aligns with the NASSS, COM-B, TAM, and RATE-XR constructs. The table also provides the guiding questions that form the basis of the decision-support instrument. Within MCDA-XR, these concepts are defined operationally through the criterion wording and guiding questions. For example, safety refers to tolerability and comfort during XR use, usability to the manageability and reliability of the system in practice, and patient experience to trust, enjoyment, and acceptance as part of care.

**Table 3.** Operational derivation and theoretical mapping of the 10 MCDA-XR^a^ criteria.

NASSS^b^ area (structural anchor) and MCDA-XR 10 criteria	Cross-framework basis (COM-B^c^, TAM^d^, and RATE-XR^e^)	MCDA-XR guiding question (rated 1‐5)
Condition		
Relevance	Capability (COM-B), perceived usefulness (TAM), clinical indication descriptor (RATE-XR)	Does the XR^f^ intervention address real clinical needs and patient goals?
Evidence and credibility	Reflective motivation (COM-B), perceived usefulness (TAM), evidence transparency (RATE-XR)	Is its use supported by scientific evidence and recognized clinical legitimacy?
Technology		
Safety and comfort	Physical capability and automatic motivation (COM-B), sensory and ergonomic descriptors (RATE-XR)	Is the XR experience safe, tolerable, and comfortable for patients?
Usability	Physical capability (COM-B), perceived ease of use (TAM), usability and interaction fidelity (RATE-XR)	Is the system intuitive, reliable, and manageable for clinicians and patients?
Value proposition and workflow		
Integration in workflow	Physical and social opportunity (COM-B), perceived usefulness (TAM), workflow compatibility (RATE-XR)	Does XR fit realistically within available time, routines, and workspace?
Resources and cost	Opportunity (COM-B), perceived usefulness (TAM), operational requirements (RATE-XR)	Are the required resources, staffing demands, and maintenance needs feasible?
Adopters (patients and professionals)		
Training requirement	Capability (COM-B), perceived ease of use (TAM), supervision and training descriptors (RATE-XR)	Can required training be achieved with current time, skills, and support?
Patient acceptability	Motivation (COM-B), perceived usefulness (TAM), user experience descriptors (RATE-XR)	Do patients trust, enjoy, and accept XR as part of their care?
Organization		
Institutional support	Social opportunity (COM-B), motivational alignment (COM-B), organizational readiness (RATE-XR)	Does the institution provide the support needed for meaningful and sustained use?
Wider system		
Legal and ethical alignment	Opportunity (system constraints; COM-B), compliance descriptors (RATE-XR)	Does the intervention meet ethical, regulatory, and data-protection requirements?

a MCDA-XR: multicriteria decision analysis for extended reality.

b NASSS: non-adoption, abandonment, scale-up, spread, sustainability.

c COM-B: capability, opportunity, motivation-behaviour.

d TAM: technology acceptance model.

e RATE-XR: Reporting of Applications, Technologies, and Experiments in Extended Reality.

f XR: extended reality.

To support traceability, the empirical themes that informed these operational definitions are presented in Multimedia Appendix 3, and the detailed conceptual-empirical mappings grounding each criterion in the NASSS, COM-B, TAM, and RATE-XR constructs are provided in Multimedia Appendix 4. Together, these materials document the analytic steps that link the thematic synthesis to the final structure summarized in Table 3.

#### Stakeholder Importance Ratings in Phase 2

A total of 33 participants contributed importance-weighting data. The sample was predominantly female (28, 84.8%). Ages ranged from 18 to 69 years and were distributed as follows: 18‐29 (12/33, 36.4%) years, 30‐39 (6/33, 18.2%) years, 40‐49 (6/33, 18.2%) years, 50‐59 (5/33, 15.2%) years, and 60‐69 (4/33, 12.1%) years. Reflecting the study’s aim to capture a broad cross-section of the health care ecosystem, the sample included 21 (63.6%) practicing health professionals from primary care, intermediate care, mental health, and neurorehabilitation services, alongside 12 (36.4%) postgraduate psychologists enrolled in a Master’s program in Psychogerontology. Detailed characteristics, including specific institutional origins (hospital, primary care, and university settings) and professional roles for all participants, are provided in Multimedia Appendix 5. Prior exposure to XR was limited: 16/33 (48.5%) participants reported no previous experience, 13/33 (39.4%) recreational-only use, and 4/33 (12.1%) had been involved in a clinical pilot.

**Figure 3.**
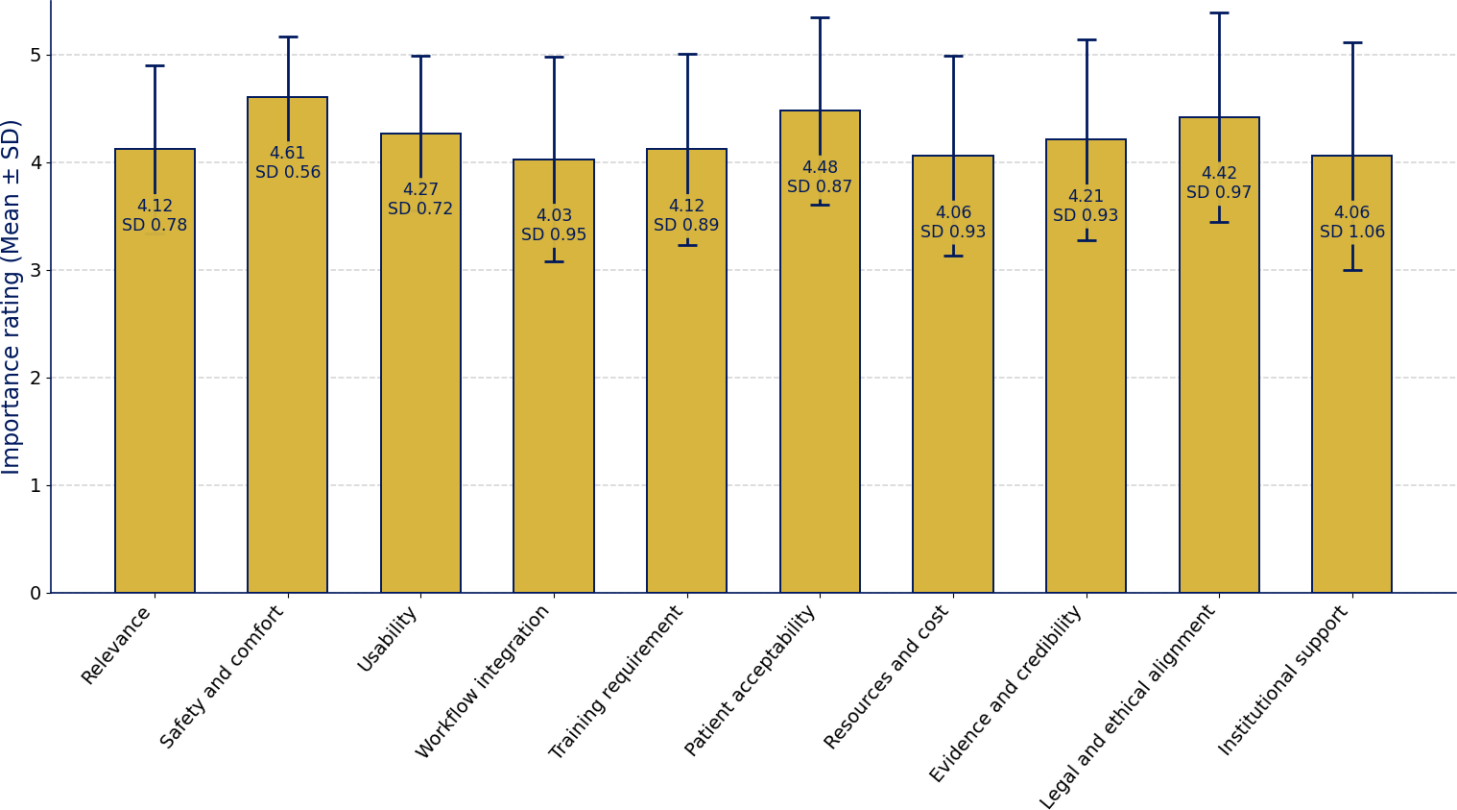
Operational derivation and theoretical mapping of the 10 multicriteria decision analysis for extended reality (MCDA-XR) criteria.

Participants rated all 10 MCDA-XR criteria on a 1‐5 scale, where 1 reflected the lowest strategic importance and 5 the highest. In the pooled Phase 2 sample, mean ratings ranged from 4.03 (SD 0.95) for workflow integration to 4.61 (SD 0.56) for safety and comfort (Figure 3). Safety and comfort received the highest mean score (4.61) and showed the lowest variability (SD 0.56), indicating the highest level of agreement among participants. Greater variability was observed for institutional support (SD 1.06) and legal and ethical alignment (SD 0.97), reflecting a wider spread of ratings across the sample.

Descriptive subgroup analyses are presented in Table 4. Overall, rating patterns were broadly similar across participant profiles, with all criteria remaining in the upper range of the scale. Some differences in relative emphasis were observed. Compared with postgraduate students, practicing professionals gave higher ratings to workflow integration, training requirements, and institutional support, whereas students rated relevance and usability slightly higher. When ratings were examined according to prior XR exposure, participants without prior XR experience gave higher ratings to evidence and credibility, legal and ethical alignment, and institutional support than those with any prior XR experience. Given the small subgroup sizes, these comparisons are descriptive only.

**Table 4.** Descriptive subgroup analysis of MCDA-XR^a^ importance ratings by participant profile and prior XR^b^ experience.

Criterion	Professionals (no XR; n=10), mean (SD)	Professionals (any XR; n=11), mean (SD)	Students (no XR; n=6), mean (SD)	Students (any XR; n=6), mean (SD)
Relevance	4.20 (0.63)	3.82 (0.87)	4.00 (0.89)	4.67 (0.52)
Safety and comfort	4.60 (0.52)	4.55 (0.52)	4.67 (0.82)	4.67 (0.52)
Usability	4.20 (0.63)	4.18 (0.87)	4.33 (0.82)	4.50 (0.55)
Workflow integration	4.20 (0.92)	4.18 (1.25)	3.67 (0.82)	3.83 (0.41)
Training requirement	4.40 (0.97)	4.09 (0.94)	4.17 (0.98)	3.67 (0.52)
Patient acceptability	4.50 (0.71)	4.36 (1.21)	4.50 (0.84)	4.67 (0.52)
Resources and cost	4.00 (1.25)	4.09 (0.94)	4.00 (0.63)	4.17 (0.75)
Evidence and credibility	4.60 (0.52)	3.82 (1.25)	4.17 (0.75)	4.33 (0.82)
Legal and ethical alignment	4.70 (0.48)	4.18 (1.25)	4.50 (0.84)	4.33 (1.21)
Institutional support	4.40 (0.70)	3.91 (1.51)	4.17 (0.75)	3.67 (0.82)

a MCDA-XR: multicriteria decision analysis for extended reality.

b XR: extended reality.

Open-text comments were brief and generally positive, noting perceived clinical usefulness, patient engagement, and comfort. Remarks concerning workload and the need for protected time aligned with the existing criteria of workflow integration, institutional support, and resources and cost. No additional domains were proposed during the sessions, and participant comments did not suggest obvious omissions within the 10-criterion structure in this sample. These observations informed retention of the 10-criterion structure and its subsequent refinement ahead of prospective testing in Phase 3.

#### From Criteria to Decision Support: Operationalization of MCDA-XR

Following the refinement of the 10 criteria and their prioritization by stakeholders in Phase 2, the MCDA-XR framework was translated into an initial decision-support format for strategic preimplementation use. The final instrument organizes the framework into a dual-score structure that distinguishes between institutional priorities and current implementation conditions.

For each of the 10 criteria, stakeholders first rate strategic importance on a 5-point scale (1-5). This step captures how critical each dimension is within the local organizational context, independently of any specific XR solution. Importance ratings reflect institutional values, service objectives, and strategic goals, making explicit which considerations carry the greatest weight in adoption decisions.

Stakeholders then assess organizational readiness for each criterion on a 3-point scale (1-3), indicating the extent to which current conditions, resources, and capabilities are in place to support implementation. Readiness scores reflect practical feasibility, including workflow compatibility, staff capacity, governance arrangements, and technical preparedness.

**Figure 4.**
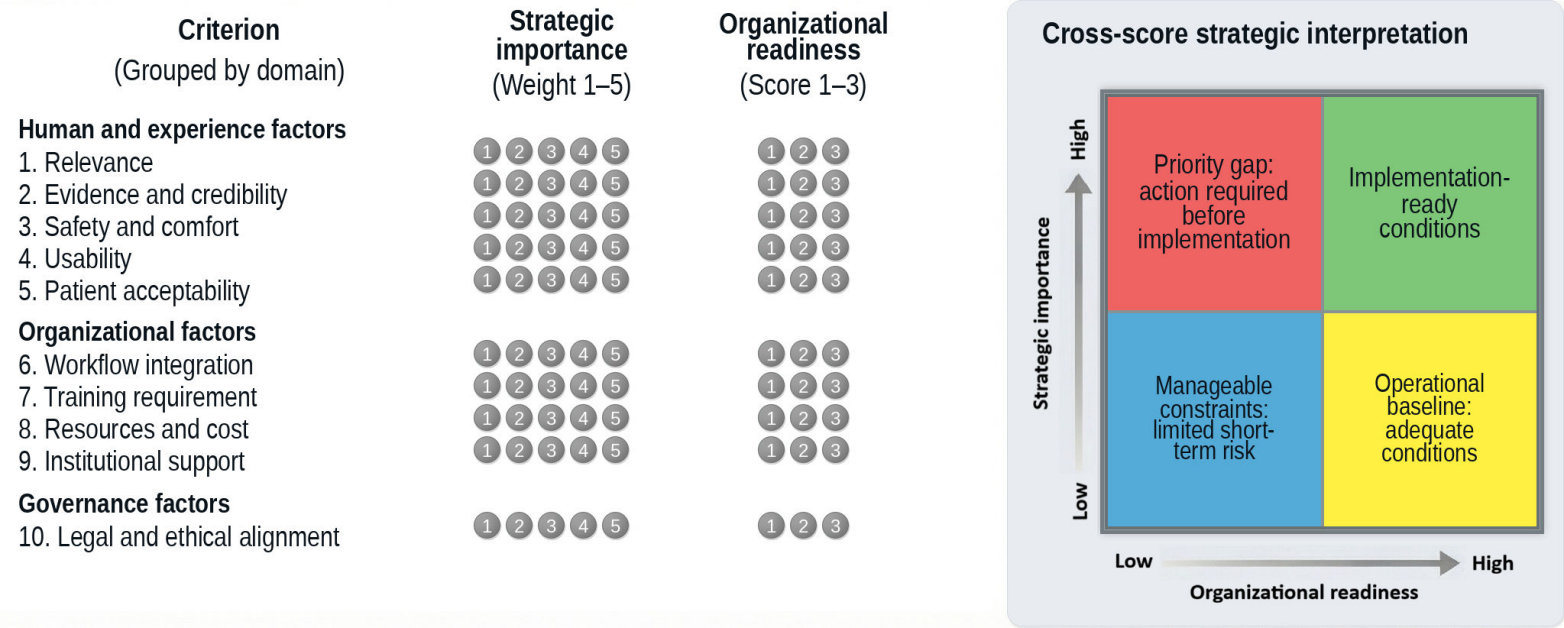
Conceptual illustration of the multicriteria decision analysis for extended reality (MCDA-XR) application.

Rather than collapsing these dimensions into a single aggregate score, MCDA-XR relies on their joint interpretation at the level of each criterion. At this stage, the matrix does not impose validated or universal decision thresholds. Instead, prioritization is based on the relative position of each criterion across the importance and readiness axes, as illustrated in Figure 4. Criteria with high importance and high readiness indicate areas that are well aligned for implementation. Criteria with high importance and low readiness identify priority gaps, where preparatory action is likely required before implementation or scale-up, such as targeted training, workflow adaptation, clarification of governance responsibilities, or allocation of additional resources. Criteria with low importance and high readiness indicate areas in which organizational conditions are already favorable, but which are not currently strategic priorities. Criteria with low importance and low readiness suggest lower-priority constraints that may be acceptable or manageable in the short term.

Figure 4 illustrates the application format of MCDA-XR. The left panel shows the evaluation scorecard where stakeholders rate the 10 criteria on strategic importance and organizational readiness. The right panel shows the cross-score interpretation matrix, where these 2 dimensions are plotted to distinguish priority gaps, defined as high importance and low readiness, from implementation-ready conditions, without collapsing the appraisal into a single aggregated score.

This importance-readiness crossing moves the framework beyond a descriptive checklist toward a structured preimplementation appraisal format. In practical terms, governance is operationalized by using this matrix to inform whether an XR initiative can proceed under current conditions, requires targeted preparation, should be adapted before deployment, or should be deferred until priority gaps are addressed. It supports informed discussion among multidisciplinary stakeholders by making trade-offs explicit and by distinguishing between aligned areas, priority gaps, and more manageable constraints under current organizational conditions. This scoring logic provides the initial operational basis of MCDA-XR, while its further refinement and prospective validation, including its translation into prioritized preparatory actions, are being addressed in Phase 3.

## Discussion

### Addressing the Strategic Gap in Digital Health Governance

This work responds to a persistent gap in how XR is governed and planned within health care organizations. Across rehabilitation, mental health, and primary care, recent reviews consistently identify determinants such as workflow fit, clinician capability, sensory tolerability, and organizational support as central to successful XR implementation [12,14,16,17]. Although these factors are well characterized, they are typically reported as descriptive findings and only rarely translated into practical evaluative frameworks that can support prospective planning. As a consequence, health care services often lack a structured means to assess feasibility before committing staff time, infrastructure, or financial resources.

MCDA-XR was developed to address this gap by organizing established behavioral, organizational, and technical determinants into a 10-criterion structure intended for strategic appraisal. The framework does not introduce new constructs. Rather, it reorganizes existing evidence into a structured basis for preimplementation appraisal that can support early planning and institutional reflection. This orientation is consistent with international guidance calling for more structured and evidence-informed approaches to the assessment and planning of digital health interventions [1,3]. It also resonates with the European Commission’s 2025 report on virtual worlds, which highlights substantial fragmentation in how safety, governance, and broader institutional considerations are currently examined, and notes the lack of cumulative, integrative approaches capable of informing policy and implementation decisions in immersive systems [47]. From this perspective, MCDA-XR should be understood as a governance-oriented multicriteria decision-support framework rather than a purely conceptual model, intended to support structured appraisal at the transition between exploratory pilots and formal implementation planning.

### Positioning MCDA-XR Within the State of the Art

The multicriteria structure of MCDA-XR differentiates it from existing approaches by linking implementation determinants to structured preimplementation appraisal. By separating strategic importance from organizational readiness, the framework invites organizations to make their priorities explicit in advance, whether related to safety, workflow integration, regulatory alignment, or resource feasibility. This distinction supports structured comparison across heterogeneous XR initiatives without collapsing decisions into a single aggregate score, a common limitation of existing appraisal approaches. At this developmental stage, however, MCDA-XR should be understood as a governance-oriented appraisal framework informed by MCDA principles, rather than as a fully specified MCDA model with validated weights, aggregation rules, or binding decision thresholds.

This design responds to limitations repeatedly identified in prior work, where evaluation tools tend to focus on a single layer of adoption. Behavioral analyses such as Chung et al [30] provide insight into individual mechanisms but offer limited guidance for organizational planning. Usability-focused studies document technical fragmentation while rarely connecting findings to governance or resource allocation decisions [13]. Broader syntheses identify recurring barriers across settings but remain largely descriptive, without translating these findings into methods for prospective prioritization [12,14,17].

Findings from Phase 2 reinforce the value of an integrative approach of this kind. Across subgroup analyses, all 10 criteria remained rated in the upper range of the scale, suggesting that the selected domains were broadly perceived as relevant across participant profiles. At the same time, Table 4 showed some variation in relative weighting. Practicing professionals gave somewhat higher ratings to workflow integration, training requirements, and institutional support, whereas postgraduate students rated relevance and usability slightly higher. Participants without prior XR experience also assigned higher ratings to evidence and credibility, legal and ethical alignment, and institutional support than those with any prior XR experience.

One possible interpretation is that stakeholders with less direct exposure to XR rely more heavily on external safeguards, including evidence, institutional endorsement, and regulatory clarity. Professionals closer to implementation, by contrast, appear to prioritize the practical conditions required to make XR workable in routine care. This reading is consistent with the experience gap described in early-stage XR evaluation, where limited first-person familiarity may influence how feasibility, risk, and implementation value are judged [48].

Rather than indicating disagreement with the underlying structure, these differences suggest that the 10 criteria were interpretable across groups, while being weighted somewhat differently according to professional role and prior familiarity with XR. The greater variation observed in domains such as institutional support and legal and ethical alignment may also reflect the influence of local organizational context, including differences in resources, governance arrangements, and implementation maturity. In this sense, MCDA-XR extends earlier integrative efforts by organizing behavioral and organizational perspectives within a structured multicriteria appraisal logic, moving beyond parallel model use toward structured decision support [31]. It thereby shifts XR evaluation from retrospective explanation toward prospective planning, in line with current calls for more structured digital health governance.

### Strengths and Limitations

A central strength of MCDA-XR lies in its dual grounding. The framework is anchored in established implementation and behavioral theory, while being iteratively refined within a living lab environment embedded in a real health care organization. This setting exposed the framework to competing clinical priorities, resource constraints, and governance requirements that are often underrepresented in conceptual or purely theoretical work. As a result, the criteria and their operational definitions are shaped by conditions encountered during actual planning and decision-making, rather than by idealized assumptions about technology adoption.

A second strength is the participatory construction process. By engaging a heterogeneous set of participants spanning clinical practice, operational roles, and postgraduate training, the framework integrates multiple perspectives during its formative development. The generally high importance ratings across domains, together with the absence of additional determinants proposed by participants, suggest that the 10-criterion structure provides a coherent representation of key XR implementation considerations within this formative stage. These findings should not, however, be interpreted as evidence that the framework is exhaustive or fully generalizable beyond the present developmental context.

Several limitations should also be acknowledged. This study focuses on framework development and formative refinement. While participants supported the relevance and interpretability of the criteria, the readiness scoring component has not yet been tested prospectively. In addition, the Phase 2 sample was heterogeneous and included both practicing professionals and postgraduate psychology students, as well as participants with limited or no prior XR exposure. Postgraduate participants were included because behavioral science and human-technology interaction are central to the framework, but not all participants routinely engage in organizational decision-making. This might limit the extent to which the resulting importance ratings can be interpreted as representative of organizational decision-making priorities and may introduce potential bias related to participant composition, subjective scoring, and local organizational context. The Phase 2 findings should therefore be interpreted as formative evidence of relevance and interpretability, rather than as proof of generalizability, definitive governance weights, or predictive validity.

Although all participants received a common introductory briefing and live demonstration before rating, this level of exposure was necessarily brief and should be interpreted as experiential familiarization rather than sustained experience of XR use in clinical practice. For participants without prior XR experience, the resulting ratings should therefore still be understood as anticipatory appraisals rather than as judgments grounded in direct implementation experience, a limitation consistent with the experience gap described in early-stage XR evaluation [48].

The LLM-assisted extraction workflow also entails methodological limitations. Although the extracted determinants were manually checked against the sources and documented in the appendices, the process did not include independent duplicate extraction or a formal reliability assessment. Classification and synthesis therefore required interpretive judgment. The resulting framework should be read as a transparent, human-verified synthesis, rather than as an automatically reproducible extraction procedure.

The framework remains sensitive to technological and regulatory evolution. Ongoing changes in XR hardware, software platforms, and regulatory interpretations may require periodic revision of technical descriptors. This reinforces the need to treat readiness assessment as an iterative process, embedded within routine governance, rather than as a one-off evaluation.

These limitations will be addressed in Phase 3, which will examine the practical utility, interpretability, and implementation relevance of MCDA-XR in real-world clinical settings, including how organizational readiness profiles inform preparatory planning during implementation.

### Strategic Implications for Research and Practice

For researchers, MCDA-XR provides a structured way to characterize implementation contexts using a shared analytical language. By organizing behavioral, organizational, and technical determinants within a single decision framework, it facilitates comparison across studies and supports cumulative knowledge development beyond isolated case reports.

For health care organizations, the framework has practical relevance as a structured planning aid. MCDA-XR enables teams to assess initiatives against explicit criteria, make trade-offs visible, and identify areas where additional preparation or institutional support may be needed before scaling, for example, when limited training capacity, workflow misalignment, or weak institutional support emerge as priority gaps requiring targeted preparatory action. Rather than replacing professional judgment, it offers a transparent structure to support collective decision-making and align adoption choices with organizational priorities.

More broadly, MCDA-XR contributes to a shift away from ad hoc decision-making toward more deliberate strategic planning in digital health. By explicitly linking adoption decisions to organizational readiness and governance considerations, the framework supports a more sustainable and defensible approach to innovation, particularly in resource-constrained health care settings.

At the same time, the broader applicability of MCDA-XR should still be interpreted with caution. Framework development was anchored at BSA, and the formative work in Phase 2 included participants from both BSA and other hospital, primary care, and academic settings. Even so, the current weighting logic and interpretive use may not transfer directly to organizations working under different governance structures, reimbursement models, regulatory conditions, or levels of digital maturity. Its transferability therefore appears more plausible at the structural level of the 10 criteria than at the level of local scoring patterns or implementation priorities. Phase 3 is intended to examine this question further through prospective application in real-world implementation settings.

### Conclusion

XR introduces forms of interaction, sensory engagement, and organizational demand that are not adequately addressed by conventional digital health planning approaches. Although the determinants influencing XR adoption are increasingly well characterized, health care organizations still lack practical, governance-oriented tools to support structured decision-making before implementation. This study responds to that gap through the development of MCDA-XR.

By distinguishing strategic importance from organizational readiness across a shared set of empirically grounded criteria, MCDA-XR is intended to help decision-makers identify priority gaps, assess feasibility, and surface governance challenges before committing institutional resources. In this way, the framework shifts evaluation away from descriptive feasibility assessments toward a more transparent and action-oriented planning process, grounded in organizational capacity and strategic intent.

The work presented here establishes the conceptual architecture and initial operational logic of MCDA-XR. Phase 3 will test and refine the framework in real-world implementation settings, including how crossed importance-readiness profiles can be interpreted and translated into prioritized preparatory actions. If supported, MCDA-XR may contribute to more deliberate, accountable, and sustainable approaches to digital health governance, particularly within resource-constrained healthcare systems.

## Supplementary material

10.2196/89801 Multimedia Appendix 1 Comparative overview of extended reality implementation studies included in the Phase 1 evidence review and the implementation determinants reported in each study.

10.2196/89801 Multimedia Appendix 2 Large language model–assisted determinant extraction protocol and extraction matrix used to identify barriers, facilitators, and requirements for extended reality implementation.

10.2196/89801 Multimedia Appendix 3 Thematic synthesis of extracted determinants and supporting evidence for the 10 multicriteria decision analysis for extended reality criteria.

10.2196/89801 Multimedia Appendix 4 Theoretical and empirical alignment of the multicriteria decision analysis for extended reality criteria with non-adoption, abandonment, scale-up, spread, sustainability, capability, opportunity, motivation-behaviour, technology acceptance model, and Reporting of Applications, Technologies, and Experiments in Extended Reality.

10.2196/89801 Multimedia Appendix 5 Participant demographics and professional roles for the 33 stakeholders included in the Phase 2 construction and refinement sessions.

## Abbreviations

AR: augmented reality
BSA: Badalona Serveis Assistencials
CFIR: consolidated framework for implementation research
COM-B: capability, opportunity, motivation-behaviour
GDPR: General Data Protection Regulation
LLM: large language model
LOPDGDD: Organic Law on Protection of Personal Data and Guarantee of Digital Rights
MCDA: multicriteria decision analysis
MCDA-XR: multicriteria decision analysis for extended reality
MR: mixed reality
NASSS: non-adoption, abandonment, scale-up, spread, sustainability
RATE-XR: Reporting of Applications, Technologies, and Experiments in Extended Reality
TAM: technology acceptance model
UOC: University of Catalonia
UOC: Open University of Catalonia
VR: virtual reality
WHO: World Health Organization
XR: extended reality

## References

[1] (2019). WHO guideline: recommendations on digital interventions for health system strengthening. World Health Organization.

[2] (2021). Global strategy on digital health 2020–2025. World Health Organization.

[3] (2025). Virtual worlds: how do they affect our health and well-being?.

[4] Spiegel BMR, Rizzo A, Persky S (2024). What is medical extended reality? A taxonomy defining the current breadth and depth of an evolving field. J Med Ext Real.

[5] Slater M, Sanchez-Vives MV (2016). Enhancing our lives with immersive virtual reality. Front Robot AI.

[6] Riva G, Wiederhold BK, Mantovani F (2019). Neuroscience of virtual reality: from virtual exposure to embodied medicine. Cyberpsychol Behav Soc Netw.

[7] Slater M (2009). Place illusion and plausibility can lead to realistic behaviour in immersive virtual environments. Philos Trans R Soc Lond B Biol Sci.

[8] Makransky G, Petersen GB (2021). The Cognitive Affective Model of Immersive Learning (CAMIL): a theoretical research-based model of learning in immersive virtual reality. Educ Psychol Rev.

[9] Grassini S, Laumann K, Parlangeli O, Marti P, Parlangeli O Immersive visual technologies and human health.

[10] Lassen KL, Hermander K, Jildenstål P (2025). Virtual reality is safe and can reduce in-hospital anxiety and pain: a systematic review with meta-analyses and trial sequence analyses. Eur J Pain.

[11] Zuo G, Wang R, Wan C, Zhang Z, Zhang S, Yang W (2024). Unveiling the evolution of virtual reality in medicine: a bibliometric analysis of research hotspots and trends over the past 12 years. Healthcare (Basel).

[12] Kouijzer M, Kip H, Bouman YHA, Kelders SM (2023). Implementation of virtual reality in healthcare: a scoping review of barriers and facilitators. Front Digit Health.

[13] Zhang T, Booth R, Jean-Louis R (2020). A primer on usability assessment approaches for health-related applications of virtual reality. JMIR Serious Games.

[14] Lie SS, Helle N, Sletteland NV, Vikman MD, Bonsaksen T (2023). Implementation of virtual reality in health professions education: scoping review. JMIR Med Educ.

[15] Morgan JW, Patel RA, Campbell S (2025). Practical considerations of clinical XR (AR/VR) deployments. Front Virtual Real.

[16] Glegg SMN, Levac DE (2018). Barriers, facilitators and interventions to support virtual reality implementation in rehabilitation: a scoping review. PM R.

[17] Elser A, Lange M, Kopkow C, Schäfer AG (2024). Barriers and facilitators to the implementation of virtual reality interventions for people with chronic pain: scoping review. JMIR XR Spat Comput.

[18] Michie S, van Stralen MM, West R (2011). The behaviour change wheel: a new method for characterising and designing behaviour change interventions. Implement Sci.

[19] Greenhalgh T, Abimbola S (2019). The NASSS Framework - a synthesis of multiple theories of technology implementation. Stud Health Technol Inform.

[20] Damschroder LJ, Aron DC, Keith RE, Kirsh SR, Alexander JA, Lowery JC (2009). Fostering implementation of health services research findings into practice: a consolidated framework for advancing implementation science. Implement Sci.

[21] Nilsen P (2015). Making sense of implementation theories, models and frameworks. Implement Sci.

[22] Lewis CC, Frank HE, Cruden G (2024). A research agenda to advance the study of implementation mechanisms. Implement Sci Commun.

[23] Vlake JH, Drop DLQ, Van Bommel J (2024). Reporting guidelines for the early-phase clinical evaluation of applications using extended reality: RATE-XR qualitative study guideline. J Med Internet Res.

[24] Shin HD, Hamovitch E, Gatov E (2025). The NASSS (Non-Adoption, Abandonment, Scale-Up, Spread and Sustainability) framework use over time: a scoping review. PLOS Digit Health.

[25] Holden RJ, Karsh BT (2010). The technology acceptance model: its past and its future in health care. J Biomed Inform.

[26] Venkatesh V, Thong JYL, Xu X (2012). Consumer acceptance and use of information technology: extending the unified theory of acceptance and use of technology. MIS Q.

[27] Venkatesh V, Morris MG, Davis GB, Davis FD (2003). User acceptance of information technology: toward a unified view. MIS Q.

[28] Cane J, O’Connor D, Michie S (2012). Validation of the theoretical domains framework for use in behaviour change and implementation research. Implement Sci.

[29] Friedman C, Rubin J, Brown J (2015). Toward a science of learning systems: a research agenda for the high-functioning Learning Health System. J Am Med Inform Assoc.

[30] Chung OS, Robinson T, Johnson AM (2021). Implementation of therapeutic virtual reality into psychiatric care: clinicians’ and service managers’ perspectives. Front Psychiatry.

[31] Pereira Guerreiro M, Félix IB, Tomé M (2025). Protocol for a usability and pilot implementation study of a digital medical device to assess pain in non-verbal people with dementia in Portuguese residential care facilities. Digit Health.

[32] Deason JP, Adams SJ, Rahman A, Lovo S, Mendez I (2025). A technology selection tool applying multiple criteria decision analysis for virtual care implementation. Mayo Clin Proc Digit Health.

[33] Chung OS, Dowling NL, Brown C (2023). Using the Theoretical Domains Framework to inform the implementation of therapeutic virtual reality into mental healthcare. Adm Policy Ment Health.

[34] Alrashidi M, Tomlinson RJ, Buckingham G, Williams CA (2025). Virtual reality current use, facilitators and barriers to implementation in paediatric physiotherapy: cross-sectional online survey of UK paediatric physiotherapists. Disabil Rehabil Assist Technol.

[35] Felnhofer A, Pfannerstill F, Gänsler L (2025). Barriers to adopting therapeutic virtual reality: the perspective of clinical psychologists and psychotherapists. Front Psychiatry.

[36] Schreiter M, Hennrich J, Wolf AL, Eymann T (2025). The influence of previous experience on virtual reality adoption in medical rehabilitation and overcoming knowledge gaps among health care professionals: qualitative interview study. J Med Internet Res.

[37] Sarkar U, Lee JE, Nguyen KH, Lisker S, Lyles CR (2021). Barriers and facilitators to the implementation of virtual reality as a pain management modality in academic, community, and safety-net settings: qualitative analysis. J Med Internet Res.

[38] Skryabina E, Reedy G, Brock L (2023). What healthcare educators know about extended reality (XR). King’s College London, UCLPartners.

[39] Abbas JR, Gantwerker E, Volk M (2024). Describing, evaluating, and exploring barriers to adoption of virtual reality: an international modified Delphi consensus study involving clinicians, educators, and industry professionals. J Med Ext Real.

[40] Kouijzer MTE, Kip H, Kelders SM, Bouman YHA (2024). The introduction of virtual reality in forensic mental healthcare - an interview study on the first impressions of patients and healthcare providers regarding VR in treatment. Front Psychol.

[41] Lurtz J, C Sauter T, Jacob C (2024). Factors impacting the adoption and potential reimbursement of a virtual reality tool for pain management in Switzerland: qualitative case study. JMIR Hum Factors.

[42] Shiner CT, Croker G, McGhee J, Faux SG (2024). Perspectives on the use of virtual reality within a public hospital setting: surveying knowledge, attitudes, and perceived utility among health care professionals. BMC Digit Health.

[43] Terkildsen MD, Bollerup S, Palmhøj C, Jensen LG, Lou S (2024). How institutional logics shape the adoption of virtual reality in mental health care: a qualitative study. Digit Health.

[44] Lattré T, Hagert E, Suero-Pineda A, Decramer A (2025). Immersive virtual reality in hand therapy - an international survey of clinical integration. Hand Surg Rehabil.

[45] Mondal H, Mondal S (2025). Adopting augmented reality and virtual reality in medical education in resource-limited settings: constraints and the way forward. Adv Physiol Educ.

[46] Braun V, Clarke V (2006). Using thematic analysis in psychology. Qual Res Psychol.

[47] Barreda-Ángeles M, Améndola S, Silva C (2025). Virtual Worlds and Well-Being: Current Research and Future Directions.

[48] Ferrer Costa J, Armayones Ruiz M (2026). Enhancing the predictive value of formative evaluation in extended reality adoption: addressing the experience gap. JMIR Form Res.

