# Supplementary material for: The Multicriteria Decision Analysis for Extended Reality (MCDA-XR) Governance Framework for Health Care Adoption: Mixed Methods Development Study
Source: J Med Internet Res. 2026 Jul 31;28:e89801. doi: 10.2196/89801 (PMC13430000; doi:10.2196/89801)
Supplement: Multimedia Appendix 1 [file jmir-v28-e89801-s001.pdf]

Multimedia Appendix 1. Comparative Overview of XR Implementation Studies and Their Reported Implementation Determinants

This file compiles the primary studies included in the comparative overview and summarises the implementation determinants described in each publication. It provides a consolidated view of the real-world barriers, facilitators, and requirements documented across XR health research. The table serves as the empirical foundation for subsequent determinant extraction and synthesis.

Group I: Studies with a Primary Focus on Methodological and Usability Factors

Focus: Technical performance, safety, and interface interaction. These studies highlight that while usability is well-mapped, it does not predict organisational adoption.

Reference / Year	Main Focus	Methodology	Frameworks / Theoretical Base	Key Findings / Contributions	Limitations / Remaining Gap	Relevance to MCDA-XR
Zhang et al., 2020 [13]	Usability assessment approaches in VR for health	Narrative review; methodological synthesis	Human–computer interaction and human-factors taxonomies	Identifies six categories of VR usability evaluation; shows large variability in metrics, protocols, and tools; emphasises lack of standardisation	No organisational or behavioural analysis; not an implementation model	Supports MCDA-XR usability and safety criteria; reveals fragmentation requiring structured XR-specific evaluation
Morgan et al., 2025 [15]	Practical considerations for clinical XR deployment	Narrative synthesis; engineering and clinical perspectives	Human-factors principles; engineering considerations; discussion aligned with RATE-XR concepts	Highlights challenges in ergonomics, tracking limits, hardware, data governance, workflow disruption, training burden, economic fit	Non-empirical; generalist; no prioritisation	Directly informs MCDA-XR on safety, usability, workflow fit, infrastructure, governance, and regulatory alignment

Group II: Studies Prioritising Behavioural and Individual Frameworks

Focus: Clinician and patient acceptance, beliefs, skills, and motivation. These studies provide deep insight into the "human factor" (COM-B/TDF) but often overlook system-level constraints.

Reference / Year	Main Focus	Methodology	Frameworks / Theoretical Base	Key Findings / Contributions	Limitations / Remaining Gap	Relevance to MCDA-XR
Glegg & Levac, 2018 [16]	Barriers and facilitators in rehab VR	PRISMA scoping review	TDF; KT concepts	Determinants span knowledge, skills, environment, social influences; KT strategies rarely tested	Pre-2020; descriptive; limited org/system data	Foundational behavioural determinants feeding capability and opportunity criteria
Chung et al., 2022 [30]	Behavioural drivers of VR implementation in psychiatry	Qualitative interviews; TDF-based coding	TDF and COM-B	Identifies beliefs, constraints, confidence, identity, emotions; behavioural bottlenecks central to early adoption	Single service; no organisational framework	Direct input to COM-B-derived MCDA-XR domains
Chung et al., 2023 [33]	TDF-based diagnostic and reflection toolkit	Semi-structured interviews; TDF to ERIC strategy mapping	TDF (coding); ERIC (strategy mapping), COM-B (mentioned conceptually)	Produces behavioural and contextual map linked to strategies; basis for reflection tools	No validation; no scoring; no org/system layer	Provides raw diagnostic logic MCDA-XR translates into measurable criteria
Alrashidi et al., 2024 [34]	VR use and barriers in paediatric physiotherapy	Cross-sectional survey (n=81)	ADOPT-VR (acceptance/behaviour constructs)	Very low use; barriers include cost, setup, support, evidence, training; facilitators include support time and patient motivation	Cross-sectional; perceived data only	Quantitative support for capability, opportunity, evidence, resources, training criteria
Elser et al., 2024 [17]	Barriers/facilitators of VR in chronic pain	PRISMA-ScR review; TDF coding	TDF (COM-B aligned)	65 determinants; dominant environmental, skill, belief barriers; minimal organisational data	Descriptive; weak org/system evidence	Reinforces behavioural determinants; shows organisational data gap filled by MCDA-XR
Felnhofer et al., 2025 [35]	Barriers to VR adoption among clinical psychologists and psychotherapists	Cross-sectional survey (n=277)	None; inductive categories	High interest but strong hesitancy; training gaps, safety, cost, time burden	No organisational determinants; self-report	Supports safety, capability, opportunity, evidence perceptions
Schreiter et al., 2025 [36]	Influence of past VR experience	Qualitative interviews	TAM and COM-B	Prior experience predicts perceived usefulness, lower anxiety, stronger intention; lack of familiarity hinders adoption	Individual level only; small sample	Supports familiarity, training, perceived usefulness, capability criteria

Group III: Studies Prioritising Organisational and System-Level Determinants

Focus: Workflow, resources, infrastructure, and policy. These studies highlight the structural barriers that typically halt implementation (NASSS/CFIR).

Reference / Year	Main Focus	Methodology	Frameworks / Theoretical Base	Key Findings / Contributions	Limitations / Remaining Gap	Relevance to MCDA-XR
Sarkar et al., 2021 [37]	VR pain-management implementation across diverse settings	Semi-structured interviews; CFIR coding	CFIR	Identifies workflow complexity, staff time, training needs, cultural mismatch, low digital literacy, reimbursement barriers	Regional sample; no prioritisation	Strong alignment with workflow fit, staff burden, training, equity, reimbursement
Kouijzer et al., 2023 [12]	VR implementation across healthcare	PRISMA scoping review	NASSS/CFIR concepts (discussion)	69 barriers, 53 facilitators; shows inconsistent framework use; highlights gaps in system-level evidence	Descriptive; mixed evidence; no evaluative model	Broadest map of organisational/system determinants; supports complexity, readiness, safety, workflow domains

UCL & KCL, 2023 [38]	XR adoption in NHS education	Rapid review and interviews across 13 Trusts	None (practical domains)	Barriers: cost, IT constraints, low digital readiness, workforce capacity; facilitators: leadership, access, procurement networks	Grey literature; London-specific	Reinforces cost, workforce, infrastructure, leadership, procurement criteria
Abbas et al., 2024 [39]	Consensus on evaluation domains and barriers in VR healthcare education	Modified Delphi	None. Domains generated inductively.	Cost is top barrier; consensus on evaluating knowledge, usability, retention, organisational outcomes	Educational scope only; no implementation analysis	Confirms cost and need for structured evaluation criteria
Kouijzer et al., 2024 [40]	Early impressions of VR in forensic mental health	Interviews with patients and providers	None; NASSS/CFIR mentioned in the discussion.	Emphasises psychological safety, realism limits, usability concerns, role clarity, pathway integration	Small exploratory sample; single setting; neutral VR content only.	Supports patient safety, capability, training, workflow fit, organisational support
Lurtz et al., 2024 [41]	Adoption and reimbursement of VR in emergency care	Semi-structured interviews; framework-guided	Consolidated Framework for eHealth Adoption	Facilitators: leadership, innovation culture, engagement; barriers: workload, time, reimbursement uncertainty	Small, role-imbalanced sample	Key evidence for workflow fit, resources, leadership, reimbursement, regulatory alignment
Shiner et al., 2024 [42]	Professional knowledge, attitudes, perceived utility	Cross-sectional survey (n=137)	None	Very limited experience; strong perceived utility; barriers include cost, IT support, skills, time, space, evidence	Single hospital; descriptive	Supports capability, opportunity, resources, technical support, infrastructure, evidence credibility
Terkildsen et al., 2024 [43]	Institutional logics in mental-health VR	Qualitative comparative case study	Institutional Logics	Identifies competing professional vs diffusion logics; misalignment creates friction	Single site; interpretive	Supports need for multi-stakeholder weighting, alignment of organisational and clinical priorities
Lattré et al., 2025 [44]	VR integration in hand therapy	International online survey	None	Very low use; motivation benefits; major barriers: cost, time, evidence, ergonomics; high protocol variability	No organisational/behavioural framework; specific to hand therapy	Supports cost, usability, resources, technical fit, training; highlights organisational-readiness gap
Mondal & Mondal, 2025 [45]	XR adoption in resource-limited medical-education settings	Narrative perspective	None	Highlights infrastructure gaps, device cost, low literacy, cultural resistance, policy barriers; proposes low-cost strategies	No empirical data; education-only	Reinforces contextual-resource, cost, literacy, equity, sociocultural-fit criteria

Group IV: Integrative and Methodological Precedents from Digital Health

Focus: Studies that successfully combined frameworks or used MCDA, demonstrating the feasibility of the approach MCDA-XR proposes.

Reference / Year	Main Focus	Methodology	Frameworks / Theoretical Base	Key Findings / Contributions	Limitations / Remaining Gap	Relevance to MCDA-XR
Deason et al., 2025 [32]	MCDA tool for telehealth technology selection	Co-design; attribute definition; scoring and weighting	MCDA aligned with ISPOR guidance	Demonstrates feasibility of MCDA for health-technology decisions; improves transparency and comparability	Not XR; no behavioural or organisational determinants	Validates MCDA as core methodological backbone for MCDA-XR
Pereira Guerreiro et al., 2025 [31]	Parallel behavioural and organisational analysis in dementia care	Mixed-methods protocol; ISO-guided usability, COM-B/TDF coding, NASSS analysis	NASSS; COM-B; TDF; ISO 9241-11	Shows NASSS and COM-B produce complementary, non-overlapping insights; offers rare combined application	Protocol only; non-XR; no scoring or model	Strong conceptual support for MCDA-XR; confirms rationale for combining behavioural and organisational determinants
