# Supplementary material for: The Multicriteria Decision Analysis for Extended Reality (MCDA-XR) Governance Framework for Health Care Adoption: Mixed Methods Development Study
Source: J Med Internet Res. 2026 Jul 31;28:e89801. doi: 10.2196/89801 (PMC13430000; doi:10.2196/89801)
Supplement: Multimedia Appendix 2 [file jmir-v28-e89801-s002.pdf]

Multimedia Appendix 2. Verbatim Determinant Extraction Matrix With MCDA-XR Classification

This appendix provides the methodological audit trail supporting Phase 1 of the study and is organised into two parts.

Part A presents the LLM-assisted extraction protocol used to generate the determinant matrix. This includes the rule-based extraction framework applied within a project-specific ChatGPT configured environment, together with the manual verification procedure used to confirm section eligibility, attribution accuracy, verbatim fidelity, and criterion assignment.

Part B presents the extraction matrix containing the implementation determinants identified in each included study. Determinants were restricted to sections in which the study authors reported their own findings, analysis, or methodological reflections relevant to implementation. Each determinant was classified as a barrier, facilitator, or requirement and mapped to one of the ten MCDA-XR criteria.

Together, these materials provide a transparent link between the source publications, the extracted implementation determinants, and the construction of the MCDA-XR criteria.

Part A. LLM-assisted extraction protocol

Overview

The extraction process was conducted in ChatGPT, using OpenAI GPT-family models within a project-specific configured environment. Methodological consistency was ensured through a predefined rule-based instruction set rather than reliance on a specific model release.

The model was used to support the structured identification, extraction, and provisional classification of implementation determinants from uploaded study documents. It was explicitly constrained to avoid unsupported inference, synthesis, or generation of new content, and all outputs were subsequently reviewed manually against the original source documents.

Source restriction

The model was instructed to use only the uploaded PDF as the source of information for each study. No external knowledge or background information was permitted. All extracted determinants therefore had to remain directly traceable to the source document.

Section restriction

Extraction was limited to sections representing the authors' own contribution, including Results, Discussion, Conclusion, and relevant subsections such as Limitations, Challenges, Gaps, Lessons learned, Recommendations, or methodological subsections when these described implementation constraints, requirements, or practical observations arising from the study itself.

The model was explicitly instructed not to extract determinants from the Abstract, Introduction, Background, Related work, or similar contextual sections, as these often contain framing statements or literature summaries rather than study-generated implementation findings. Section headers were identified directly from the PDF and recorded for each extracted determinant to preserve traceability.

Attribution filter

Statements explicitly attributed to another study or external source were excluded from extraction. This applied to formulations such as "previous studies have shown" or "X et al. found," where the determinant did not originate from the authors' own findings or analysis.

Determinant definition

A determinant was defined as an explicit statement presented by the study authors as a barrier, facilitator, or requirement relevant to implementation. Only determinants stated explicitly in eligible sections were retained. The model was not permitted to infer determinants from general discussion or extrapolate beyond the source text.

Granularity and verbatim rules

Determinants were extracted at the highest level of specificity available in the source text. When a broader statement contained multiple specific determinants, each was extracted separately. Determinants were retained in verbatim form whenever possible, with only minor typographic normalisation permitted to correct PDF artefacts such as spacing, line breaks, or punctuation inconsistencies. No paraphrasing, summarisation, or conceptual reformulation was allowed.

Classification rule

After extraction, each determinant was provisionally assigned to one of the ten predefined MCDA-XR criteria: Relevance, Evidence and credibility, Safety and comfort, Usability, Integration in workflow, Resources and cost, Training requirement, Patient acceptability, Institutional support, and Legal and ethical alignment. Where classification was potentially ambiguous, the closest fitting criterion was selected and reviewed during manual verification.

Absolute prohibitions

The model was explicitly instructed not to extract from non-eligible sections, include determinants attributed to prior studies, infer or synthesise beyond the literal source text, merge multiple determinants into a broader category, or create new evaluative categories beyond the ten predefined MCDA-XR criteria.

Manual verification

All extracted determinants underwent manual verification against the original PDF by the research team. This review confirmed section eligibility, authorship attribution, exclusion of externally sourced statements, verbatim fidelity apart from minor typographic normalisation, and appropriateness of criterion assignment. Items were corrected or removed when necessary. The final extraction matrix therefore reflects human-validated source-level evidence rather than autonomous model-generated output.

Full prompt - Criteria Extractor

The complete prompt and project instruction set used in the configured environment are provided below in full to support transparency and reproducibility.

You are assisting with the structured extraction and classification of implementation determinants to support the development of the MCDA-XR framework for the adoption of extended reality technologies in healthcare.

For each XR implementation study provided as a PDF or pasted text, extract only the implementation determinants generated by the study itself, classify them into the 10 MCDA-XR criteria, and present them in a structured, auditable format.

1. Source fidelity

Use only the content contained in the provided study document.

Do not use external knowledge.
Do not introduce assumptions beyond the literal text.
All extracted content must be directly traceable to the source document.

2. Section restriction

Do not extract determinants from:

Abstract
Introduction
Background
Related work or literature review sections
Any paragraph that cites another study as the source of the statement

Extract determinants only from sections representing the authors' own contribution, including:

Results
Discussion
Conclusion
Subsections such as Limitations, Challenges, Gaps, Barriers, Lessons learned, Recommendations
Methods, only when describing practical constraints, implementation requirements, or limitations identified by the authors

3. Section boundary identification

Identify the document's section titles and structure directly from the PDF.

Use the section header exactly as it appears in the document to label each extracted determinant.

4. Attribution filter

Exclude any determinant explicitly attributed to another study.

Examples of excluded formulations include:

"previous studies have shown…"
"X et al. found…"
"according to prior research…"

Only extract determinants generated by the authors of the current study.

5. Determinant definition

A determinant is any explicit statement presented by the authors as:

a barrier
a facilitator
a requirement

Determinants must be explicitly stated.
Do not infer determinants from context or general discussion.

6. Granularity requirement

Extract determinants at the highest level of specificity available.

Do not extract broad or aggregated statements when more specific determinants are described.

If multiple determinants appear within a single sentence or paragraph, extract each one separately.

7. Verbatim integrity

Extract determinants using the exact wording from the document whenever possible.

Minor typographic normalisation is allowed only to correct PDF artefacts, such as:

spacing errors
line breaks

punctuation inconsistencies

Do not paraphrase, summarise, or generalise.

8. Mandatory classification into MCDA-XR criteria

Assign each determinant to one of the following criteria:

Relevance
Evidence and credibility
Safety and comfort
Usability
Integration in workflow
Resources and cost
Training requirement
Patient acceptability
Institutional support
Legal and ethical alignment

If classification is ambiguous, assign the closest criterion.
Provide a brief note only when necessary to clarify the decision.

9. Output requirements

Produce two outputs:

Part 1. Raw determinant list

A list of extracted determinants, one determinant per item, using verbatim wording.

Part 2. MCDA-XR classification table

One determinant per row with the following columns:

Study (Author, Year)
Type (Barrier / Facilitator / Requirement)
Raw Determinant (Verbatim)
MCDA-XR Criterion
Notes, if needed
Section (as identified in the PDF)

The section field must match the original section header exactly.

10. Extraction process

For each document:

Read the entire document
Identify the section structure
Apply section restriction
Apply attribution filter
Extract determinants at maximum specificity
Generate the raw determinant list
Generate the classification table with section traceability

11. Absolute prohibitions

Do not:

extract from excluded sections
include determinants attributed to other studies
infer, synthesise, or reinterpret content
merge multiple determinants into a single item
create new categories beyond the predefined MCDA-XR criteria
include statements not explicitly framed as determinants

12. Additional notes

If PDF extraction introduces formatting artefacts, these may be corrected without altering the wording.

Author-defined labels appearing in figures may be retained when they are directly relevant to implementation determinants.

Notes should be included only when necessary, mainly to indicate:

ambiguity in classification
minor typographic normalisation

Otherwise, leave the Notes field empty.

Part B. Extraction matrix

Study (Author, Year)	Type (Barrier / Facilitator / Requirement)	Raw Determinant	MCDA-XR Criterion	Notes	Section
Abbas et al., 2024	Requirement	"A VR experience should be defined by its interplay between hardware and software."	Evidence & credibility	Definition requirement supports interpretability of evidence	Results, Area of interest 1—describing VR technology (first research question)
Abbas et al., 2024	Requirement	"mixed methods should be utilized when evaluating educational tools"	Evidence & credibility	Evaluation design guidance	Results, Area of interest 2—educational evaluation methodology (second research question)
Abbas et al., 2024	Requirement	"studies that should measure educational outcomes over multiple time periods in order to determine retention of learning"	Evidence & credibility	Timing is general, not fixed	Results, Area of interest 2—educational evaluation methodology (second research question)
Abbas et al., 2024	Requirement	"the participant's knowledge should be measured when evaluating the learning content of a VR training tool."	Evidence & credibility	Outcome measurement requirement	Results, Area of interest 3—research outcome measures (third research question)
Abbas et al., 2024	Requirement	"usability, skill performance retention, and organizational outcomes reached the level for consensus inclusion"	Evidence & credibility	High level summary of required outcomes	Results, Area of interest 3—research outcome measures (third research question)
Abbas et al., 2024	Requirement	"any literature description of novel health care VR should be described using the hardware and software interplay, and not by hardware, or software alone."	Evidence & credibility	Reporting requirement	Discussion
Abbas et al., 2024	Requirement	"content validity was mandatory"	Evidence & credibility	Shortened phrase appears	Discussion
Abbas et al., 2024	Requirement	"mixed-methods approach when evaluating novel VR training."	Evidence & credibility	Evaluation approach	Discussion
Abbas et al., 2024	Requirement	"it is of vital importance that the presented barriers to technology adoption are considered."	Institutional support	Broad, but explicitly frames adoption planning	Discussion
Abbas et al., 2024	Requirement	"technology adoption within any health care system is reliant on sustainable business models."	Resources & cost	Relates to sustainability and financing	Discussion
Abbas et al., 2024	Requirement	"These priorities are centered on three main areas including cost, volume of educational content, accessibility, and comfort"	Relevance	"Priorities" are adoption drivers; criterion split across multiple domains	Discussion
Abbas et al., 2024	Requirement	"Virtual Environment (a digital simulated space which can be real or fictional)."	Evidence & credibility	Terminology standardisation	Table 3, Describing VR technology
Abbas et al., 2024	Requirement	"Interactive (e.g., buttons, choices, or fully-interactive elements)."	Usability	Interaction modality	Table 3, Describing VR technology
Abbas et al., 2024	Requirement	"Simulation (imitation of an environment, object or process)."	Evidence & credibility	Definition for reporting	Table 3, Describing VR technology
Abbas et al., 2024	Requirement	"Environment (e.g., an operating theatre, emergency room, or fictional area)."	Relevance	Context specificity	Table 3, Describing VR technology
Abbas et al., 2024	Requirement	"Immersion (relating to the sensations that a user experiences)."	Safety & comfort	Sensory exposure and tolerability link	Table 3, Describing VR technology
Abbas et al., 2024	Requirement	"Interactive Simulation (an imitated environment, object, or process which can be interacted with)."	Usability	Interaction requirement	Table 3, Describing VR technology
Abbas et al., 2024	Requirement	"3D (digital assets or environments with the three spatial dimensions of height, width, and depth)."	Usability	Spatial representation	Table 3, Describing VR technology
Abbas et al., 2024	Requirement	"Experience (an activity or event that the user experiences which may be passive or active)."	Patient acceptability	Experience framing	Table 3, Describing VR technology
Abbas et al., 2024	Requirement	"Technology (including hardware, software, or both)."	Evidence & credibility	Reporting completeness	Table 3, Describing VR technology
Abbas et al., 2024	Requirement	"Head Mounted Display (a type of VR device worn on a user's head which displays media via screens to each, or both eyes)."	Resources & cost	Hardware requirement implies procurement	Table 3, Describing VR technology
Abbas et al., 2024	Requirement	"Outcomes should be measured again after a period to assess retention."	Evidence & credibility	Retention measurement	Table 3, Educational evaluation methodology

Abbas et al., 2024	Requirement	“Mixed methods research (qualitative and quantitative data) is the best approach when evaluating VR education.”	Evidence & credibility	Evaluation approach	Table 3, Educational evaluation methodology
Abbas et al., 2024	Requirement	“VR educational modalities should be focused around Kirkpatrick learning level 3 as described as the transfer of knowledge, skills and abilities.”	Relevance	Focus on transfer outcomes	Table 3, Educational evaluation methodology
Abbas et al., 2024	Requirement	“Usability should be measure by validated questionnaires such as the widely accepted System Usability Scale.”	Usability	Validated usability assessment	Table 3, Educational evaluation methodology
Abbas et al., 2024	Requirement	“Content validity is mandatory for the validation of VR training tools.”	Evidence & credibility	Validation requirement	Table 3, Educational evaluation methodology
Abbas et al., 2024	Requirement	“Knowledge gained from using the VR simulation.”	Evidence & credibility	Outcome measure	Table 3, Research outcome measures
Abbas et al., 2024	Requirement	“Overall system usability.”	Usability	Outcome measure	Table 3, Research outcome measures
Abbas et al., 2024	Requirement	“Skill performance in real life situation at least 3 months after training.”	Evidence & credibility	Timepoint is explicit in table	Table 3, Research outcome measures
Abbas et al., 2024	Requirement	“Organisation outcomes such as adherence to standards, grouped patient outcomes, or litigation spend.”	Institutional support	Organisational outcomes	Table 3, Research outcome measures
Abbas et al., 2024	Requirement	“Reporting of adverse effects such as nausea, disorientation, or headaches which would indicate low safety or low levels of satisfaction as a result.”	Safety & comfort	Adverse effects	Table 3, Research outcome measures
Abbas et al., 2024	Barrier	“High cost.”	Resources & cost		Table 3, Integration of VR into medical curricula: barriers to technology adoption
Abbas et al., 2024	Barrier	“Lack of evidence of educational benefit.”	Evidence & credibility		Table 3, Integration of VR into medical curricula: barriers to technology adoption
Abbas et al., 2024	Barrier	“Digital readiness.”	Institutional support		Table 3, Integration of VR into medical curricula: barriers to technology adoption
Abbas et al., 2024	Barrier	“Lack of clarity of how a simulator is used within a curriculum.”	Integration in workflow		Table 3, Integration of VR into medical curricula: barriers to technology adoption
Abbas et al., 2024	Barrier	“Patient specific factors not taught.”	Relevance	Refers to content gaps	Table 3, Integration of VR into medical curricula: barriers to technology adoption
Abbas et al., 2024	Facilitator	“Easy access for your trainees or students to use the system.”	Usability		Table 3, Integration of VR into medical curricula: Design decisions
Abbas et al., 2024	Facilitator	“Low maintenance cost.”	Resources & cost		Table 3, Integration of VR into medical curricula: Design decisions
Abbas et al., 2024	Facilitator	“High level of graphical fidelity i. e., it looks more realistic.”	Relevance		Table 3, Integration of VR into medical curricula: Design decisions
Abbas et al., 2024	Facilitator	“The ability for your students to tolerate the experience for longer periods of time.”	Safety & comfort		Table 3, Integration of VR into medical curricula: Design decisions
Abbas et al., 2024	Facilitator	“Large volume of content or frequent updates with more content.”	Relevance		Table 3, Integration of VR into medical curricula: Design decisions
Abbas et al., 2024	Facilitator	“Low up-front cost of purchasing the system.”	Resources & cost		Table 3, Integration of VR into medical curricula: Design decisions
Abbas et al., 2024	Facilitator	“Low chance of technical issues.”	Usability		Table 3, Integration of VR into medical curricula: Design decisions
Alrashidi et al., 2025	Facilitator	“Time to use VR”	Integration in workflow	Time availability within routine practice	Results (Figure 2)
Alrashidi et al., 2025	Facilitator	“Technology support”	Resources & cost	Technical infrastructure and IT support	Results (Figure 2)
Alrashidi et al., 2025	Facilitator	“Patients motivation”	Patient acceptability	Wording preserved as in figure	Results (Figure 2)
Alrashidi et al., 2025	Facilitator	“Management support”	Institutional support	Organisational and managerial backing	Results (Figure 2)
Alrashidi et al., 2025	Facilitator	“Access to evidence”	Evidence & credibility	Availability of supporting evidence	Results (Figure 2)
Alrashidi et al., 2025	Facilitator	“Time to learn VR”	Training requirement	Time needed for learning VR	Results (Figure 2)
Alrashidi et al., 2025	Facilitator	“Access to educational opportunities about VR”	Training requirement	Formal education or learning opportunities	Results (Figure 2)
Alrashidi et al., 2025	Barrier	“Lack of interest”	Institutional support	Reflects professional or organisational engagement	Results (Figure 3)
Alrashidi et al., 2025	Barrier	“Lack of appropriate patients”	Relevance	Perceived mismatch with patient population	Results (Figure 3)
Alrashidi et al., 2025	Barrier	“Treatment space issues”	Integration in workflow	Physical space constraints	Results (Figure 3)
Alrashidi et al., 2025	Barrier	“Lack of evidence”	Evidence & credibility	Insufficient evidence base	Results (Figure 3)
Alrashidi et al., 2025	Barrier	“Poor match to patients needs/goals”	Relevance	Alignment with clinical goals	Results (Figure 3)
Alrashidi et al., 2025	Barrier	“Lack of funds”	Resources & cost	Financial limitations	Results (Figure 3)
Alrashidi et al., 2025	Barrier	“Lack of support to set-up VR equipment”	Resources & cost	Setup and deployment support	Results (Figure 3)
Alrashidi et al., 2025	Barrier	“Lack of support staff to administer VR”	Resources & cost	Staffing constraints	Results (Figure 3)

Chung et al., 2022	Facilitator	“break down barriers”	Patient acceptability	Quoted phrase within author narrative	Results, Patient Engagement
Chung et al., 2022	Facilitator	“could encourage earlier or sustained help-seeking”	Patient acceptability	Exact clause from author sentence	Results, Patient Engagement
Chung et al., 2022	Facilitator	“would have particular appeal for young people”	Patient acceptability	Exact author wording	Results, Patient Engagement
Chung et al., 2022	Barrier	“older people would be more reluctant to use VR”	Patient acceptability	Reported as mixed views	Results, Patient Engagement
Chung et al., 2022	Requirement	“evidence of its therapeutic efficacy”	Evidence & credibility	Author-defined requirement	Results, Therapeutic Efficacy
Chung et al., 2022	Barrier	“Most staff were unaware of the current VR evidence-base”	Evidence & credibility	Exact author sentence	Results, Therapeutic Efficacy
Chung et al., 2022	Barrier	“real enough”	Evidence & credibility	Quoted fragment embedded in narrative	Results, Therapeutic Efficacy
Chung et al., 2022	Barrier	“translate to the same kind of clinical results”	Evidence & credibility	Exact quoted fragment	Results, Therapeutic Efficacy
Chung et al., 2022	Requirement	“a clear evidence base, or some evidence”	Evidence & credibility	Exact quotation	Results, Therapeutic Efficacy
Chung et al., 2022	Requirement	“ongoing evaluation”	Evidence & credibility	Appears within quoted sentence	Results, Therapeutic Efficacy
Chung et al., 2022	Facilitator	“practical”	Integration in workflow	Quoted adjective	Results, Clinical Applications
Chung et al., 2022	Facilitator	“exposure work”	Relevance	Quoted clinical term	Results, Clinical Applications
Chung et al., 2022	Facilitator	“minimising risk is fantastic”	Safety & comfort	Full participant quote	Results, Clinical Applications
Chung et al., 2022	Barrier	“I’d be concerned of how it would impact on their reality testing”	Safety & comfort	Full participant quote	Results, Clinical Applications
Chung et al., 2022	Barrier	“Not in the current format we run our groups”	Integration in workflow	Full participant quote	Results, Clinical Applications
Chung et al., 2022	Facilitator	“people may not necessarily need to go to see the psychiatrist for early parts”	Integration in workflow	Stepped-care implication	Results, Clinical Applications
Chung et al., 2022	Barrier	“too distressed”	Safety & comfort	Quoted descriptor	Results, Safety and Ethical Concerns
Chung et al., 2022	Barrier	“feeling out of body, unreal”	Safety & comfort	Quoted descriptors	Results, Safety and Ethical Concerns
Chung et al., 2022	Barrier	“touching”	Legal & ethical alignment	Ethical concern expressed verbatim	Results, Safety and Ethical Concerns
Chung et al., 2022	Requirement	“specific protocols would need to be developed”	Legal & ethical alignment	Exact author wording	Results, Safety and Ethical Concerns
Chung et al., 2022	Requirement	“clear financial viability”	Resources & cost	Author wording	Results, Business Case
Chung et al., 2022	Requirement	“collaborative planning”	Institutional support	Exact phrase from narrative	Results, Collaborative Stakeholder Planning
Chung et al., 2022	Facilitator	“trialling”	Institutional support	Quoted cultural descriptor	Results, Service Culture
Chung et al., 2022	Barrier	“resourcing challenges”	Resources & cost	Subtheme label, appears verbatim	Table 2
Chung et al., 2022	Requirement	“education and training”	Training requirement	Subtheme label	Table 2
Chung et al., 2022	Barrier	“bulky”	Usability	Quoted adjective	Results, VR System Usability
Chung et al., 2022	Barrier	“heavy”	Usability	Quoted adjective	Results, VR System Usability
Chung et al., 2022	Requirement	“increased customisability”	Usability	Exact author wording	Results, VR System Usability
Chung et al., 2022	Requirement	“collaborative stakeholder planning”	Institutional support		Table 2
Chung et al., 2022	Requirement	“local opinion leaders to champion change”	Institutional support		Figure 3
Chung et al., 2022	Facilitator	“a service culture supportive of innovation”	Institutional support		Figure 3
Chung et al., 2022	Barrier	“resourcing challenges”	Resources & cost		Figure 3
Chung et al., 2022	Requirement	“education and training”	Training requirement		Figure 3
Chung et al., 2022	Requirement	“training in technical VR skills”	Training requirement		Results, Education and Training
Chung et al., 2022	Requirement	“assessing patient suitability”	Training requirement		Results, Education and Training
Chung et al., 2022	Requirement	“managing ethical and safety risks”	Training requirement		Results, Education and Training
Chung et al., 2022	Barrier	“fear of change and resistance to new therapeutic approaches”	Institutional support		Results, Staff Attitudes Towards Technology
Chung et al., 2022	Barrier	“VR system usability” concerns including “bulky” and “heavy” headsets	Usability	Quoted adjectives used in Results	Results, VR System Usability
Chung et al., 2022	Requirement	“increased customisability”	Usability		Results, VR System Usability
Chung et al., 2023	Mixed	“participants also expressed conflicting beliefs regarding VR’s appropriateness for people with psychosis, dementia, and learning disabilities.”	Relevance		Results – Beliefs About Consequences
Chung et al., 2023	Barrier	“numerous concerns were raised, including beliefs VR would be too distressing for some patients (e.g., severe PTSD) and could cause known/unknown adverse effects (e.g., sickness, physical injury, medication interaction, encourage risk-taking behaviour).”	Safety & comfort		Results – Beliefs About Consequences

Chung et al., 2023	Mixed	“views varied regarding VR’s likely appeal vs. aversiveness to patients and the influence of patient age (e.g., greater appeal among young people; reluctance from older adults).”	Patient acceptability		Results – Beliefs About Consequences
Chung et al., 2023	Facilitator	“VR was commonly discussed in the context of ‘adjunctive use’ to current therapies, and its introduction seen as conferring ‘organisational benefits’, including increased service capacity and positive brand impact.”	Institutional support	Clinical and organisational implications combined	Results – Beliefs About Consequences
Chung et al., 2023	Barrier	“a minority of participants expressed concerns of VR presenting a ‘threat to practice’, including replacing clinicians and diminishing the credibility of therapeutic programs.”	Institutional support		Results – Beliefs About Consequences
Chung et al., 2023	Mixed	“Some participants also discussed VR’s ‘therapeutic efficacy’, including its effectiveness compared to current treatments, and its impact on therapeutic alliance.”	Evidence & credibility		Results – Beliefs About Consequences
Chung et al., 2023	Barrier	“beliefs conflicted regarding its user-friendliness (e.g., bulkiness, heavy weight, transportability), and concerns were expressed about the limited range of empirically validated scenarios.”	Usability		Results – Environmental Context and Resources
Chung et al., 2023	Mixed	“The ‘financial viability’ of VR (e. g., cost-effectiveness, insurance coverage) and ‘treatment space’ requirements (e.g., need for dedicated space when rooms in health services are limited) were also commonly discussed.”	Resources & cost	Financial and spatial resources combined	Results – Environmental Context and Resources
Chung et al., 2023	Barrier	“its feasibility was questioned as its use was perceived to require an individualised, tailored approach that current structures (e.g., predominant group therapy model, ward routines) could not support.”	Integration in workflow		Results – Environmental Context and Resources
Chung et al., 2023	Barrier	“one manager cautioned against introducing VR during periods of active or recent change, as change on multiple fronts could overwhelm staff and lead to suboptimal implementation.”	Institutional support		Results – Environmental Context and Resources
Chung et al., 2023	Requirement	“Participants also discussed ‘promotion of therapeutic VR’ as a treatment option and the need for external ‘clinical regulation’ of VR applications to ensure safe and ethical usage.”	Legal & ethical alignment	Promotion and regulation combined	Results – Environmental Context and Resources
Chung et al., 2023	Barrier	“Participants reported limited ‘general knowledge’ about VR, with most only aware of VR’s use in gaming and entertainment, and some reporting no VR knowledge at all.”	Training requirement		Results – Knowledge
Chung et al., 2023	Requirement	“Thus, staff identified a need for education (i.e., use cases, benefits) and to increase awareness among referring clinicians and consumers about VR as a treatment option.”	Training requirement		Results – Knowledge
Chung et al., 2023	Requirement	“Staff also identified gaps related to ‘procedural knowledge’, including how to select appropriate clients for VR, applying VR clinically, and having awareness of safety risks and their management procedures.”	Training requirement		Results – Knowledge
Chung et al., 2023	Barrier	“Additionally, staff highlighted the need to address ‘evidence gaps’ related to the strength of research evidence and patient acceptability of VR therapies.”	Evidence & credibility		Results – Knowledge
Chung et al., 2023	Requirement	“Concerns about ‘professional ethics’ in relation to maintaining appropriate boundaries around physical touch to prevent patient injury were also raised.”	Legal & ethical alignment		Results – Social and Professional Role and Identity

Chung et al., 2023	Barrier	“Negative emotions tended to be more common among staff who were unfamiliar with VR, which often subsided with experience (e.g., anxiety about bumping into walls abating once feeling comfortable with the chaperone function).”	Safety & comfort	Staff safety perception	Results – Emotions
Chung et al., 2023	Requirement	“Several participants discussed the importance of VR being delivered by skilful clinicians experienced with the clinical conditions a VR system has been developed for, to minimise the likelihood of overstimulation and inadvertent psychological harm (e.g., distress after exposure to traumatic memories).”	Training requirement		Results – Skills
Chung et al., 2023	Facilitator	“At the service level, participants identified factors that would support therapeutic VR ‘implementation decision-making’, including existing organisational processes and committees, and evidence of robust therapeutic efficacy.”	Institutional support		Results – Memory, Attention, and Decision Processes
Chung et al., 2023	Requirement	“at the individual provider level, staff identified a need for clinical guidelines to facilitate ‘clinical decision-making’, including selection of appropriate patients for, and delivery of therapeutic VR in a safe, efficacious manner.”	Integration in workflow		Results – Memory, Attention, and Decision Processes
Deason et al., 2025	Requirement	“voice commanded, which allows hands-free operation, an essential feature for wound care scenarios that often require both hands for patient management”	Usability		Case Study: Results From the Île-à-la-Crosse Pilot
Deason et al., 2025	Requirement	“its ability to connect to both Wi-Fi and cellular networks provides the flexibility needed to operate across multiple clinical settings, including the potential to be taken directly into patients’ homes where connectivity infrastructure may vary”	Resources & cost	Connectivity framed as infrastructure feasibility	Case Study: Results From the Île-à-la-Crosse Pilot
Deason et al., 2025	Facilitator	“The TeleVU Glass View’s portability and compatibility with existing infrastructure in Île-à-la-Crosse made it a practical choice for implementation”	Integration in workflow		Case Study: Results From the Île-à-la-Crosse Pilot
Deason et al., 2025	Facilitator	“These consultations have allowed patients to access specialized wound care support in their home community of Île-à-la-Crosse without needing to travel to distant tertiary centers for specialist care”	Resources & cost	Reduced travel burden	Implementation Plan
Deason et al., 2025	Barrier	“One common challenge is the establishment of criteria weights, which determine the relative importance of different criteria and quantify the benefit of the technology”	Evidence & credibility		Discussion
Deason et al., 2025	Requirement	“criteria should be nonoverlapping to prevent double-counting of their value domains, as well as preferentially independent, meaning the performance of one variable should not affect the weight of another”	Evidence & credibility	Methodological requirement	Discussion
Deason et al., 2025	Barrier	“Tailored and relevant information can be resource intensive and costly to obtain, restricting the applicability of the assessment’s conclusions”	Resources & cost		Discussion
Deason et al., 2025	Requirement	“organizations are encouraged to convene a multidisciplinary panel that includes clinical experts, technical specialists, and implementation leads to define local priorities and customize the scoring framework accordingly”	Institutional support		Discussion
Deason et al., 2025	Requirement	“contextual variables such as infrastructure readiness and workforce capacity should be integrated into the scoring process to enhance relevance”	Institutional support		Discussion

Deason et al., 2025	Requirement	“patient reported outcomes and satisfaction scores could be weighted alongside technological attribute quality and clinical relevance to ensure that the selected technologies align with patient expectations and experiences”	Patient acceptability		Discussion
Deason et al., 2025	Requirement	“economic impact, equity of access, and societal/organizational considerations, can also be added to the weighting system”	Resources & cost	Multiple domains mentioned, primary framing economic	Discussion
Deason et al., 2025	Requirement	“criteria related to power supply requirements, internet connectivity standards, or compatibility with existing electronic medical record systems could be added to ensure that selected technologies are feasible to deploy in diverse health care environments”	Resources & cost	EMR compatibility noted	Discussion
Deason et al., 2025	Requirement	“In remote or resource-limited settings, criteria such as the ability to function on low-bandwidth networks, portability for transport between locations, and durability in harsh environmental conditions may be critical to ensuring successful implementation”	Resources & cost	Portability overlaps with workflow, kept here due to infrastructure framing	Discussion
Deason et al., 2025	Barrier	“The small number of technologies initially included in the TST’s evaluation pool limited the breadth of comparison”	Evidence & credibility		Discussion
Deason et al., 2025	Barrier	“variability in clinical relevance ratings among individual clinicians”	Evidence & credibility		Discussion
Deason et al., 2025	Barrier	“evidence supporting the tool’s application is limited in scope and does not reflect the broader dimensions typically considered in HTA frameworks”	Evidence & credibility		Discussion
Deason et al., 2025	Requirement	“expanding the pilot to include additional technologies and clinical needs”	Evidence & credibility	Validation requirement	Discussion
Deason et al., 2025	Requirement	“expanded collaborations with health organizations, government departments, and policymakers will be essential to support the broader adoption of the TST and the technologies it recommends”	Institutional support		Discussion
Deason et al., 2025	Requirement	“Standardizing this process through more robust training and consensus-building exercises could enhance the reliability of the ratings.”	Training requirement	Addresses clinician rating variability and reliability	Discussion
Deason et al., 2025	Requirement	“The integration of feedback mechanisms into the TST’s algorithm will also be needed to ensure continuous improvement and alignment with emerging clinical and technological advancements.”	Evidence & credibility	Requirement for ongoing validation and improvement	Discussion
Elser et al., 2024	Barrier	“A negative factor was that the VR glasses were heavy to wear”	Safety & comfort		Results
Elser et al., 2024	Barrier	“Yeah, that would really have to be under guidance, yeah. […] So they [peers] wouldn’t be able to do it alone”	Institutional support		Results
Elser et al., 2024	Barrier	“I’ve got really bad arthritis too at the moment so holding onto those [hand controls] was an issue”	Usability		Results
Elser et al., 2024	Barrier	“I had some trouble figuring out which controls to use to move around so um I’ve never played computer games before and maybe that had something to do with it. I felt like a total idiot totally frustrated and not able to catch onto what to do”	Training requirement	Could also map to usability	Results
Elser et al., 2024	Barrier	“The exceptions were those whose first language was not English and who described difficulties in understanding game instructions”	Usability		Results
Elser et al., 2024	Facilitator	“I had really high hopes...I thought it might actually take my pain away”	Patient acceptability		Results

Elser et al., 2024	Facilitator	“I’ve taken opiates for 40 years and they don’t work as well as what the virtual reality did”	Evidence & credibility	Results
Elser et al., 2024	Facilitator	“Especially in the future, you could have thousands of different situations that you could immerse yourself in, for as much time as you want in the day”	Relevance	Results
Elser et al., 2024	Facilitator	“I’m not in as much pain when I’ m seated as when I’m standing, so it was quite easy for me to do the movements”	Safety & comfort	Results
Elser et al., 2024	Facilitator	“I think it makes you feel better that it’s a trained physiotherapist. You knew they had that background and it just fills you with confi dence a bit more”	Training requirement	Results
Elser et al., 2024	Facilitator	“Positive factors were that VR added a dimension of playfulness and gaming to the exercise”	Patient acceptability	Results
Elser et al., 2024	Facilitator	“The competition against the computer opponents increased engagement and several participants mentioned the feeling of satisfaction they got when they performed well”	Patient acceptability	Results
Elser et al., 2024	Facilitator	“you’re enjoying yourself, you can do things you’ve never experienced before, obviously you’re going to do it”	Patient acceptability	Results
Elser et al., 2024	Facilitator	“Health care professionals indicated that the opportunity to be with the patient during the VR intervention and to be able to intervene in adverse events supports its implementation.”	Safety & comfort	Results
Elser et al., 2024	Facilitator	“Another facilitating factor from the health care professional perspective was that the VR intervention allows patients to practice everyday situations in therapy, such as working in the garden.”	Relevance	Results
Elser et al., 2024	Requirement	“an important step in implementing VR interventions appears to be the selection of an appropriate VR device and VR software for patients with chronic pain and in consideration of their actual conditions.”	Safety & comfort	Discussion
Elser et al., 2024	Requirement	“Both can be addressed in software development, such as by participatory developed tutorials or using plain language.”	Usability	Discussion
Elser et al., 2024	Requirement	“our findings emphasize the importance of developing and providing plain-language options in VR interventions for people with chronic pain”	Usability	Discussion
Elser et al., 2024	Requirement	“As part of such a strategy, special attention should be given to competencies of health care professionals related to the use of VR to enable them to teach the acquired skills to their patients with individual needs”	Training requirement	Discussion
Elser et al., 2024	Requirement	“Additionally, for a successful implementation, it is important that health care professionals are positive about the digital technology”	Institutional support	Discussion
Elser et al., 2024	Requirement	“and perceive it as user-friendly”	Usability	Discussion
Elser et al., 2024	Requirement	“Thus, a key aspect of implementing VR in the treatment of chronic pain is adequate training of the health care professionals who will provide the VR interventions to people with chronic pain.”	Training requirement	Discussion
Elser et al., 2024	Requirement	“When implementing VR interventions for people with chronic pain, this positive belief can be used and facilitated by educating patients about the positive effects of the intervention and presenting best-practice examples.”	Evidence & credibility	Discussion

Felnhofer et al., 2025	Facilitator	“expanded therapeutic options, such as improved access to therapy, tailored environments, flexible exposure settings, and immediate stimulus generation”	Relevance		Results – Characteristics of VR users
Felnhofer et al., 2025	Facilitator	“VR was seen as secure, motivating, offering novel, engaging experiences.”	Patient acceptability	Multiple advantages in one sentence	Results – Characteristics of VR users
Felnhofer et al., 2025	Barrier	“high technical demands”	Usability		Results – Characteristics of VR users
Felnhofer et al., 2025	Barrier	“additional preparation time”	Integration in workflow		Results – Characteristics of VR users
Felnhofer et al., 2025	Barrier	“underdeveloped technology”	Usability		Results – Characteristics of VR users
Felnhofer et al., 2025	Barrier	“image glitches or device failures”	Usability		Results – Characteristics of VR users
Felnhofer et al., 2025	Barrier	“lack of provider support”	Institutional support		Results – Characteristics of VR users
Felnhofer et al., 2025	Barrier	“cybersickness”	Safety & comfort	Author-defined label	Results – Characteristics of VR users
Felnhofer et al., 2025	Barrier	“the risk of patients avoiding exposure (i.e., by closing their eyes) without the therapist’s awareness.”	Integration in workflow	Spacing normalized only	Results – Characteristics of VR users
Felnhofer et al., 2025	Requirement	“a need for more research and development”	Evidence & credibility	Spacing normalized only	Results – Characteristics of VR users
Felnhofer et al., 2025	Requirement	“user-friendly programs approved by health insurance”	Institutional support		Results – Characteristics of VR users
Felnhofer et al., 2025	Requirement	“better-quality software”	Usability		Results – Characteristics of VR users
Felnhofer et al., 2025	Barrier	“I have reservations about VR so far.”	Training requirement		Results – Table 3
Felnhofer et al., 2025	Barrier	“I am not sure for which patients VR is beneficial.”	Relevance		Results – Table 3
Felnhofer et al., 2025	Barrier	“I’m not sure about the evidence for VR treatments.”	Evidence & credibility		Results – Table 3
Felnhofer et al., 2025	Barrier	“There are barriers that make it impossible for me to use it.”	Integration in workflow	Non-specific, retained verbatim	Results – Table 3
Felnhofer et al., 2025	Barrier	“No relevance or not applicable for my patients.”	Relevance		Results – Table 3
Felnhofer et al., 2025	Barrier	“Not interested.”	Training requirement		Results – Table 3
Felnhofer et al., 2025	Barrier	“I don’t see any advantages for me or my patients.”	Relevance		Results – Table 3
Felnhofer et al., 2025	Barrier	“lack of or limited knowledge”	Training requirement	Author-defined sub-theme label	Results – Professional factors
Felnhofer et al., 2025	Barrier	“lack of training”	Training requirement	Author-defined sub-theme label	Results – Professional factors
Felnhofer et al., 2025	Barrier	“personal reasons”	Training requirement	Author-defined sub-theme label	Results – Professional factors
Felnhofer et al., 2025	Barrier	“lack of time”	Integration in workflow	Author-defined sub-theme label	Results – Professional factors
Felnhofer et al., 2025	Barrier	“little to no knowledge of VR technology”	Training requirement		Results – Lack of or limited knowledge
Felnhofer et al., 2025	Barrier	“no prior contact with it”	Training requirement		Results – Lack of or limited knowledge
Felnhofer et al., 2025	Barrier	“being uninformed about relevant scientific evidence.”	Evidence & credibility		Results – Lack of or limited knowledge
Felnhofer et al., 2025	Barrier	“entirely unfamiliar with the technology, unaware of its existence or even its use in psychotherapy.”	Training requirement		Results – Lack of or limited knowledge
Felnhofer et al., 2025	Barrier	“lack of or little direct experience with VR”	Training requirement		Results – Lack of or limited knowledge
Felnhofer et al., 2025	Barrier	“No knowledge and no self-experience in a therapeutic setting.”	Training requirement	Participant quote	Results – Lack of or limited knowledge
Felnhofer et al., 2025	Barrier	“perceived lack of VR specific training and further education.”	Training requirement		Results – Lack of training
Felnhofer et al., 2025	Barrier	“No training on this, and too little knowledge of what I can do with it and how.”	Training requirement	Spacing normalized only	Results – Lack of training
Felnhofer et al., 2025	Barrier	“vision impairments”	Safety & comfort	Author-defined factor	Results – Personal reasons
Felnhofer et al., 2025	Barrier	“age-related concerns (i.e., feeling too old or approaching retirement)”	Training requirement		Results – Personal reasons
Felnhofer et al., 2025	Barrier	“a perceived lack of technological skills.”	Training requirement		Results – Personal reasons
Felnhofer et al., 2025	Barrier	“engaging with VR was too cumbersome.”	Usability	Spacing normalized only	Results – Personal reasons
Felnhofer et al., 2025	Barrier	“high acquisition costs”	Resources & cost		Results – Costs
Felnhofer et al., 2025	Barrier	“high running costs”	Resources & cost		Results – Costs
Felnhofer et al., 2025	Barrier	“a lack of suitable facilities (i.e., rooms)”	Resources & cost		Results – Costs
Felnhofer et al., 2025	Barrier	“insufficient funding from external sources”	Resources & cost		Results – Costs
Felnhofer et al., 2025	Barrier	“cost-benefit ratio”	Resources & cost	Author-defined sub-theme label	Results – Financial factors
Felnhofer et al., 2025	Barrier	“clinical applicability”	Relevance	Author-defined sub-theme label	Results – Therapeutic factors
Felnhofer et al., 2025	Barrier	“concerns about the ‘real’ therapeutic relationship”	Patient acceptability	Author-defined sub-theme label	Results – Therapeutic factors

Felnhofer et al., 2025	Barrier	“immature technology”	Usability	Author-defined sub-theme label	Results – Technological factors
Felnhofer et al., 2025	Barrier	“lack of equipment”	Resources & cost	Author-defined sub-theme label	Results – Technological factors
Felnhofer et al., 2025	Barrier	“Triggers dizziness, nausea, vomiting and headaches for me.”	Safety & comfort		Results – Cybersickness
Felnhofer et al., 2025	Barrier	“Kinetic nausea in about 50% of users.”	Safety & comfort		Results – Cybersickness
Felnhofer et al., 2025	Barrier	“lack of reimbursement for VR-based therapies”	Institutional support		Discussion – Opportunity
Felnhofer et al., 2025	Barrier	“inadequate workplace support for implementing the technology.”	Institutional support		Discussion – Opportunity
Felnhofer et al., 2025	Requirement	“introducing comprehensive education programs as early as in university curricula”	Training requirement		Discussion – Capability
Felnhofer et al., 2025	Requirement	“Reinforcing clinical guidelines and offering specialized training to build practical skills”	Training requirement		Discussion – Capability
Felnhofer et al., 2025	Requirement	“developing specified clinical guidelines for therapeutic VR usage.”	Institutional support		Conclusion
Felnhofer et al., 2025	Requirement	“online courses and introductory workshops covering the basics of VR technology”	Training requirement		Conclusion
Felnhofer et al., 2025	Requirement	“hands-on training with immersive demonstrations and supervised practice sessions with case studies”	Training requirement		Conclusion
Felnhofer et al., 2025	Requirement	“mentorship programs pairing VR novices with experienced clinicians”	Training requirement		Conclusion
Felnhofer et al., 2025	Requirement	“online forums for sharing experiences”	Institutional support		Conclusion
Felnhofer et al., 2025	Requirement	“introducing reimbursements in the public health sector for VR treatments”	Institutional support		Conclusion
Felnhofer et al., 2025	Requirement	“supporting the development and implementation of specialized VR software beyond exposure therapy”	Relevance		Conclusion
Glegg & Levac, 2018	Determinant	Knowledge about the differences between VR and conventional therapy (including benefits)	Evidence & credibility		Results – Table 2
Glegg & Levac, 2018	Determinant	Knowledge of disadvantages of different systems	Evidence & credibility		Results – Table 2
Glegg & Levac, 2018	Determinant	Awareness of usefulness of the technology	Evidence & credibility		Results – Table 2
Glegg & Levac, 2018	Determinant	Knowledge to operate the technology	Training requirement		Results – Table 2
Glegg & Levac, 2018	Determinant	Familiarity with games/tasks	Training requirement		Results – Table 2
Glegg & Levac, 2018	Determinant	Knowledge to apply the technology clinically	Training requirement		Results – Table 2
Glegg & Levac, 2018	Determinant	Familiarity of clients with technology	Patient acceptability		Results – Table 2
Glegg & Levac, 2018	Determinant	Therapist experience with the system	Training requirement		Results – Table 2
Glegg & Levac, 2018	Determinant	Client experience with the system	Patient acceptability		Results – Table 2
Glegg & Levac, 2018	Determinant	Skills to optimize patient outcomes	Training requirement		Results – Table 2
Glegg & Levac, 2018	Determinant	Attitude of therapist toward the technology	Institutional support		Results – Table 2
Glegg & Levac, 2018	Determinant	Attitude of patient toward the technology	Patient acceptability		Results – Table 2
Glegg & Levac, 2018	Determinant	Reconciling therapist role in gaming context	Institutional support		Results – Table 2
Glegg & Levac, 2018	Determinant	Being tech-savvy (clients and therapists)	Training requirement		Results – Table 2
Glegg & Levac, 2018	Determinant	Compatibility with treatment preferences/needs	Relevance		Results – Table 2
Glegg & Levac, 2018	Determinant	Keeping up with rapid technological advances	Training requirement		Results – Table 2
Glegg & Levac, 2018	Determinant	Self-perceptions of being generally tech-savvy	Training requirement		Results – Table 2
Glegg & Levac, 2018	Determinant	Availability of education opportunities	Institutional support		Results – Table 2
Glegg & Levac, 2018	Determinant	Perceived ease of use of the technology (for both clinicians and patients)	Usability		Results – Table 2
Glegg & Levac, 2018	Determinant	Self-efficacy in clinical use of the technology	Training requirement		Results – Table 2
Glegg & Levac, 2018	Determinant	Skepticism about its benefits	Evidence & credibility		Results – Table 2

Glegg & Levac, 2018	Determinant	Perceived therapeutic benefit to clients	Relevance	Results – Table 2
Glegg & Levac, 2018	Determinant	Perceived match to client's goals/needs	Relevance	Results – Table 2
Glegg & Levac, 2018	Determinant	Perceived match to therapist's needs	Institutional support	Results – Table 2
Glegg & Levac, 2018	Determinant	Perceived motivational utility	Patient acceptability	Results – Table 2
Glegg & Levac, 2018	Determinant	Concerns about screen time	Safety & comfort	Results – Table 2
Glegg & Levac, 2018	Determinant	Transfer of training to real life	Relevance	Results – Table 2
Glegg & Levac, 2018	Determinant	Privacy concerns of social media use/confidentiality of data	Legal & ethical alignment	Results – Table 2
Glegg & Levac, 2018	Determinant	Potential for physical fatigue	Safety & comfort	Results – Table 2
Glegg & Levac, 2018	Determinant	Confidence in expert clinician/mentor	Institutional support	Results – Table 2
Glegg & Levac, 2018	Determinant	Ethical issues	Legal & ethical alignment	Results – Table 2
Glegg & Levac, 2018	Determinant	Legal issues	Legal & ethical alignment	Results – Table 2
Glegg & Levac, 2018	Determinant	Relative advantage over other options	Relevance	Results – Table 2
Glegg & Levac, 2018	Determinant	Technical issues	Usability	Results – Table 2
Glegg & Levac, 2018	Determinant	Treatment space issues (eg, dedicated; proximity to typical treatment areas, getting patients to room)	Integration in workflow	Results – Table 2
Glegg & Levac, 2018	Determinant	Organizational support	Institutional support	Results – Table 2
Glegg & Levac, 2018	Determinant	Opportunity for client self-practice	Integration in workflow	Results – Table 2
Glegg & Levac, 2018	Determinant	Opportunity for additional practice outside of regular therapy sessions	Integration in workflow	Results – Table 2
Glegg & Levac, 2018	Determinant	Opportunity for social interaction	Patient acceptability	Results – Table 2
Glegg & Levac, 2018	Determinant	Opportunity for competition	Patient acceptability	Results – Table 2
Glegg & Levac, 2018	Determinant	Degree of system/game's match to client's needs/goals	Relevance	Results – Table 2
Glegg & Levac, 2018	Determinant	Technical issues	Usability	Results – Table 2
Glegg & Levac, 2018	Determinant	Treatment space issues (eg, dedicated; proximity to typical treatment areas, getting patients to room)	Integration in workflow	Results – Table 2
Glegg & Levac, 2018	Determinant	Organizational support	Institutional support	Results – Table 2
Glegg et al., 2018	Requirement	high-quality evidence demonstrating that VR/AVG is at least equal to, if not more effective than, other treatment options	Evidence & credibility	Research Gaps
Glegg et al., 2018	Requirement	guidance with respect to optimal dosage, key therapeutic ingredients, and information about safety and contraindications	Evidence & credibility	Research Gaps
Glegg et al., 2018	Requirement	outcomes at the activity and participation levels	Evidence & credibility	Research Gaps
Glegg et al., 2018	Requirement	to what extent skills learned in a virtual environment transfer to real-life functional settings	Relevance	Research Gaps
Glegg et al., 2018	Barrier	Population-specific treatment guidelines do not exist in this field	Evidence & credibility	Research Gaps
Glegg et al., 2018	Requirement	Identifying methods to evaluate client progress, and offering guidance in the interpretation of system-integrated assessment features	Usability	Research Gaps
Glegg et al., 2018	Requirement	High-quality evidence demonstrating that VR/AVG is at least equal to, if not more effective than, other treatment options	Evidence & credibility	Research Gaps
Glegg et al., 2018	Requirement	Optimal dosage, key therapeutic ingredients, and information about safety and contraindications	Evidence & credibility	Research Gaps
Glegg et al., 2018	Requirement	Outcomes at the activity and participation levels	Evidence & credibility	Research Gaps
Glegg et al., 2018	Requirement	To what extent skills learned in a virtual environment transfer to real-life functional settings	Relevance	Research Gaps
Glegg et al., 2018	Barrier	Population-specific treatment guidelines do not exist in this field	Evidence & credibility	Research Gaps
Glegg et al., 2018	Requirement	Identifying methods to evaluate client progress, and offering guidance in the interpretation of system-integrated assessment features	Usability	Research Gaps

Glegg et al., 2018	Requirement	a balance between adequate flexibility in the design of customizable software parameters while avoiding overwhelming clinicians with abundant decision-making requirements	Usability		Technology Development
Glegg et al., 2018	Barrier	Motivation for clients to participate in VR/AVG often declines over time as the novelty fades	Patient acceptability		Technology Development
Glegg et al., 2018	Requirement	Involving therapists in decision making about which systems will best meet their needs	Institutional support		Facilitated Implementation
Glegg et al., 2018	Requirement	context-specific barrier assessments for any clinical site in which VR/AVG implementation is being planned	Institutional support		Facilitated Implementation
Kouijzer et al., 2023	Barrier	A decline in cognitive capabilities, such as reasoning and problem-solving, could negatively affect VR use	Relevance		Table 2 Barriers to implementation
Kouijzer et al., 2023	Barrier	Motion- or cybersickness experienced while using VR	Safety & comfort		Table 2 Barriers to implementation
Kouijzer et al., 2023	Barrier	Lack of data privacy and security when using patient data in VR	Legal & ethical alignment		Table 2 Barriers to implementation
Kouijzer et al., 2023	Barrier	Issues with the usability and user-friendliness of VR	Usability		Table 2 Barriers to implementation
Kouijzer et al., 2023	Barrier	The use of VR adds additional steps for healthcare providers during treatment	Integration in workflow		Table 2 Barriers to implementation
Kouijzer et al., 2023	Barrier	Costs of purchasing and time for maintaining VR	Resources & cost		Table 2 Barriers to implementation
Kouijzer et al., 2023	Barrier	Perceived lack of research and evidence on the added value of VR	Evidence & credibility		Table 2 Barriers to implementation
Kouijzer et al., 2023	Barrier	Perceived lack of support from management in using VR	Institutional support		Table 2 Barriers to implementation
Kouijzer et al., 2023	Barrier	Lack of perceived time and opportunities to learn how to use VR and integrate VR in treatment	Training requirement		Table 2 Barriers to implementation
Kouijzer et al., 2023	Facilitator	Availability of validated evidence on the value of VR for treatment	Evidence & credibility		Table 3 Facilitators to implementation
Kouijzer et al., 2023	Facilitator	Client is physically safe in treatment room while using VR hardware	Safety & comfort		Table 3 Facilitators to implementation
Kouijzer et al., 2023	Facilitator	The VR hardware and software is easy to use by end-users	Usability		Table 3 Facilitators to implementation
Kouijzer et al., 2023	Facilitator	The capacity to combine and integrate VR in existing treatment	Integration in workflow		Table 3 Facilitators to implementation
Kouijzer et al., 2023	Facilitator	Perceived support from management to use VR	Institutional support		Table 3 Facilitators to implementation
Kouijzer et al., 2023	Requirement	Availability of treatment manuals and protocols on how to use VR in practice	Integration in workflow		Table 4 Summary of implementation strategies mentioned in included records
Kouijzer et al., 2023	Requirement	Hiring support staff to use VR, to coach clinicians in the use of VR, or to maintain the VR system	Resources & cost		Table 4 Summary of implementation strategies mentioned in included records
Kouijzer et al., 2023	Requirement	Training (in person and online) to learn how to use VR and discussing appropriate content in treatment	Training requirement		Table 4 Summary of implementation strategies mentioned in included records
Kouijzer et al., 2023	Requirement	Use a theoretical framework to guide development of relevant implementation strategies to enhance uptake	Institutional support	Could also fit Evidence & credibility, kept here as governance support	Table 5 Recommendations on implementation and the number of publications they were mentioned in (n)
Kouijzer et al., 2023	Barrier	a lack of confidence and self-efficacy in healthcare providers to work with VR during treatment.	Training requirement		Results, Barriers to implementation
Kouijzer et al., 2023	Barrier	the limited space of the treatment room that limits freedom of movement	Safety & comfort		Results, Barriers to implementation
Kouijzer et al., 2023	Barrier	the feeling of isolation while wearing the headset	Patient acceptability	Near-duplicate of Table 2 phrasing, "The VR headset can isolate patients from human contact." Kept here because this wording appears in the Results narrative.	Results, Barriers to implementation

Kouijzer et al., 2023	Barrier	The explicit conceptualization of implementation outcomes and the use of these outcomes to formulate implementation objectives or design implementation strategies was not described as such in the included records.	Evidence & credibility	Determinant is framed as an absence in the literature included in the scoping review.	Results, Implementation strategies, objectives, and outcomes
Kouijzer et al., 2023	Barrier	they were not integrated as outcomes into a systematic implementation process.	Institutional support	Refers to "acceptability, adoption, uptake, or feasibility", and is closely related to the prior row, kept as separate verbatim sentence.	Results, Implementation strategies, objectives, and outcomes
Kouijzer et al., 2024	Facilitator	“Their first experience was regarded as fun, increasing curiosity, creating possibilities, and overall experiencing feelings of enjoyment.”	Patient acceptability		3.1.1 Positive first impressions
Kouijzer et al., 2024	Facilitator	“I did not know what to expect. I never experienced it before, but I was curious, and it was awesome. (…) It’s good to practice in VR how to find peace again. I would highly recommend this.” (P8)	Patient acceptability		3.1.1 Positive first impressions
Kouijzer et al., 2024	Facilitator	“Explaining experiences [face-to-face] is more difficult than showing it [in VR].” (P5)	Relevance		3.1.1 Positive first impressions
Kouijzer et al., 2024	Facilitator	“Physically you are in the [treatment] room, but mentally you are in VR. It’s a strange feeling. It really can be compared to a game” (P3).	Patient acceptability	Presence framed as engaging	3.1.1 Positive first impressions
Kouijzer et al., 2024	Barrier	“I do not experience it as real; it feels unrealistic. There is still very little feeling or emotion [while conversing with a virtual character]. So, I cannot see if he means what he says. It is now very fake and superficial.” (P7).	Usability		3.1.2 Points of improvement
Kouijzer et al., 2024	Barrier	“Now the person facing you is still robotic. It must seem somewhat real to use for people with aggression problems. The movements and appearance feel very unnatural.” (P2).	Usability		3.1.2 Points of improvement
Kouijzer et al., 2024	Requirement	“It would be nice if you could also look in the store through the window or hear some more background noises. Maybe you can walk everywhere and hear sounds from the houses above or birds flying and chirping above you.” (P6)	Usability	Feature request framed by patient	3.1.2 Points of improvement
Kouijzer et al., 2024	Barrier	“It’s different. You stand still yourself [in real-life], but it feels like I’m walking [in VR]. Everything I see has to be processed by my brain. So basically, my brain is being fooled and that’s why it feels so weird in my body. The reality doesn’t match and that makes me dizzy for a while.” (P7)	Safety & comfort		3.1.2 Points of improvement
Kouijzer et al., 2024	Barrier	“enclosed”	Safety & comfort	Word appears as part of headset discomfort explanation	3.1.2 Points of improvement
Kouijzer et al., 2024	Facilitator	“I find it useful that you can see on the left [of the dashboard] at which step you are and what the next step is. All the expansions are nice. This allows more variations to be made in the VR characters. That is nice for patients to be able to personalize it. I find it user-friendly. It’s easy and even for me it’s doable [to set up a VR scenario].” (H8).	Usability		3.2.1 Positive first impressions
Kouijzer et al., 2024	Facilitator	“My first impression is that it is user-friendly. It is useful and nice that it [setting up a VR scenario] goes step by step. I am surprised about all the options you can choose from. It’ s super comprehensive. I don’t miss anything in terms of environments.” (H9)	Usability		3.2.1 Positive first impressions

Kouijzer et al., 2024	Requirement	"It would be nice if you as a healthcare provider could see a concept of the VR scenario, perhaps by clicking on a special button at the last step, to see what that session will really look like in VR. Then you can see, for example, that the police officer is placed over there and the cashier is really behind the cash register. That you can see a preview of the session that you created before you click 'play'. Then you can easily adjust if something isn't right yet." (H6)	Usability		3.2.2 Points of improvement
Kouijzer et al., 2024	Requirement	"Adding a filter for the characters' profession, age, gender, or length was mentioned."	Usability		3.2.2 Points of improvement
Kouijzer et al., 2024	Barrier	"What I did notice is that the VR environments look too neat. It should be a bit messier to be realistic." (H10)	Usability		3.2.2 Points of improvement
Kouijzer et al., 2024	Barrier	"The stress is a bit higher because someone [a virtual character] is facing me. I know it is not real, but I still have the feeling that I have to deal with him." (P7)	Safety & comfort	Psychological distress during exposure	3.3 Subjective psychological distress
Kouijzer et al., 2024	Barrier	"This experience of uncertainty increased distress and forced patients to be extremely attentive to the situation and their behavior."	Safety & comfort		3.3 Subjective psychological distress
Kouijzer et al., 2024	Facilitator	"Yes, I would definitely like to use this. I would really recommend it to others as well."	Patient acceptability		3.4.1 Willingness to use VR in treatment
Kouijzer et al., 2024	Barrier	"lack of technical skills and experience with innovative technology created a barrier."	Training requirement	Patient capability barrier	3.4.1 Willingness to use VR in treatment
Kouijzer et al., 2024	Barrier	"I prefer to have a real person in front of me. I cannot imagine talking to a virtual person. It just does not feel real. I know this is fake. I find digital and virtual communication harder to understand." (P7).	Patient acceptability		3.4.1 Willingness to use VR in treatment
Kouijzer et al., 2024	Barrier	"difficulty in acting in VR as if it were a real situation, feeling 'insensitive' to the virtual scenarios."	Patient acceptability		3.4.1 Willingness to use VR in treatment
Kouijzer et al., 2024	Facilitator	"It's nice that it's possible to recreate my experiences or what I'm going through in daily life [in VR] and then reflect on that. If you simulate my experiences in the [VR] system, the healthcare providers can see what they normally don't see. You can tell that you fought with someone yesterday, and tell them exactly what happened, but then it is still a guess for that person how it really went. [In VR] you can simulate that situation together and reflect o n i t ." (P7)	Relevance		3.4.2 Treatment possibilities with VR
Kouijzer et al., 2024	Facilitator	"You could use it for role play. For example, if someone is afraid of something, you can expose them to it in a controlled way. I do think that could be useful. You can make someone take a step forward that they might be too afraid of or too shy to do in real-life." (P6)	Relevance		3.4.2 Treatment possibilities with VR
Kouijzer et al., 2024	Facilitator	"If a patient wants to hit someone, at least in VR they hit the air." (P4).	Safety & comfort	Safety of aggression rehearsal	3.4.2 Treatment possibilities with VR
Kouijzer et al., 2024	Facilitator	"I think you can use it for the initial phase and discover personal triggers." (H7)	Relevance		3.5.1 Treatment possibilities with VR
Kouijzer et al., 2024	Facilitator	"I am working with a client on stress reduction. We are looking at what causes him stress, for example, big crowds or loud music around him. With all those triggers I can simulate a scenario in which we can practice relaxation exercises." (H4).	Relevance		3.5.1 Treatment possibilities with VR

Kouijzer et al., 2024	Facilitator	"You could use it for aggression problems. I think most people will start role-playing. For example, when clients feel threatened on the subway if someone stares at them. With aggression, it is often useful to practice management skills, conflict management." (H8)	Relevance		3.5.1 Treatment possibilities with VR
Kouijzer et al., 2024	Facilitator	"You can use it in the exploratory phase, so to discover signals and triggers. After, you can use it as a replacement for the exposure. You can move much faster in the VR world [than in real-life] to apply exposure and set up more behavioral experiments as a result." (H1).	Relevance		3.5.1 Treatment possibilities with VR
Kouijzer et al., 2024	Requirement	"I think they should slowly get used to VR. I think you should explain very well that you first practice with them and go through it together: What can you expect? T hat they have a bit of an idea." (H5).	Training requirement	Onboarding and guided pra	3.5.2 Integrating VR into treatment practice
Kouijzer et al., 2024	Requirement	"It is best to embed it in an existing treatment. I think patients can get used to it slowly. You should explain it very well and go through it together: What it is, what is possible. That they [patients] have an idea and that they can also come up with a situation that you can practice [in VR]". (H5)	Integration in workflow		3.5.2 Integrating VR into treatment practice
Kouijzer et al., 2024	Requirement	"I would not necessarily say in advance that we are going to use VR as standard for everyone. I think that it is very important to look at each and every patient to see whether it fits the treatment goal. Because, for example, with schema therapy treatments you may not need it." (H1)	Relevance	Indication criteria, fit with goals	3.5.2 Integrating VR into treatment practice
Kouijzer et al., 2024	Requirement	"Pay attention to the option to use VR from the start of the treatment. It is important that it is discussed in a team meeting, while the treatment plan is discussed. I think that it is important that patients are informed about this early on in the treatment. That there is a possibility to use VR. Then you can think about it together with the patient." (H2)	Integration in workflow	Early planning, team coordination	3.5.2 Integrating VR into treatment practice
Kouijzer et al., 2024	Barrier	"What I find a barrier in use, is that you have to do a lot. You have to pay attention to the client, control the VR session and you also have to think about what we are going to do." (H10).	Integration in workflow	Cognitive load on provider	3.5.2 Integrating VR into treatment practice
Kouijzer et al., 2024	Requirement	"An introductory video, information brochure, or an online psycho-education module were mentioned by a few healthcare professionals to inform patients and themselves about VR, its possibilities, and its added value."	Training requirement		3.5.3 Implementation materials and activities
Kouijzer et al., 2024	Facilitator	"The managers have to motivate colleagues and say: Well, this is VR therapy and we think this is an important development. People need to be referred to VR as an option for treatment. It needs more attention so that people think; 'Oh, there is a VR set? Nice, I'm using it in my treatment plan." (H3)	Institutional support		3.5.3 Implementation materials and activities
Kouijzer et al., 2024	Requirement	"it is the available time they have to practice together with colleagues on how to use VR technology and apply it to treatment."	Resources & cost	Time as a required resource	3.5.3 Implementation materials and activities

Kouijzer et al., 2024	Requirement	“It just takes a lot of time, training, and practice. You will need to practice this in an intervision with colleagues, because it is mainly a lot of ‘doing, doing, doing’, before you have the self-confidence to say: ‘Oh yes, it works, I can do it.’, and actually apply it to treatment with patients. So, I think this is the biggest investment we have to make.” (H1)	Training requirement	Also relates to resources, but framed as practice/training	3.5.3 Implementation materials and activities
Kouijzer et al., 2024	Requirement	“official training sessions on the use of VR and how to set up scenarios were mentioned.”	Training requirement		3.5.3 Implementation materials and activities
Kouijzer et al., 2024	Requirement	“Certain templates that you make available, which are common situations [written down as a VR exercise] which therapists can then perform. That also gives something to hold on to during treatment.” (H2).	Usability	Templates reduce setup burden	3.5.3 Implementation materials and activities
Lattré et al., 2025	Barrier	“the high initial cost of hardware and software”	Resources & cost		Results – Barriers to the use of VR in hand therapy
Lattré et al., 2025	Barrier	“a lack of awareness of its potential applications in hand therapy”	Evidence & credibility	Awareness linked to perceived legitimacy	Results – Barriers to the use of VR in hand therapy
Lattré et al., 2025	Barrier	“technical difficulties”	Usability		Results – Barriers to the use of VR in hand therapy
Lattré et al., 2025	Barrier	“limited evidence-based research”	Evidence & credibility		Results – Barriers to the use of VR in hand therapy
Lattré et al., 2025	Barrier	“lack of available therapeutic applications”	Usability		Results – Barriers to the use of VR in hand therapy
Lattré et al., 2025	Barrier	“skepticism regarding its clinical benefits”	Evidence & credibility		Results – Barriers to the use of VR in hand therapy
Lattré et al., 2025	Barrier	“the technology is still in its early stages and expected significant developments in the near future”	Institutional support	Organisational readiness signal	Results – Barriers to the use of VR in hand therapy
Lattré et al., 2025	Barrier	“the lack of knowledge regarding how to implement and use VR in hand therapy”	Training requirement	Exact substring from sentence	Discussion
Lattré et al., 2025	Barrier	“the limited number of therapy-specific applications currently available”	Usability	Exact substring from sentence	Discussion
Lattré et al., 2025	Facilitator	“VR usability received a moderate average score of 3.69 /5”	Usability		Discussion
Lattré et al., 2025	Barrier	“only two respondents in this survey mentioned patient experience as an indication for VR use”	Patient acceptability	Low salience, not rejection	Discussion
Lattré et al., 2025	Barrier	“the limited availability of evidence-based research, coupled with ongoing skepticism about VR’s therapeutic value, remains a major obstacle to its routine use”	Evidence & credibility		Discussion
Lattré et al., 2025	Barrier	“Nine percent of respondents cited the lack of scientific evidence as their reason for not adopting VR”	Evidence & credibility		Discussion
Lattré et al., 2025	Requirement	“reducing hardware and software costs”	Resources & cost	Clause from Conclusion sentence	Conclusion
Lattré et al., 2025	Requirement	“expanding the range of therapy-specific applications”	Usability	Clause from Conclusion sentence	Conclusion
Lattré et al., 2025	Requirement	“improving system usability”	Usability	Clause from Conclusion sentence	Conclusion
Lattré et al., 2025	Requirement	“Further high-quality research is needed to evaluate the clinical efficacy of VR”	Evidence & credibility		Conclusion
Lurtz et al., 2024	Facilitator	“the innovational spirit in a university hospital is high.”	Institutional support		Organizational Factors
Lurtz et al., 2024	Facilitator	“Since these environments are often research-focused, financial concerns can take a back seat during pilot phases.”	Resources & cost		Organizational Factors
Lurtz et al., 2024	Facilitator	“This allows for a more exploratory and experimental approach without immediate financial pressures.”	Resources & cost		Organizational Factors
Lurtz et al., 2024	Facilitator	“the inner setting and strong focus around training and education within the hospital, particularly on innovative treatment methods.”	Training requirement		Organizational Factors

Lurtz et al., 2024	Facilitator	“the teaching assignment of the university hospital promotes the adoption of new tools by not merely offering the option to use the device, but by carefully introducing it to the relevant personnel.”	Training requirement	Organizational Factors
Lurtz et al., 2024	Facilitator	“Leadership commitment and management support regarding this and similar projects that go in the direction of digital solutions were explicitly mentioned as a facilitator.”	Institutional support	Organizational Factors
Lurtz et al., 2024	Facilitator	“participants noted that the VR tool might act as a unique selling point of the organization and its ED in clinician recruitment efforts.”	Institutional support	Organizational Factors
Lurtz et al., 2024	Facilitator	“the organizational factor of ‘tension for change’ and the current trend toward such interventions can create traction.”	Relevance	Organizational Factors
Lurtz et al., 2024	Facilitator	“pain management in the ED was confirmed as a pressing concern; having an additional option to alleviate patient pain will facilitate adoption.”	Relevance	Organizational Factors
Lurtz et al., 2024	Barrier	“The required changes in clinical work to implement VR were brought up as a barrier.”	Integration in workflow	Workflow-Related Factors
Lurtz et al., 2024	Barrier	“Workload was mentioned frequently as a main barrier to adoption into the clinical workflow and routine, as time is scarce in the ED.”	Integration in workflow	Workflow-Related Factors
Lurtz et al., 2024	Barrier	“pharmacological options might initially be faster.”	Integration in workflow	Workflow-Related Factors
Lurtz et al., 2024	Facilitator	“a clinician believed that VR may lead to time savings in the long run, benefiting the organization.”	Integration in workflow	Workflow-Related Factors
Lurtz et al., 2024	Facilitator	“the tool could allow them to allocate their time more effectively by distracting patients in pain before further attention is needed.”	Integration in workflow	Workflow-Related Factors
Lurtz et al., 2024	Facilitator	“the tool’s adoption may support the transformation away from purely pharmacological pain management, which was mentioned as a positive change of clinical care.”	Relevance	Workflow-Related Factors
Lurtz et al., 2024	Facilitator	“within the possible simulation environments that the provider supplies, customization is possible in a sense of selection of the preferred environment through the patient, which is a facilitating factor.”	Patient acceptability	Workflow-Related Factors
Lurtz et al., 2024	Facilitator	“especially for nurses, this tool is seen as an opportunity to expand their capabilities and skill sets.”	Institutional support	Workflow-Related Factors
Lurtz et al., 2024	Facilitator	“it is beneficial that nurses can decide to use the VR tool without needing to escalate up the hospital hierarchy or require a physician’s prescription.”	Integration in workflow	Workflow-Related Factors
Lurtz et al., 2024	Facilitator	“Since the tool is already approved and certified, research participants did not perceive regulatory approval as a barrier.”	Legal & ethical alignment	Policy and Regulations
Lurtz et al., 2024	Barrier	“the Swiss insurance system operates on a fee-for-service payment model, where reimbursement is based on the volume of care services provided.”	Resources & cost	Policy and Regulations
Lurtz et al., 2024	Barrier	“This could act as a barrier to adoption for digital health interventions such as the VR tool studied, which focus more on quality and patient experience improvements rather than increasing service volume.”	Resources & cost	Policy and Regulations
Lurtz et al., 2024	Barrier	“as the VR tool subject of this study is applied inside the hospital, the MiGeL-related reimbursement is not applicable.”	Legal & ethical alignment	Policy and Regulations

Lurtz et al., 2024	Barrier	"the categorization as a mandatory service cannot definitely be confirmed according to the policy experts participating in this study."	Legal & ethical alignment		Policy and Regulations
Lurtz et al., 2024	Barrier	"This categorization would rule out options of reimbursement through complementary insurances."	Resources & cost		Policy and Regulations
Lurtz et al., 2024	Barrier	"Further it rules out self-payment of patients, as mandatory services fall under a so-called tariff protection."	Resources & cost		Policy and Regulations
Lurtz et al., 2024	Requirement	"the hospital subject of this study and its physicians, must determine whether the WZW criteria are fulfilled by the tool, by demonstrating effectiveness, appropriateness, and economic efficiency."	Evidence & credibility	WZW criteria (in German: wirksam keit, zweckmässigkeit, und wirtschaftlichkeit; translates to effectiveness, expediency, and efficiency)	Policy and Regulations
Lurtz et al., 2024	Barrier	"this does neither automatically lead to a cost-covering reimbursement amount, nor does it create tariff positions in the TARMED system for 'standard' reimbursement."	Resources & cost		Policy and Regulations
Lurtz et al., 2024	Barrier	"Reimbursement could also be pursued through individual case invoices… However, this approach is resource-intensive and not advisable in this case."	Resources & cost	Ellipsis preserves original wording	Policy and Regulations
Lurtz et al., 2024	Requirement	"other short-term solutions should be explored, such as assessing whether the tool's adoption offers economic advantages, making it attractive even without immediate reimbursement."	Resources & cost		Policy and Regulations
Lurtz et al., 2024	Requirement	"participating policy experts strongly recommend that the hospital engage in advocacy and help shape the new outpatient tariff system."	Legal & ethical alignment		Policy and Regulations
Lurtz et al., 2024	Barrier	"the severity of the patients' pain may impact the tools' effectiveness, as the necessary immersion may be harder to achieve."	Safety & comfort		Patient-Related Factors
Lurtz et al., 2024	Facilitator	"the general difficulty to appropriately treat the condition 'pain' in the ED, including the reluctance for the use of opioids, can be seen as a facilitator."	Relevance		Patient-Related Factors
Lurtz et al., 2024	Barrier	"The nurses in this study highlighted that patient education requires significant time and effort, which might not always be available."	Training requirement		Patient-Related Factors
Lurtz et al., 2024	Facilitator	"The participating patient confirmed the time taken to inform them and noted their high involvement in deciding whether to use the VR tool."	Patient acceptability		Patient-Related Factors
Lurtz et al., 2024	Facilitator	"This process substantially increased patient engagement and their willingness to use the tool."	Patient acceptability		Patient-Related Factors
Lurtz et al., 2024	Facilitator	"nurses may perceive the tool as an expansion of their capabilities, which may have a positive effect on their feelings about their job."	Institutional support		User Engagement
Lurtz et al., 2024	Facilitator	"the interviewed nurses expressed excitement about the application of the new tool."	Institutional support		User Engagement
Lurtz et al., 2024	Barrier	"different nurse team members and physicians individually vary in their perception, which affects teamwork and adoption."	Institutional support	Variability creates local resistance	User Engagement
Lurtz et al., 2024	Facilitator	"User perception was generally positive, and the participating clinicians highlighted the great potential of VR for pain management."	Relevance		Usefulness
Lurtz et al., 2024	Barrier	"installing and applying the tool takes about 20 minutes, which the care professionals must integrate into their already busy schedule."	Integration in workflow		Usefulness
Lurtz et al., 2024	Facilitator	"while the patient is occupied with the tool for 20 minutes, the care team may take advantage of this time more efficiently."	Integration in workflow		Usefulness

Lurtz et al., 2024	Barrier	“explaining the function and usefulness of the device could take significant time, depending on the patient’s prior knowledge.”	Training requirement		Usefulness
Lurtz et al., 2024	Facilitator	“this effort can enhance the patient experience.”	Patient acceptability		Usefulness
Lurtz et al., 2024	Facilitator	“positive stories about the tool increase patients’ willingness to try it.”	Patient acceptability		Usefulness
Lurtz et al., 2024	Requirement	“clear guidelines on how to effectively present the tool to patients may facilitate its adoption.”	Training requirement		Usefulness
Lurtz et al., 2024	Facilitator	“it does not require any additional infrastructure and does not necessitate any integration with other IT systems in the hospital.”	Integration in workflow		IT Capability and Capacity
Lurtz et al., 2024	Facilitator	“these were mostly not applicable to the VR tool in question, as it does not generate health data.”	Legal & ethical alignment		Data-Related Factors
Lurtz et al., 2024	Facilitator	“recent software updates of the visual simulation… made the VR environments more realistic and thereby more immersive and enjoyable.”	Patient acceptability	Ellipsis preserves original wording	User Experience
Lurtz et al., 2024	Facilitator	“The option to select specific scenarios based on personal preferences was especially appreciated by patients.”	Patient acceptability		User Experience
Lurtz et al., 2024	Barrier	“certain medical conditions impacting the mobility of the neck, may not be suitable for the application of VR in a lying position.”	Safety & comfort		User Experience
Lurtz et al., 2024	Facilitator	“users are guided through the application step by step on the tablet steering the VR simulation.”	Usability		Ease of Use
Lurtz et al., 2024	Facilitator	“even without training, a person less familiar with technology can handle the device.”	Usability		Ease of Use
Lurtz et al., 2024	Barrier	“others had issues using the tool, especially in connection with their personal innovativeness.”	Usability		Ease of Use
Lurtz et al., 2024	Barrier	“the lack of technical affinity is mentioned to affect ease-of-use on a personal level.”	Usability		Ease of Use
Lurtz et al., 2024	Barrier	“The lack of a routine in using VR tools was attributed to workload and time constraints.”	Integration in workflow		Ease of Use
Lurtz et al., 2024	Requirement	“patients’ awareness and personal attitudes could be affected through persistent and educative communication.”	Patient acceptability		Personal Characteristics
Lurtz et al., 2024	Facilitator	“Clinicians’ endorsement is key for the acceptance of the tool and may positively influence individual decisions.”	Institutional support		Social and Cultural Factors
Lurtz et al., 2024	Facilitator	“even only one promoting individual physician serving as a VR tool advocate can socially influence others to adopt VR.”	Institutional support		Social and Cultural Factors
Lurtz et al., 2024	Barrier	“When no other team member uses the device, that may therefore inhibit adoption.”	Institutional support		Social and Cultural Factors
Lurtz et al., 2024	Mixed	“younger age was frequently associated with higher comfort and acceptability as well as skills and attitude toward novel technologies.”	Training requirement	Age moderates capability and acceptance	Moderating Factors
Lurtz et al., 2024	Mixed	“older patients generally require more on-boarding time.”	Training requirement		Moderating Factors
Lurtz et al., 2024	Mixed	“male gender was associated with a higher openness to try VR for pain management.”	Patient acceptability		Moderating Factors
Lurtz et al., 2024	Requirement	“an incentive structure within the ED to drive the adoption of new tools, such as VR for pain management.”	Institutional support		Practical Implications
Lurtz et al., 2024	Requirement	“The introduction of a specific ambassador role is proposed.”	Institutional support		Practical Implications
Lurtz et al., 2024	Requirement	“further investigation is proposed in the direction of the potential of the VR tool to support clinical staff in allocating their time as effectively as possible.”	Resources & cost		Practical Implications

Lurtz et al., 2024	Facilitator	“As patients are immersed in VR, this could potentially lead to less simultaneous attention requirements of the nurse.”	Integration in workflow	Practical Implications
Lurtz et al., 2024	Requirement	“investment in advocacy work by the hospital is suggested to shape the upcoming revision of the Swiss tariff system.”	Legal & ethical alignment	Practical Implications
Mondal & Mondal, 2025	Barrier	“lack of access to necessary hardware, such as VR headsets, AR-enabled devices, and high-performance computers”	Resources & cost	Infrastructure Challenges
Mondal & Mondal, 2025	Barrier	“hardware is often outdated or incapable of supporting high-quality AR/VR applications”	Usability	Infrastructure Challenges
Mondal & Mondal, 2025	Barrier	“Power supply instability also hampers AR/VR adoption”	Resources & cost	Infrastructure Challenges
Mondal & Mondal, 2025	Barrier	“medical institutions often lack dedicated spaces for VR-based training”	Integration in workflow	Infrastructure Challenges
Mondal & Mondal, 2025	Barrier	“The cost of AR/VR hardware and software is very high for resource-constrained settings”	Resources & cost	Cost and Affordability
Mondal & Mondal, 2025	Barrier	“The lack of localized AR/VR content is a major barrier to its effective use in education”	Relevance	Lack of Localized Content
Mondal & Mondal, 2025	Barrier	“Many teachers lack the technical skills needed to operate AR/VR devices, navigate software, and troubleshoot issues”	Training requirement	Teacher Training and Awareness
Mondal & Mondal, 2025	Barrier	“The absence of clear national policies prioritizing AR/VR, coupled with limited budgets”	Institutional support	Policy and Institutional Barriers
Mondal & Mondal, 2025	Barrier	“Compatibility issues with older devices and nonstandardized educational platforms”	Usability	Technological Limitations
Mondal & Mondal, 2025	Barrier	“unreliable internet connectivity, particularly in rural and semiurban areas”	Resources & cost	Technological Limitations
Mondal & Mondal, 2025	Barrier	“The digital divide may further limit adoption”	Patient acceptability	Socioeconomic Factors
Mondal & Mondal, 2025	Barrier	“Resistance to change from traditional education systems to newer technologies”	Institutional support	Cultural and Perception Issues
Mondal & Mondal, 2025	Barrier	“the validity and reliability of AR/VR evaluations may create additional barriers”	Evidence & credibility	Assessment and Evaluation
Mondal & Mondal, 2025	Barrier	“Inadequate infrastructure”	Resources & cost	Figure 1
Mondal & Mondal, 2025	Barrier	“High cost”	Resources & cost	Figure 1
Mondal & Mondal, 2025	Barrier	“Lack of localized contents”	Relevance	Figure 1
Mondal & Mondal, 2025	Barrier	“Lack of training”	Training requirement	Figure 1
Mondal & Mondal, 2025	Barrier	“Policy barrier”	Institutional support	Figure 1
Mondal & Mondal, 2025	Barrier	“Technology limitations”	Usability	Figure 1
Mondal & Mondal, 2025	Barrier	“Socio-economic factors”	Resources & cost	Figure 1
Mondal & Mondal, 2025	Barrier	“Cultural barrier”	Institutional support	Figure 1
Mondal & Mondal, 2025	Barrier	“Assessment barrier”	Evidence & credibility	Figure 1
Morgan et al., 2025	Requirement	designing intuitive interfaces for individuals with varying levels of technical proficiency	Usability	Discussion
Morgan et al., 2025	Requirement	ensuring accessibility for individuals with physical disabilities	Usability	Discussion
Morgan et al., 2025	Requirement	the design of experiences for clinically related XR systems must be optimized to prevent fatigue, adverse events, and discomfort during use	Safety & comfort	Discussion
Morgan et al., 2025	Barrier	hardware manufacturers propensity to protect (or limit) access data, and capabilities native to the device itself that may be essential for the implementation of certain use cases	Resources & cost	Discussion
Morgan et al., 2025	Barrier	developing standardized protocols for data flow and compatibility remains a complex set of tasks that has not yet been achieved at scale	Integration in workflow	Discussion

Morgan et al., 2025	Barrier	Protecting sensitive patient information within XR applications necessitates data security measures, which can be both technically demanding and resource-intensive to implement	Legal & ethical alignment		Discussion
Morgan et al., 2025	Barrier	the lack of clear and widely accepted standards	Legal & ethical alignment	Extracted as the explicit causal clause used by the authors	Discussion
Morgan et al., 2025	Barrier	achieving low latency and high performance for real-time interactions is challenging, requiring widespread high-bandwidth connectivity and advanced computing infrastructure that may not be readily available	Resources & cost		Discussion
Morgan et al., 2025	Barrier	complex interfaces and steep learning curves can hinder adoption	Usability		Discussion
Morgan et al., 2025	Requirement	ongoing maintenance and support are vital to ensuring continued functionality, which demand dedicated resources and expertise	Resources & cost		Discussion
Morgan et al., 2025	Barrier	there is also a need for more clinical trials and rigorous research to establish the effectiveness of XR interventions and their impact on patient outcomes	Evidence & credibility		Discussion
Morgan et al., 2025	Barrier	cost considerations, including the initial investment in XR technology, ongoing maintenance, and training for healthcare personnel, must be weighed against the potential benefits	Resources & cost		Discussion
Morgan et al., 2025	Barrier	Budget constraints and the return on investment (ROI) are significant factors influencing the decision to adopt XR in clinical practice	Resources & cost		Discussion
Morgan et al., 2025	Barrier	these regulations can slow down the adoption of innovative technologies and increase the cost and complexity of development and compliance	Legal & ethical alignment		Discussion
Morgan et al., 2025	Barrier	Any slowdowns are particularly costly given the constant stream of new hardware	Resources & cost		Discussion
Morgan et al., 2025	Barrier	Lack of a clear boundary between healthcare and consumer facing applications represents a potentially existential challenge for XR in healthcare startups	Legal & ethical alignment		Discussion
Morgan et al., 2025	Barrier	this ambiguity will likely continue to represent a significant risk for even the most diligent teams developing solutions at the bleeding edge	Legal & ethical alignment		Discussion
Morgan et al., 2025	Barrier	AI carries its own set of complexities and regulatory challenges	Legal & ethical alignment		Discussion
Morgan et al., 2025	Barrier	such efforts will likely have a compounded set of challenges originating from combining both AI and XR	Legal & ethical alignment		Discussion
Morgan et al., 2025	Barrier	without any clear directives this “combination” space runs the risk of being exclusive to large companies	Institutional support	Market access and institutional capacity	Discussion
Morgan et al., 2025	Barrier	Ethical considerations, such as patient consent, privacy, and the potential for XR to alter the patient-provider relationship, also pose significant challenges	Legal & ethical alignment		Discussion
Morgan et al., 2025	Barrier	Ensuring robust consent processes and maintaining patient privacy are crucial for ethical deployment, but they can also be resource-intensive and can complicate implementation	Legal & ethical alignment		Discussion
Morgan et al., 2025	Barrier	there is a risk of it depersonalizing care or creating dependency on technology	Patient acceptability		Discussion
Morgan et al., 2025	Barrier	PHI, institutional policies, interdepartmental communication and coordination	Legal & ethical alignment		Table 2 – Considerations

Morgan et al., 2025	Barrier	Tether vs. Standalone, Form Factor, Enterprise “friendliness”, Agnosticism & Lock-in	Resources & cost		Table 2 – Considerations
Morgan et al., 2025	Barrier	greater performance comes at a higher price point which may limit your application in its ability to scale	Resources & cost		Table 2 – Hardware specifications
Morgan et al., 2025	Barrier	Data Security, Privacy, Clinical utility, Interoperability, Patient and provider access to the data	Legal & ethical alignment		Table 2 – Considerations
Morgan et al., 2025	Requirement	AI outputs that are central to the clinical value-add may have a higher level of diligence required compared to AI outputs being used for an ancillary purpose	Evidence & credibility		Table 2 – In combination with AI
Pereira Guerreiro et al., 2025	Facilitator	“integration with resident care systems contributes to streamlining the care process and humanising care for this vulnerable population.”	Integration in workflow	Explicit workflow and system-level efficiency enabler	Conclusion
Pereira Guerreiro et al., 2025	Requirement	“Training of formal caregivers will be online and self-paced, with the duration of 30–45 min.”	Training requirement	Practical training format affecting feasibility and uptake	Stage 2: Usability study
Pereira Guerreiro et al., 2025	Barrier	“This may affect the generalisability of our findings, as the selected sample is unlikely to represent the broader population of residential care facilities in Portugal.”	Evidence & credibility	Limits scalability and external validity	Discussion
Pereira Guerreiro et al., 2025	Barrier	“the absence of a control group in our study design means that we are unable to determine whether potential changes are attributable to the adoption of the PainChek® App or to external factors or variations over time.”	Evidence & credibility	Constrains causal attribution of adoption effects	Discussion
Pereira Guerreiro et al., 2025	Requirement	“Benefits associated with the PainChek® App, which warrant further research, can only be realised through successful adoption of this technology.”	Relevance	Adoption explicitly framed as a necessary condition	Conclusion
Sarkar et al., 2021	Facilitator	“a strong sense of an unmet need for chronic pain management”	Relevance		Results
Sarkar et al., 2021	Facilitator	“addressing pain management differently represents a high organizational/clinical priority”	Relevance		Results
Sarkar et al., 2021	Facilitator	“the favorable safety of using VR compared to pain medications”	Safety & comfort		Characteristics of the Intervention
Sarkar et al., 2021	Facilitator	“the option for patients to use the technology on their own on an ongoing basis after initial training from staff”	Integration in workflow	Relates to self-management and reduced clinician dependency	Characteristics of the Intervention
Sarkar et al., 2021	Facilitator	“the potential for scaling pain management activities through VR-enabled self-management approaches”	Integration in workflow		Characteristics of the Intervention
Sarkar et al., 2021	Facilitator	“the face validity of VR as a chronic pain treatment”	Evidence & credibility	Perceived credibility rather than formal evidence	Characteristics of the Intervention
Sarkar et al., 2021	Barrier	“the platform is available only in English and therefore not usable in limited English–proficiency populations”	Patient acceptability		Characteristics of the Intervention
Sarkar et al., 2021	Barrier	“the content’s appropriateness for culturally diverse populations and for those with significant trauma histories”	Patient acceptability		Characteristics of the Intervention
Sarkar et al., 2021	Barrier	“the currently available content does not always resonate across cultures, particularly for minoritized populations”	Patient acceptability		Characteristics of the Intervention
Sarkar et al., 2021	Facilitator	“the ability of VR to offer many culturally tailored content offerings”	Patient acceptability		Characteristics of the Intervention
Sarkar et al., 2021	Barrier	“the specific contraindication of motion sickness”	Safety & comfort		Characteristics of the Intervention
Sarkar et al., 2021	Facilitator	“the overall low risk of the modality”	Safety & comfort		Characteristics of the Intervention
Sarkar et al., 2021	Barrier	“challenges for older adults in navigating technology”	Usability		Individual Characteristics
Sarkar et al., 2021	Barrier	“digital literacy more broadly”	Usability		Individual Characteristics
Sarkar et al., 2021	Barrier	“patient mistrust of new or experimental treatments”	Patient acceptability		Individual Characteristics
Sarkar et al., 2021	Facilitator	“near-universal high levels of patient satisfaction with VR”	Patient acceptability		Individual Characteristics
Sarkar et al., 2021	Barrier	“physical opioid dependence and a fear of opioid de-prescribing”	Patient acceptability		Individual Characteristics

Sarkar et al., 2021	Barrier	“patients’ more pressing social and health needs as significant barriers to VR implementation for pain”	Integration in workflow	Social context interfering with engagement	Individual Characteristics
Sarkar et al., 2021	Requirement	“patients would require specific orientation from staff in order to initiate VR use for pain”	Training requirement		Implementation Process
Sarkar et al., 2021	Requirement	“staff support would be required for coaching and troubleshooting on an ongoing basis”	Training requirement		Implementation Process
Sarkar et al., 2021	Requirement	“the requirement that frontline staff interact with VR and have a personal buy-in to facilitate successful implementation”	Institutional support	Buy-in framed as prerequisite	Implementation Process
Sarkar et al., 2021	Requirement	“the need for champions among clinicians who can share both evidence for VR as a pain treatment and successful treatment experiences”	Institutional support		Implementation Process
Sarkar et al., 2021	Barrier	“The availability of staff time was a dominant concern”	Resources & cost	Time treated as a constrained resource	Implementation Process
Sarkar et al., 2021	Barrier	“Integration of VR into existing pain management workflows”	Integration in workflow	Framed as challenge rather than neutral descriptor	Implementation Process
Sarkar et al., 2021	Requirement	“implementation success would depend on the extent to which VR could be integrated into specific clinical settings”	Integration in workflow		Implementation Process
Sarkar et al., 2021	Barrier	“the intervention requires a multistep process with each patient, which both users and nonusers believe complicates more widespread implementation”	Integration in workflow		Implementation Process
Sarkar et al., 2021	Barrier	“pain management is a cross-cutting issue and therefore requires collaboration among departments and stakeholders”	Integration in workflow	Organisational coordination burden	Inner Setting
Sarkar et al., 2021	Barrier	“pressure to prescribe opioids”	Institutional support	Reflects systemic norms	Inner Setting
Sarkar et al., 2021	Barrier	“the providers’ lack of familiarity with VR”	Training requirement		Inner Setting
Sarkar et al., 2021	Barrier	“the default habit and culture of addressing pain with medication”	Institutional support	Cultural resistance	Inner Setting
Sarkar et al., 2021	Facilitator	“the importance of leadership attitudes”	Institutional support		Inner Setting
Sarkar et al., 2021	Facilitator	“openness to innovation”	Institutional support		Inner Setting
Sarkar et al., 2021	Requirement	“the need for dedicated staff time and training and space for VR treatment”	Resources & cost	Multiple resource dimensions in one verbatim phrase	Inner Setting
Sarkar et al., 2021	Barrier	“Resource limitations in safety-net settings were seen to preclude these needed actions for implementation”	Resources & cost		Inner Setting
Sarkar et al., 2021	Requirement	“an innovative culture is a prerequisite for VR implementation”	Institutional support		Inner Setting
Sarkar et al., 2021	Requirement	“the need for adequate physical space for VR treatment”	Resources & cost		Inner Setting
Sarkar et al., 2021	Requirement	“up-front investment in the VR hardware”	Resources & cost		Inner Setting
Sarkar et al., 2021	Barrier	“use of VR is not a billable service”	Resources & cost		Outer Setting
Sarkar et al., 2021	Barrier	“lack of insurance reimbursement as a critical barrier to implementation at scale”	Resources & cost		Outer Setting
Sarkar et al., 2021	Barrier	“expecting patients to bear the cost of pain treatment is a significant implementation barrier”	Resources & cost		Outer Setting
Sarkar et al., 2021	Barrier	“concerns around data privacy and security in digital health”	Legal & ethical alignment		Outer Setting
Sarkar et al., 2021	Barrier	“competing demands in patients’ lives, relating to housing status, income instability, and other social challenges, would interfere with using a novel therapeutic approach like VR”	Integration in workflow	Social determinants interfering with uptake	Discussion
Sarkar et al., 2021	Requirement	“the need for cultural tailoring and translation”	Patient acceptability		Discussion
Sarkar et al., 2021	Requirement	“deeper qualitative exploration with patients about their preferences or comfort in using VR headsets”	Patient acceptability		Discussion

Sarkar et al., 2021	Requirement	“usability testing with diverse populations could evaluate concerns about the acceptability and usability of technology, disinfection, and adverse effects such as motion sickness”	Evidence & credibility	Methodological credibility and safety evidence	Discussion
Sarkar et al., 2021	Requirement	“Any deployment strategy for VR should take into account the resource and workforce constraints of the safety-net environment”	Resources & cost		Discussion
Schreiter et al., 2025	Barrier	“No previous VR experience”	Training requirement	Prior exposure is treated as a capability precondition in this paper’s results	Table 2. Results of the prior conditions.
Schreiter et al., 2025	Facilitator	“Previous VR experience”	Training requirement		Table 2. Results of the prior conditions.
Schreiter et al., 2025	Barrier	“External stakeholder”	Institutional support	External actors include insurers, legislation, organisation in their definition	Table 2. Results of the prior conditions.
Schreiter et al., 2025	Barrier	“market uncertainties”	Resources & cost		Table 2. Results of the prior conditions.
Schreiter et al., 2025	Barrier	“IT infrastructure”	Integration in workflow	Infrastructure is described as an implementation constraint	Table 2. Results of the prior conditions.
Schreiter et al., 2025	Barrier	“Awareness knowledge of the existence of an innovation”	Evidence & credibility	In this paper, awareness is treated as “being informed” that the innovation exists	Table 4. Results of the knowledge of health care professionals.
Schreiter et al., 2025	Facilitator	“Effectiveness”	Evidence & credibility		Table 5. Results of the characteristics of virtual reality (VR) technologies.
Schreiter et al., 2025	Facilitator	“Efficiency”	Integration in workflow	Efficiency links to time and operational flow	Table 5. Results of the characteristics of virtual reality (VR) technologies.
Schreiter et al., 2025	Facilitator	“Diversification”	Relevance	Portfolio expansion and patient-centred tailoring	Table 5. Results of the characteristics of virtual reality (VR) technologies.
Schreiter et al., 2025	Barrier	“Affordability”	Resources & cost	Cost concerns are described as adoption-relevant	Table 5. Results of the characteristics of virtual reality (VR) technologies.
Schreiter et al., 2025	Barrier	“Data security”	Legal & ethical alignment		Table 5. Results of the characteristics of virtual reality (VR) technologies.
Schreiter et al., 2025	Barrier	“Complexity of VR hardware”	Usability		Table 5. Results of the characteristics of virtual reality (VR) technologies.
Schreiter et al., 2025	Barrier	“Complexity of VR software”	Usability		Table 5. Results of the characteristics of virtual reality (VR) technologies.
Schreiter et al., 2025	Requirement	“Trialability”	Usability	Treated as need to test before adoption	Table 5. Results of the characteristics of virtual reality (VR) technologies.
Schreiter et al., 2025	Barrier	“Lack of evidence-based studies”	Evidence & credibility		Table 5. Results of the characteristics of virtual reality (VR) technologies.
Schreiter et al., 2025	Facilitator	“Measurability of results with VR technologies”	Evidence & credibility		Table 5. Results of the characteristics of virtual reality (VR) technologies.
Schreiter et al., 2025	Barrier	“Maybe It [VR technologies] could fail in the sense that patients do not accept it, that they just say that it does not do anything for me or that confuses me, or I do not know how to do it right there.”	Patient acceptability		Norms of the Social System
Schreiter et al., 2025	Facilitator	“if you explain it [VR technologies] to them well and make it palatable...I do not think [patients] are then averse to it.”	Patient acceptability		Norms of the Social System
Schreiter et al., 2025	Facilitator	“They [patients] are open to it. If I sit in front of them and say we are doing therapy today with VR technologies, and it can be an 80-year-old grandma, she does not say no...”	Patient acceptability		Norms of the Social System
Schreiter et al., 2025	Facilitator	“would not see it [age] as a barrier, but rather as an opportunity”	Patient acceptability	This is explicitly about age as not being a barrier	Norms of the Social System
Schreiter et al., 2025	Barrier	“those who are not so technically skilled...so with over-60s, over-70s, over-80s.”	Patient acceptability		Norms of the Social System
Schreiter et al., 2025	Facilitator	“is only a matter of time [before] VR glasses are in our house too”	Institutional support	Diffusion expectation linked to team context	Norms of the Social System
Schreiter et al., 2025	Facilitator	“I always present such newer things to the team [and] the decision is then also [made] in the team.”	Institutional support		Norms of the Social System
Schreiter et al., 2025	Barrier	“Otherwise, we would have made the investment [in VR technologies] a long time ago.”	Resources & cost	Points to funding/reimbursement as constraint	Norms of the Social System
Schreiter et al., 2025	Barrier	“...[it] was also not yet required [from the clinic] to be done that way.”	Institutional support	Lack of organisational requirement	Norms of the Social System
Schreiter et al., 2025	Facilitator	“Who is sitting there in the rehabilitation clinics with an open mind?...and how open is the [CEO] really to innovation?”	Institutional support	Leadership openness	Norms of the Social System

Schreiter et al., 2025	Facilitator	“We wanted to remain competitive as a unique selling point...Of course, it is also special when I am looking for an employee and have VR glasses there.”	Institutional support	Recruitment/positioning motive	Felt Needs and Problems
Schreiter et al., 2025	Barrier	“a big barrier overall is implementing it [VR technologies] into existing structures because they have to be flexible and kind of make room”	Integration in workflow		Felt Needs and Problems
Schreiter et al., 2025	Barrier	“spatial requirements”	Integration in workflow	Physical space constraint	Felt Needs and Problems
Schreiter et al., 2025	Requirement	“[the] integration of VR technologies should result in an overall concept”	Integration in workflow		Felt Needs and Problems
Schreiter et al., 2025	Barrier	“lack of motivation...as the biggest barrier””	Institutional support	Motivation is discussed as an adoption driver among staff	Innovativeness
Schreiter et al., 2025	Barrier	“There was a lot of fear: Oh God, so much technology. I cannot do this anyway. It is just too technically demanding for me.”	Training requirement		Innovativeness
Schreiter et al., 2025	Barrier	“I sometimes have a hard time with the latest innovations and with technology in general. That is why it takes me a little longer [and] when I can, I like to skip things like [VR technologies].”	Training requirement		Innovativeness
Schreiter et al., 2025	Barrier	“a lack of being informed about it [VR technologies]”	Evidence & credibility	Knowledge gap, framed as obstacle	Knowledge of Health Care Professionals
Schreiter et al., 2025	Barrier	“biggest obstacles [to successful adoption]”	Evidence & credibility	Refers to missing awareness/information	Knowledge of Health Care Professionals
Schreiter et al., 2025	Requirement	“clearing-up work”	Training requirement	Education work as required action	Knowledge of Health Care Professionals
Schreiter et al., 2025	Requirement	“intensified medium appearance...in the sense of television contributions”	Institutional support	Dissemination via media channels	Knowledge of Health Care Professionals
Schreiter et al., 2025	Requirement	“rehabilitation magazines”	Institutional support	Dissemination channel	Knowledge of Health Care Professionals
Schreiter et al., 2025	Requirement	“education”	Training requirement		Knowledge of Health Care Professionals
Schreiter et al., 2025	Requirement	“training courses”	Training requirement		Knowledge of Health Care Professionals
Schreiter et al., 2025	Barrier	“additional screen time”	Safety & comfort		Relative Advantage
Schreiter et al., 2025	Barrier	“various children and adolescents becoming addicted”	Safety & comfort		Relative Advantage
Schreiter et al., 2025	Barrier	“body awareness is lost”	Safety & comfort		Relative Advantage
Schreiter et al., 2025	Barrier	“headaches”	Safety & comfort		Relative Advantage
Schreiter et al., 2025	Barrier	“eye pain”	Safety & comfort		Relative Advantage
Schreiter et al., 2025	Barrier	“nausea”	Safety & comfort		Relative Advantage
Schreiter et al., 2025	Barrier	“dizziness”	Safety & comfort		Relative Advantage
Schreiter et al., 2025	Barrier	“motion sickness”	Safety & comfort		Relative Advantage
Schreiter et al., 2025	Barrier	“seems to initially involve more work”	Integration in workflow	Training/setup burden phrased as extra work	Efficiency
Schreiter et al., 2025	Barrier	“The time investment required to engage with the technology is a tragic hurdle.”	Integration in workflow		Efficiency
Schreiter et al., 2025	Facilitator	“autonomous patient therapy from home without supervision”	Integration in workflow		Efficiency
Schreiter et al., 2025	Facilitator	“group therapy”	Integration in workflow		Efficiency
Schreiter et al., 2025	Facilitator	“automated documentation and measurement”	Integration in workflow		Efficiency
Schreiter et al., 2025	Barrier	“very dauntingly high”	Resources & cost		Affordability
Schreiter et al., 2025	Facilitator	“one of the smallest investments [made] here in practice compared to other therapy methods”	Resources & cost		Affordability
Schreiter et al., 2025	Barrier	“development costs”	Resources & cost		Affordability
Schreiter et al., 2025	Barrier	“spare parts”	Resources & cost		Affordability
Schreiter et al., 2025	Facilitator	“leasing options”	Resources & cost		Affordability
Schreiter et al., 2025	Barrier	“As soon as anything is digitally innovative, [data security] always comes right to my mind.”	Legal & ethical alignment		Data security
Schreiter et al., 2025	Facilitator	“nowadays...data privacy must be accepted everywhere. That should be the lesser inconvenience”	Legal & ethical alignment		Data security
Schreiter et al., 2025	Facilitator	“...it does not store any data. I do not have to enter a name... just press the button and go.”	Legal & ethical alignment		Data security
Schreiter et al., 2025	Requirement	“user-friendly, intuitive, and robust against user error”	Usability		Complexity
Schreiter et al., 2025	Requirement	“...that if you are not that computer literate, you can get on with it quickly...”	Usability		Complexity

Schreiter et al., 2025	Barrier	"can lead to failure [of VR technologies]"	Usability		Complexity
Schreiter et al., 2025	Barrier	"My biggest concern is the applicability, whether this concept works well or still seems very, very beginning...[then it may be] I bought it [VR technologies] but will never use it."	Relevance	Applicability is framed as the make-or-break adoption issue	Complexity
Schreiter et al., 2025	Requirement	"transportable"	Resources & cost	Operational requirement for deployment	Complexity
Schreiter et al., 2025	Requirement	"light"	Safety & comfort	Physical burden/ergonomics	Complexity
Schreiter et al., 2025	Requirement	"comfortable"	Safety & comfort		Complexity
Schreiter et al., 2025	Requirement	"robust"	Usability	Reliability requirement	Complexity
Schreiter et al., 2025	Barrier	"too heavy"	Safety & comfort		Complexity
Schreiter et al., 2025	Barrier	"the cables...[can] be a difficulty"	Usability		Complexity
Schreiter et al., 2025	Barrier	"hurt on the nose"	Safety & comfort		Complexity
Schreiter et al., 2025	Barrier	"field of view [is] still very limited...and partly a bit blurry"	Usability	Visual fidelity affects use	Complexity
Schreiter et al., 2025	Requirement	"It would be good to test it [VR technology] beforehand."	Usability		Trialability
Schreiter et al., 2025	Facilitator	"results can be seen directly"	Evidence & credibility		Observability
Schreiter et al., 2025	Facilitator	"...range of motion, mobility...you can measure it very well."	Evidence & credibility		Observability
Schreiter et al., 2025	Barrier	"they [VR technologies] are not researched or proven with studies at all how much this does"	Evidence & credibility		Observability
Schreiter et al., 2025	Barrier	"person to person...so that the social component is not missing"	Integration in workflow	Value-based compatibility with care model	Compatibility
Schreiter et al., 2025	Barrier	"Maybe it also fails because of [lack of] connectivity."	Integration in workflow	Interoperability constraint	Compatibility
Schreiter et al., 2025	Requirement	"[should] be compatible with other devices."	Integration in workflow		Compatibility
Schreiter et al., 2025	Barrier	"does not have twelve operating systems."	Integration in workflow	System fragmentation concern	Compatibility
Shiner et al., 2024	Barrier	"Cost"	Resources & cost		Table 4
Shiner et al., 2024	Barrier	"Insufficient IT support"	Institutional support		Table 4
Shiner et al., 2024	Barrier	"Insufficient clinician skill/knowledge to operate VR"	Training requirement		Table 4
Shiner et al., 2024	Barrier	"Lack of clinician time to learn how to use VR"	Integration in workflow		Table 4
Shiner et al., 2024	Barrier	"Additional time required to use VR in a treatment session"	Integration in workflow		Table 4
Shiner et al., 2024	Barrier	"Patient resistance or reluctance"	Patient acceptability		Table 4
Shiner et al., 2024	Barrier	"Insufficient or no available treatment space/s to use VR"	Integration in workflow		Table 4
Shiner et al., 2024	Barrier	"Insufficient or poor quality evidence to support the use of VR"	Evidence & credibility		Table 4
Shiner et al., 2024	Barrier	"Safety concerns (such as infection and/or injury risk)"	Safety & comfort		Table 4
Shiner et al., 2024	Barrier	"Lack of suitability for some patient groups (such as older adults, those with cognitive impairment, vestibular problems)"	Patient acceptability		Table 4
Shiner et al., 2024	Barrier	"Clinicians not interested in using VR technology"	Institutional support		Table 4
Shiner et al., 2024	Barrier	"Concerns for VR technology replacing the role of clinicians"	Institutional support		Table 4
Shiner et al., 2024	Barrier	"resistance to change"	Institutional support	Other barriers	Table 4
Shiner et al., 2024	Barrier	"lack of organisational and/or senior leadership support"	Institutional support	Other barriers	Table 4
Shiner et al., 2024	Barrier	"uncertainty around clinical benefits"	Evidence & credibility	Other barriers	Table 4
Shiner et al., 2024	Barrier	"concern that VR would reduce human interaction between patients and care providers to negatively impact rapport"	Patient acceptability	Other barriers	Table 4
Shiner et al., 2024	Facilitator	"Training courses for clinical staff on VR use"	Training requirement		Table 4
Shiner et al., 2024	Facilitator	"Designated IT support personnel"	Institutional support		Table 4
Shiner et al., 2024	Facilitator	"Access to portable and/or wireless VR technology to use in hospital (such as Smartphone VR applications)"	Integration in workflow		Table 4
Shiner et al., 2024	Facilitator	"Designated VR treatment space in the hospital"	Integration in workflow		Table 4
Shiner et al., 2024	Facilitator	"Clinical evidence summaries or guidelines on VR use"	Evidence & credibility		Table 4

Shiner et al., 2024	Facilitator	"Access to VR software developers and/or design experts"	Resources & cost		Table 4
Shiner et al., 2024	Facilitator	"Local VR champions within the clinical setting"	Institutional support		Table 4
Shiner et al., 2024	Facilitator	"The ability to record and store data for tracking patient progression"	Resources & cost		Table 4
Shiner et al., 2024	Facilitator	"The ability to develop customised VR programs/software"	Resources & cost		Table 4
Shiner et al., 2024	Facilitator	"Instructional brochures or pamphlets"	Training requirement		Table 4
Shiner et al., 2024	Facilitator	"Subsidised access to VR devices"	Resources & cost		Table 4
Shiner et al., 2024	Facilitator	"Financial incentives for VR treatment"	Resources & cost		Table 4
Shiner et al., 2024	Facilitator	"organisational endorsement from senior leadership"	Institutional support	Other enablers	Table 4
Shiner et al., 2024	Facilitator	"development of a hospital-wide policy or strategy"	Institutional support	Other enablers	Table 4
Shiner et al., 2024	Barrier	"modest familiarity with VR technology"	Training requirement		Results – Knowledge of and familiarity with VR technology
Shiner et al., 2024	Barrier	"had never personally used or engaged with VR before"	Training requirement		Results – Knowledge of and familiarity with VR technology
Shiner et al., 2024	Barrier	"being aware of any research or clinical evidence that supported the use of VR in a clinical or hospital setting"	Evidence & credibility		Results – Knowledge of and familiarity with VR technology
Shiner et al., 2024	Facilitator	"they believed VR technology would become routinely implemented within hospital environments"	Institutional support		Results – Knowledge of and familiarity with VR technology
Shiner et al., 2024	Barrier	"time constraints for clinicians"	Integration in workflow		Results – Perceived barriers and enablers of VR use
Shiner et al., 2024	Barrier	"limited physical space within the hospital environment"	Integration in workflow		Results – Perceived barriers and enablers of VR use
Shiner et al., 2024	Barrier	"limited operational knowledge of VR among staff"	Training requirement		Results – Perceived barriers and enablers of VR use
Terkildsen et al., 2024	Facilitator	"VR's current technological maturity and affordability were seen as having been achieved in a few years, and it was expected that the technology and price would only improve."	Resources & cost		Results
Terkildsen et al., 2024	Facilitator	"All informants expected VR to be a significant part of the future mental health care system."	Institutional support		Results
Terkildsen et al., 2024	Requirement	"It makes little sense to develop a solution for a specific department. Then a different department also has to develop the same solution. So, in that way, we have a fundamental view always to consider whether there is an opportunity to test this in some ways so that more people can benefit from what is being made."	Institutional support		Results – Diffusion logic
Terkildsen et al., 2024	Facilitator	"We have taken this approach to new technologies, where we come up with them and then say, how can we use them and get them into our healthcare sector? What happens when we do that? Instead of us just sitting and waiting for someone to think that VR could be a solution."	Institutional support		Results – Diffusion logic
Terkildsen et al., 2024	Requirement	"Still, we must constantly be able to spread it out in our colossal organisation across the Region."	Institutional support		Results – Diffusion logic
Terkildsen et al., 2024	Requirement	"It is about organisational learning. The way we get it out there. That is to say, there is a place that does a project, then you do a trial operation, and then you have to spread it out so that it is used everywhere. And that is– that must be the goal."	Institutional support		Results – Diffusion logic
Terkildsen et al., 2024	Barrier	"implementation requires more than development so that it is implemented appropriately in the way it is intended. Because otherwise, it will be 117 different versions."	Integration in workflow		Results – Diffusion logic

Terkildsen et al., 2024	Barrier	“Individual healthcare professional staff and ‘activists’ were considered a potential barrier to the overall organisational adoption process by focusing too much on their specific clinical context and thereby not seeing the bigger organisational picture.”	Institutional support	Results – Diffusion logic
Terkildsen et al., 2024	Barrier	“it is often entirely random who you partner with and what solutions you see.”	Institutional support	Results – Diffusion logic
Terkildsen et al., 2024	Requirement	“Selecting highly skilled providers that were also deemed financially resilient and could be expected to deliver support and upgrades for years was essential.”	Resources & cost	Results – Diffusion logic
Terkildsen et al., 2024	Barrier	“solutions based on smaller companies (often expressed as one man in a basement) were less desirable:”	Resources & cost	Results – Diffusion logic
Terkildsen et al., 2024	Barrier	“the health care system was under pressure from a lack of finances and how developing local VR solutions from scratch was considered an expensive ordeal:”	Resources & cost	Results – Diffusion logic
Terkildsen et al., 2024	Barrier	“So it’s also the fact that it’s an expensive technology when we have to make these recordings from scratch.”	Resources & cost	Results – Diffusion logic
Terkildsen et al., 2024	Requirement	“The development and adoption of VR for mental health care always needed to consider the economy.”	Resources & cost	Results – Diffusion logic
Terkildsen et al., 2024	Barrier	“The logic of diffusion promoted an understanding of local VR developments as economically unsustainable if developed and adopted without focusing on the organisation as a whole.”	Resources & cost	Results – Diffusion logic
Terkildsen et al., 2024	Requirement	“It also included a focus on the importance of the establishment of central IT competencies.”	Institutional support	Results – Diffusion logic
Terkildsen et al., 2024	Facilitator	“VR technology was understood first and foremost as a tool that would afford new ways of helping specific patient groups or improving clinical practices through education in a particular setting.”	Relevance	Results – Professional logic
Terkildsen et al., 2024	Requirement	“That is why you must also address when you want to use VR, how, which patients, and what you hope to achieve.”	Relevance	Results – Professional logic
Terkildsen et al., 2024	Requirement	“It is the clinicians who present the problem and say, we think VR might be a solution to this clinical problem, and we need this kind of content”	Relevance	Results – Professional logic
Terkildsen et al., 2024	Requirement	“the unique features of each group of end-users required constant vigilance and focus on detail to ensure that VR also responded appropriately to such features:”	Usability	Results – Professional logic
Terkildsen et al., 2024	Barrier	“Neglecting a strict focus on particularity and choosing a generic solution across end-user groups was, on the other hand, seen as putting technology ahead of the user or dancing after the technology and, therefore, the wrong path to pursue.”	Relevance	Results – Professional logic
Terkildsen et al., 2024	Requirement	“the development and adoption process should ensure measurable and objective improvement for the strictly defined end-user groups.”	Evidence & credibility	Results – Professional logic
Terkildsen et al., 2024	Requirement	“the fundamental understanding of scientific evidence as the foundation of treatment and education in the healthcare sector and, therefore, as a critical mechanism for the adoption process.”	Evidence & credibility	Results – Professional logic
Terkildsen et al., 2024	Requirement	“then it must be driven by a clinical problem where you think this could perhaps do something different for my patients. It could help my patients in a new way.”	Relevance	Results – Professional logic

Terkildsen et al., 2024	Requirement	"there is a procedure where we assess the patient, and we assess, is this the right thing for them?"	Safety & comfort	Results – Professional logic
Terkildsen et al., 2024	Requirement	"And that's why it (VR) also requires research to ensure that you register the side effects when you do that research."	Safety & comfort	Results – Professional logic
Terkildsen et al., 2024	Barrier	"Implementation before research? It makes no sense? Now it is the goggle and then the patient."	Evidence & credibility	Results – Professional logic
Terkildsen et al., 2024	Barrier	"Such differences can cause friction that may hinder the adoption process if not accommodated adequately."	Institutional support	Discussion
Terkildsen et al., 2024	Requirement	"calling organisations wanting to adopt VR in various health care setting to actively provide collaborative venues for participating stakeholders in which their differences are made the subject of joint reflection."	Institutional support	Discussion
Terkildsen et al., 2024	Barrier	"experienced several incidents of COVID-19 shutdown, causing several periods of stand-by where they could not work on their projects."	Integration in workflow	Strengths and limitations
Terkildsen et al., 2024	Barrier	"many initiatives not directly related to COVID-19, to an extent, may have received lesser attention from FS and ASS."	Institutional support	Strengths and limitations
Terkildsen et al., 2024	Barrier	"different institutional logics may collide, causing friction and challenges."	Institutional support	Conclusion
UCL & KCL, 2023	Barrier	"buy-in from senior decision makers and wider awareness of the technology limits implementation"	Institutional support	What we learned from interviewing simulation practitioners
UCL & KCL, 2023	Barrier	"Resources for XR implementation are very limited"	Resources & cost	What we learned from interviewing simulation practitioners
UCL & KCL, 2023	Barrier	"There is limited implementation of XR technologies within London"	Institutional support	What we learned from interviewing simulation practitioners
UCL & KCL, 2023	Requirement	"learners require guidance when first using a VR environment"	Training requirement	The perceived benefits of XR
UCL & KCL, 2023	Requirement	"investing in the technology and technology knowledgeable staff"	Training requirement	The perceived benefits of XR
UCL & KCL, 2023	Barrier	"High cost"	Resources & cost	Barriers and Facilitators of XR Implementation
UCL & KCL, 2023	Barrier	"Poor quality software"	Usability	Barriers and Facilitators of XR Implementation
UCL & KCL, 2023	Barrier	"Staff attitutes"	Institutional support	Barriers and Facilitators of XR Implementation
UCL & KCL, 2023	Barrier	"Limited evidence of effectiveness"	Evidence & credibility	Barriers and Facilitators of XR Implementation
UCL & KCL, 2023	Barrier	"IT systems"	Integration in workflow	Barriers and Facilitators of XR Implementation
UCL & KCL, 2023	Barrier	"Limited workforce support for XR modalities"	Resources & cost	Barriers and Facilitators of XR Implementation
UCL & KCL, 2023	Barrier	"Poor quality software (with limited off the shelf scenarios)"	Usability	Barriers to XR Implementation
UCL & KCL, 2023	Barrier	"Staff attitudes and a resistance to change"	Institutional support	Barriers to XR Implementation
UCL & KCL, 2023	Barrier	"The Limited evidence of the effectiveness of XR modalities"	Evidence & credibility	Barriers to XR Implementation
UCL & KCL, 2023	Barrier	"Both hardware and software were identified as being expensive and challenging to fund"	Resources & cost	Barriers to XR Implementation
UCL & KCL, 2023	Barrier	"Slow IT approvals for software and equipment utilisation was a source of delay"	Integration in workflow	Barriers to XR Implementation
UCL & KCL, 2023	Barrier	"Limited resources for healthcare education"	Institutional support	Barriers to XR Implementation
UCL & KCL, 2023	Barrier	"the lack of technically skilled staff to drive the implementation of XR technologies"	Training requirement	Barriers to XR Implementation
UCL & KCL, 2023	Facilitator	"Comprehension of the modality"	Training requirement	Facilitators of Implementation
UCL & KCL, 2023	Facilitator	"Buy in from colleagues and executive"	Institutional support	Facilitators of Implementation
UCL & KCL, 2023	Facilitator	"Networking opportunities"	Institutional support	Facilitators of Implementation
UCL & KCL, 2023	Facilitator	"Central coordination of XR procurement"	Institutional support	Facilitators of Implementation
UCL & KCL, 2023	Facilitator	"a Skilled technical team"	Institutional support	Facilitators of Implementation
UCL & KCL, 2023	Facilitator	"exposure to XR being identified as the antidote to resistance"	Training requirement	Barriers and Facilitators of XR Implementation

Zhang et al., 2020	Requirement	"It is recommended that some assessment methods should favor the involvement of specific user groups, such as external users (ie, a group of testers not involved in the development process)."	Evidence & credibility	Requirement for external validation	VR Usability Assessment Methods – Overview
Zhang et al., 2020	Requirement	"Some assessment method requirements also lend themselves to requiring representative users, meaning a sample of users who may reflect the appropriate end-user population."	Evidence & credibility	Addresses sampling validity	VR Usability Assessment Methods – Overview
Zhang et al., 2020	Requirement	"VR should be as convincingly realistic as possible to users, and the physical performance of a VR system is the key determinant of mental and physical immersion [33]."	Safety & comfort	Immersion tied to system p[illegible]	Physical Performance Evaluation
Zhang et al., 2020	Requirement	"This assessment method can facilitate user-centered design and can also yield information on the physical space required for users to fully explore the VR environment [33]."	Integration in workflow	Physical space as impleme[illegible]	Physical Performance Evaluation
Zhang et al., 2020	Barrier	"As another example, tasks involving actions that require users to reach out around their body to interact with nearby objects can be used to highlight unaddressed issues with distance compression, a frequent phenomenon within VR environments where objects are perceived by the user to be closer than their actual position [46]."	Safety & comfort	Perceptual distortion risk	Physical Performance Evaluation
Zhang et al., 2020	Barrier	"Following a given task, a user may achieve high task performance scores but still report heavy cognitive overload (ie, mental exhaustion) while using the system, for example, finding that performing the task in VR was significantly more difficult than performing the same task with real objects or tools."	Usability	Cognitive burden despite performance	Physical Performance Evaluation
Zhang et al., 2020	Requirement	"An optimized UI solution should provide the user with the best combination between immersion and usability, such that users feel immersed but unencumbered in accomplishing their tasks relative to outside a VR environment [14,34]."	Usability	Explicit UI design requirement	User Interface Evaluation
Zhang et al., 2020	Barrier	"If the integration between virtual and real-life tools is insufficient, it has been shown that this friction will cause users to prefer the No VR option, which may also be partially related to physical performance (see Physical Performance Evaluation section) [34]."	Integration in workflow	Direct rejection risk	User Interface Evaluation
Zhang et al., 2020	Barrier	"Given the lack of standardization across approaches, this warrants future research regarding the development of a comprehensive framework incorporating multiple methods of VR evaluation to provide, at a minimum, a strategic work plan for those looking to perform a baseline evaluation of any new VR application."	Evidence & credibility	Lack of standardized framework	Other Considerations
Zhang et al., 2020	Barrier	"The current literature base lacks practical examples of how to best use these approaches, which could be of great use to those employing them."	Evidence & credibility	Practical guidance gap	Other Considerations
Zhang et al., 2020	Requirement	"When developing VR interventions and applications, particularly in the context of health, the comfort of the end user is paramount."	Safety & comfort	Foundational safety requirement	Other Considerations
Zhang et al., 2020	Barrier	"VR still carries the risk of imposing symptoms similar to motion sickness during use as a result of visual distortions and asynchronies, among other effects [45]."	Safety & comfort	Cybersickness risk	Other Considerations

Zhang et al., 2020	Barrier	“These data are inherently subjective and the effects are, thus, not easily quantifiable enough to measure improvements.”	Evidence & credibility	Measurement limitation	Other Considerations
Zhang et al., 2020	Requirement	“Thus, the authors recommend that any VR assessment also explicitly consider the possible effect of motion sickness on its users by incorporating tools such as the Simulator Sickness Questionnaire (SSQ), originally developed to help measure motion sickness for pilots in flight simulators [45].”	Safety & comfort	Mandatory assessment tool	Other Considerations
Zhang et al., 2020	Requirement	“Like all emerging technologies in health care, there is a need to ensure the quality and safety of these novel tools [55].”	Safety & comfort	General safety obligation	Conclusions
Zhang et al., 2020	Requirement	“For VR, validated usability and assessment approaches are an important step before its deployment in real-world clinical settings.”	Evidence & credibility	Pre-deployment requirement	Conclusions
Zhang et al., 2020	Barrier	“This area of science is in its infancy and comprehensive knowledge translation would be critical to its growth.”	Evidence & credibility	Maturity and dissemination gap	Conclusions
