# Supplementary material for: The Multicriteria Decision Analysis for Extended Reality (MCDA-XR) Governance Framework for Health Care Adoption: Mixed Methods Development Study
Source: J Med Internet Res. 2026 Jul 31;28:e89801. doi: 10.2196/89801 (PMC13430000; doi:10.2196/89801)
Supplement: Multimedia Appendix 3 [file jmir-v28-e89801-s003.pdf]

Multimedia Appendix 3. Empirical Support for the Ten MCDA-XR Criteria: Thematic Determinant Synthesis

This appendix presents the thematic synthesis used to assess empirical support for the ten MCDA-XR criteria. Master themes were derived by clustering conceptually equivalent determinants across studies within each criterion. Frequency counts reflect the number of unique studies contributing to each theme, with repeated mentions within the same study counted once.

Themes were classified as Major when identified in three or more unique studies, as Critical when they represented safety, ethical, regulatory, or implementation-blocking concerns despite lower frequency, and as Minor when they were infrequent or not sufficiently distinct for retention in the final weighting structure.

Part A describes the LLM-assisted thematic synthesis protocol. Part B presents the resulting thematic evidence tables by criterion. The full determinant matrix underlying this synthesis is provided in Multimedia Appendix 2.

Part A. LLM-assisted thematic synthesis protocol

Overview

The thematic synthesis process was conducted in ChatGPT, using OpenAI GPT-family models within a project-specific configured environment. Methodological consistency was ensured through a predefined rule-based analytical protocol rather than reliance on a specific model release.

The model was used to support the structured aggregation of previously extracted and human-verified implementation determinants within each MCDA-XR criterion. It was explicitly constrained to perform rule-guided semantic clustering, assign higher-order theme labels, calculate study-level frequencies, and generate structured evidence matrices, without introducing new concepts or performing unsupported interpretation.

Clustering was performed by grouping determinants that referred to the same underlying concept, regardless of differences in wording. Multiple determinants from the same study contributing to a given concept were counted once for frequency calculation. As a result, the number of final themes does not correspond to the number of extracted determinants, but to the number of distinct conceptual clusters identified across studies. Determinants that were redundant, overlapping, or insufficiently specific to define an independent concept were absorbed into broader themes or not retained as standalone entries.

The procedure did not constitute autonomous qualitative analysis. All clustering outputs, theme definitions, frequencies, and classifications were subsequently reviewed manually by the research team to confirm conceptual consistency, source traceability, and alignment with the original determinant set.

The resulting evidence matrices provide a structured representation of recurrent and strategically relevant themes within each MCDA-XR criterion. The full analytical prompt and instruction set are provided below to support transparency and reproducibility.

Full prompt – Determinants Synthetizer

You are assisting with the structured thematic aggregation of implementation determinants to support the development of the MCDA-XR framework. Your task is to transform a structured list of previously extracted determinants into a consolidated evidence matrix for analytical review.

For each batch of determinants provided, perform a structured thematic aggregation using a rule-based clustering procedure. The objective is to identify recurrent concepts across studies, quantify their frequency, and classify their strategic relevance.

1. Input structure

You will receive data corresponding to one specific MCDA-XR criterion at a time.

Each input consists of a list or table with the following columns:

Study (Author, Year)
Type (Barrier, Facilitator, Requirement)
Raw Determinant (verbatim text)

All determinants belong to the same MCDA-XR criterion and must be analysed within that domain only.

2. Source fidelity and analytical boundaries

Use only the determinants provided in the input.

Do not introduce external knowledge.
Do not reinterpret determinants beyond their explicit meaning.
Do not merge concepts unless they are clearly semantically equivalent.

All outputs must remain traceable to the original determinants.

3. Semantic clustering

Group determinants that refer to the same underlying concept.

Clustering must follow these rules:

Only group determinants when they express the same concept, even if wording differs
Do not group determinants that are related but conceptually distinct
Preserve conceptual specificity, avoid overgeneralisation

When in doubt, keep determinants separated rather than merging

The goal is conceptual equivalence, not thematic similarity.

4. Theme labelling

For each cluster, assign a Master Theme Label.

The label must be:

Short and precise
Conceptually neutral
Descriptive of the underlying implementation factor
Written in professional, scientific language

Avoid vague or overly broad labels.

5. Frequency calculation

For each theme, calculate frequency (N) as:

The number of unique studies in which the theme appears

Rules:

If a study mentions the same concept multiple times, count it only once
Frequency is study-based, not determinant-based

6. Status classification

Classify each theme into one of the following categories:

Major Theme: appears in ≥ 3 unique studies

Critical Theme: appears in < 3 studies but represents a high-risk issue (e.g., safety, ethical, regulatory, or implementation-blocking factor)
Minor/Vague: non-recurrent or conceptually redundant

Minor/Vague themes should be excluded from the final table.

7. Description of concept

Provide a short, neutral description explaining the underlying concept represented by the theme.

The description must:

Reflect the meaning of the grouped determinants
Avoid interpretation beyond the data
Remain concise and precise

8. Source attribution

List all contributing studies for each theme:

Use format: Author, Year
Include each study only once per theme
Separate multiple sources with semicolons

9. Output format

Produce a single Markdown table with the following columns:

Master Theme Label
Status
Count (N)
Description of Concept
Sources (Author, Year)

Do not include raw determinants in the final output.

10. Analytical procedure

For each batch:

Review all determinants
Identify conceptually equivalent items
Cluster determinants into themes
Assign a Master Theme Label to each cluster
Calculate frequency at the study level
Classify themes by status
Remove Minor/Vague themes
Generate the final evidence matrix

11. Absolute prohibitions

Do not:

Introduce new concepts not present in the data
Merge distinct determinants into a broader category
Inflate frequency by counting the same study multiple times
Include themes without clear conceptual definition
Produce narrative summaries instead of the table

12. Additional notes

If ambiguity arises in clustering or classification:

Prefer conservative decisions
Preserve traceability to the original determinants
Avoid over-aggregation

Part B: The thematic evidence tables by criterion

Criterion 1. Relevance

Master Theme Label	Status	Count (N)	Description of Concept	Sources (Author, Year)
**Fit to patient profile, needs, and treatment goals**	Major	8	XR was considered more relevant when clearly matched to the right patient group, cognitive capacity, clinical profile, and therapeutic objective. Irrelevance was often framed as poor fit, uncertain benefit for certain populations, or lack of indication for a given case.	Abbas et al., 2024; Alrashidi et al., 2025; Chung et al., 2023; Felnhofer et al., 2025; Glegg & Levac, 2018; Kouijzer et al., 2023; Kouijzer et al., 2024; Terkildsen et al., 2024
**Real-world therapeutic applicability and transfer**	Major	7	Relevance depended on whether XR could support meaningful clinical work beyond the virtual session, particularly transfer of skills, behaviours, or insights to everyday functioning and real care processes.	Abbas et al., 2024; Elser et al., 2024; Glegg & Levac, 2018; Glegg et al., 2018; Kouijzer et al., 2024; Schreiter et al., 2025; Terkildsen et al., 2024
**Simulation and exposure of meaningful real-life situations**	Major	5	XR was valued when it enabled controlled recreation of situations, triggers, environments, or interpersonal contexts that are difficult to reproduce in standard care, especially for exposure, rehearsal, role play, or reflective work.	Abbas et al., 2024; Chung et al., 2022; Elser et al., 2024; Felnhofer et al., 2025; Kouijzer et al., 2024
**Alignment with a concrete clinical problem or unmet need**	Major	5	Adoption was more likely when XR addressed a recognised clinical gap, priority condition, or unresolved care challenge, and when it offered a plausible advantage over existing options.	Glegg & Levac, 2018; Lurtz et al., 2024; Sarkar et al., 2021; Schreiter et al., 2025; Terkildsen et al., 2024
**Availability of relevant, realistic, and sufficiently varied content**	Major	5	Relevance was strengthened when XR offered realistic environments, high graphical fidelity, diverse scenarios, frequent content updates, localisation, and specialised software adapted to the intended clinical use.	Abbas et al., 2024; Elser et al., 2024; Felnhofer et al., 2025; Mondal & Mondal, 2025; Terkildsen et al., 2024

Criterion 2. Evidence and Credibility

Master Theme Label	Status	Count (N)	Description of Concept	Sources (Author, Year)
**Demonstrated effectiveness and added clinical value**	Major	17	Adoption depends strongly on evidence that XR is effective, clinically useful, educationally beneficial, or at least comparable to existing care. Recurrent barriers were limited evidence, uncertainty about benefits, and skepticism about therapeutic or practical value.	Abbas et al., 2024; Alrashidi et al., 2025; Chung et al., 2022; Chung et al., 2023; Deason et al., 2025; Elser et al., 2024; Felnhofer et al., 2025; Glegg et al., 2018; Kouijzer et al., 2023; Lattré et al., 2025; Lurtz et al., 2024; Morgan et al., 2025; Sarkar et al., 2021; Schreiter et al., 2025; Shiner et al., 2024; Terkildsen et al., 2024; UCL & KCL, 2023
**Awareness of the evidence base among stakeholders**	Major	7	Beyond the existence of evidence, implementation is affected by whether clinicians or decision-makers know that evidence exists and understand its implications. Lack of awareness, being uninformed, or poor familiarity with available research was repeatedly described as a barrier.	Chung et al., 2022; Chung et al., 2023; Felnhofer et al., 2025; Glegg & Levac, 2018; Lattré et al., 2025; Schreiter et al., 2025; Shiner et al., 2024

**Rigorous validation and study design**	Major	8	Credibility requires methodologically robust evaluation, including mixed methods, longitudinal follow-up, external validation, representative users, control conditions, broader trials, and attention to validity, reliability, and generalisability. Weak design was framed as limiting confidence in findings and transferability.	Abbas et al., 2024; Kouijzer et al., 2023; Mondal & Mondal, 2025; Morgan et al., 2025; Pereira Guerreiro et al., 2025; Sarkar et al., 2021; Zhang et al., 2020; Deason et al., 2025
**Standardised guidance, definitions, and evaluation frameworks**	Major	7	Several studies highlighted the need for clearer standards to interpret and compare evidence, including common terminology, reporting conventions, evaluation frameworks, treatment guidance, evidence summaries, and decision criteria. Absence of such structure weakens interpretability and practical uptake.	Abbas et al., 2024; Deason et al., 2025; Glegg et al., 2018; Kouijzer et al., 2023; Lurtz et al., 2024; Shiner et al., 2024; Zhang et al., 2020
**Measurable and objective outcomes for ongoing appraisal**	Major	4	Credibility was also linked to whether XR effects can be measured clearly, objectively, and repeatedly over time. Studies emphasized observable results, predefined outcomes, retention measures, objective improvement, and feedback mechanisms for continuous refinement.	Abbas et al., 2024; Deason et al., 2025; Schreiter et al., 2025; Terkildsen et al., 2024

Criterion 3. Safety & comfort

Master Theme Label	Status	Count (N)	Description of Concept	Sources (Author, Year)
**Adverse Physical Effects and Cybersickness**	Major	10	Recurrent concern that VR may provoke nausea, dizziness, headaches, visual discomfort, motion sickness, vomiting, fatigue, or other bodily adverse effects during or after use. This was one of the most consistently reported safety barriers across studies.	Abbas, 2024; Chung, 2023; Felnhofer, 2025; Kouijzer, 2023; Kouijzer, 2024; Morgan, 2025; Sarkar, 2021; Schreiter, 2025; Terkildsen, 2024; Zhang, 2020
**Patient Suitability and Contraindication Screening**	Major	5	Safe use depends on assessing whether VR is appropriate for the individual patient, considering factors such as prior motion sickness, vision problems, pain severity, neck mobility, or broader clinical condition. Several studies framed this as a necessary precondition for implementation.	Elser, 2024; Felnhofer, 2025; Lurtz, 2024; Sarkar, 2021; Terkildsen, 2024
**Environmental and Procedural Safety Safeguards**	Major	5	Implementation is facilitated when the physical environment and supervision process reduce risk, for example through chaperoning, clinician presence, safe treatment-room setup, adequate movement space, or using VR as a safer setting for behavioral rehearsal.	Chung, 2023; Elser, 2024; Kouijzer, 2023; Kouijzer, 2024; Shiner, 2024
**Headset Ergonomics and Physical Comfort**	Major	4	The usability and acceptability of VR are influenced by device comfort, including headset weight, pressure on the face or nose, sense of enclosure, and the need for light and comfortable hardware adapted to clinical use.	Elser, 2024; Morgan, 2025; Schreiter, 2025; Zhang, 2020

**Psychological Distress and Altered Sense of Reality**	Major	3	Beyond physical side effects, some studies raised concerns that VR may heighten distress, unsettle reality testing, provoke anxiety, or intensify emotional arousal in vulnerable users. This reflects a distinct psychological safety dimension.	Chung, 2022; Chung, 2023; Kouijzer, 2024
**Safety Monitoring and Side-Effect Reporting**	Major	3	Several studies emphasized that adverse events and side effects should be actively documented, measured, and reported during evaluation and implementation, rather than assumed to be negligible.	Abbas, 2024; Terkildsen, 2024; Zhang, 2020
**Exposure Burden, Screen Time, and Tolerability Over Time**	Major	4	Safety and comfort were also linked to the duration and cumulative burden of use, including ability to tolerate longer sessions, concerns about excessive screen time, physical fatigue, and possible overuse.	Abbas, 2024; Glegg & Levac, 2018; Morgan, 2025; Schreiter, 2025
**Perceptual Coherence and Sensory Realism**	Major	4	Safe and comfortable VR use depends partly on perceptual congruence between the virtual environment and bodily experience. When realism, immersion, or spatial perception are poorly aligned, users may feel disoriented, unsettled, or physically uncomfortable.	Abbas, 2024; Chung, 2022; Kouijzer, 2024; Zhang, 2020

Criterion 4. Usability

Master Theme Label	Status	Count (N)	Description of Concept	Sources (Author, Year)
**Ease of use and low digital burden**	Major	11	Usability depends on whether the system is easy to learn, intuitive to operate, and manageable for users with different levels of technical confidence, language ability, age-related limitations, or physical capability.	Abbas et al., 2024; Chung et al., 2023; Elser et al., 2024; Glegg & Levac, 2018; Kouijzer et al., 2023; Kouijzer et al., 2024; Lattré et al., 2025; Lurtz et al., 2024; Morgan et al., 2025; Sarkar et al., 2021; Schreiter et al., 2025
**Technical robustness and software quality**	Major	8	Recurrent concern that usability is undermined by unstable, immature, low-quality, or outdated hardware and software, including glitches, failures, compatibility problems, and general technical difficulties.	Abbas et al., 2024; Chung et al., 2022; Felnhofer et al., 2025; Glegg & Levac, 2018; Lattré et al., 2025; Mondal & Mondal, 2025; Schreiter et al., 2025; UCL & KCL, 2023
**Customisation and fit to clinical tasks**	Major	7	Systems are more usable when content, interface options, and scenarios can be adapted to clinical goals, user preferences, and patient characteristics, rather than relying on rigid or generic designs.	Chung et al., 2022; Chung et al., 2023; Glegg et al., 2018; Kouijzer et al., 2024; Lattré et al., 2025; Morgan et al., 2025; Terkildsen et al., 2024
**Guided workflows, tutorials, and setup support**	Major	4	Stepwise navigation, plain-language instructions, previews, templates, and other scaffolding features improve usability by reducing confusion during setup and use.	Elser et al., 2024; Kouijzer et al., 2024; Lurtz et al., 2024; Schreiter et al., 2025
**Hardware ergonomics and interaction accessibility**	Major	4	Usability is affected by the physical demands of the hardware and input method, including headset weight, controller handling, cables, field of view, and the need for accessible hands-free interaction.	Chung et al., 2022; Deason et al., 2025; Elser et al., 2024; Schreiter et al., 2025

**Usability testing and validated assessment methods**	Major	4	Several studies emphasised the need to evaluate usability systematically through validated questionnaires, structured testing, trial use before adoption, and clearer interpretation of system-generated measures.	Abbas et al., 2024; Glegg et al., 2018; Schreiter et al., 2025; Zhang et al., 2020

Criterion 5. Integration in workflow

Master Theme Label	Status	Count (N)	Description of Concept	Sources (Author, Year)
**Infrastructure, space, and system compatibility**	Major	11	Workflow integration depends on having adequate physical space, portable or deployable hardware, compatible IT systems, connectivity, and interoperability with existing digital infrastructure or care platforms. Lack of space, fragmented systems, or poor compatibility can directly obstruct implementation.	Alrashidi, 2025; Deason, 2025; Glegg & Levac, 2018; Lurtz, 2024; Mondal & Mondal, 2025; Morgan, 2025; Pereira Guerreiro, 2025; Schreiter, 2025; Shiner, 2024; UCL & KCL, 2023; Zhang, 2020
**Time and workload fit within routine care**	Major	9	VR adoption is strongly shaped by whether it can be accommodated within busy clinical routines. Determinants include setup time, additional operational steps, clinician workload, scarce time during sessions, and whether the technology ultimately saves staff time or adds burden.	Alrashidi, 2025; Felnhofer, 2025; Kouijzer, 2023; Kouijzer, 2024; Lurtz, 2024; Sarkar, 2021; Schreiter, 2025; Shiner, 2024; Chung, 2022
**Need for structured protocols, guidance, and implementation planning**	Major	7	Integration is facilitated when services have clear protocols, manuals, decision guidance, and an agreed implementation plan for how VR should be introduced, discussed by teams, and embedded within treatment pathways or curricula. Lack of such structure creates inconsistency and uncertainty.	Abbas, 2024; Chung, 2023; Kouijzer, 2023; Kouijzer, 2024; Sarkar, 2021; Schreiter, 2025; Terkildsen, 2024
**Compatibility with existing clinical routines and care models**	Major	6	Successful workflow integration depends on whether VR fits current service organization, such as group versus individual formats, ward routines, existing treatment pathways, and broader care structures. When VR requires substantial changes to established practice, feasibility is reduced.	Abbas, 2024; Chung, 2022; Chung, 2023; Kouijzer, 2023; Lurtz, 2024; Sarkar, 2021; Schreiter, 2025
**Efficiency, autonomy, and distributed delivery as workflow enablers**	Major	8	VR is perceived as easier to integrate when it supports more efficient care processes, enables patient self-practice or home use, reduces dependence on clinician presence, facilitates group delivery, or streamlines documentation and task allocation.	Chung, 2022; Deason, 2025; Glegg & Levac, 2018; Lurtz, 2024; Pereira Guerreiro, 2025; Sarkar, 2021; Schreiter, 2025; Shiner, 2024

Criterion 6. Resources & cost

Master Theme Label	Status	Count (N)	Description of Concept	Sources (Author, Year)

**Affordability and funding constraints**	Major	16	VR adoption was repeatedly shaped by the direct and indirect financial burden of implementation. This included high acquisition and running costs, limited budgets, lack of funds, uncertain return on investment, and concerns about whether organisations could absorb the economic burden of hardware, software, maintenance, and scale-up.	Abbas, 2024; Alrashidi, 2025; Chung, 2022; Chung, 2023; Deason, 2025; Felnhofer, 2025; Kouijzer, 2023; Lattré, 2025; Lurtz, 2024; Mondal and Mondal, 2025; Morgan, 2025; Sarkar, 2021; Schreiter, 2025; Shiner, 2024; Terkildsen, 2024; UCL and KCL, 2023
**Infrastructure, equipment, and deployment capacity**	Major	10	Implementation depended on the material and technical infrastructure needed to deploy VR in practice. This included access to hardware, suitable rooms or treatment space, reliable internet and power supply, transportability, and compatibility with existing digital systems or local service conditions.	Abbas, 2024; Alrashidi, 2025; Chung, 2023; Deason, 2025; Felnhofer, 2025; Mondal and Mondal, 2025; Morgan, 2025; Sarkar, 2021; Schreiter, 2025; UCL and KCL, 2023
**Staffing, technical support, maintenance, and protected time**	Major	9	Adoption required human and operational resources beyond device purchase. Recurring determinants included the need for support staff, setup assistance, maintenance capacity, workforce support, clinician coaching, and protected time for administration, training, and use.	Abbas, 2024; Alrashidi, 2025; Kouijzer, 2023; Kouijzer, 2024; Lurtz, 2024; Morgan, 2025; Sarkar, 2021; Shiner, 2024; UCL and KCL, 2023
**Reimbursement and sustainable business model viability**	Major	8	Beyond simple cost, several studies stressed the need for a viable financial model to sustain implementation. This included reimbursement pathways, insurance coverage, billability, tariff structures, cost-effectiveness, and broader concerns about whether VR could be maintained within real healthcare financing systems.	Abbas, 2024; Chung, 2022; Chung, 2023; Lurtz, 2024; Sarkar, 2021; Schreiter, 2025; Shiner, 2024; Terkildsen, 2024
**Resource intensity of tailoring and local content development**	Major	4	Some studies emphasised that adapting VR to local needs was itself resource-demanding. This included the cost and effort of generating tailored content, producing local recordings or applications, and accessing software development or design expertise for customisation.	Deason, 2025; Schreiter, 2025; Shiner, 2024; Terkildsen, 2024

Criterion 7. Training requirement

Master Theme Label	Status	Count (N)	Description of Concept	Sources (Author, Year)
**Lack of knowledge and awareness of VR**	Major	12	Limited or absent understanding of VR, its clinical applications, and potential benefits among professionals. Includes unfamiliarity, lack of exposure, and misconceptions about VR as only entertainment technology.	Chung 2023; Felnhofer 2025; Kouijzer 2023; Lattré 2025; Sarkar 2021; Schreiter 2025; Shiner 2024; UCL & KCL 2023; Mondal 2025; Elser 2024; Glegg & Levac 2018; Kouijzer 2024
**Need for structured training and education programs**	Major	14	Requirement for formal, structured training initiatives, including courses, workshops, university curricula, and continuous education to support VR adoption in clinical practice.	Chung 2022; Chung 2023; Felnhofer 2025; Elser 2024; Kouijzer 2023; Kouijzer 2024; Lurtz 2024; Shiner 2024; UCL & KCL 2023; Pereira Guerreiro 2025; Sarkar 2021; Deason 2025; Lattré 2025; Mondal 2025

**Development of practical skills and clinical competencies**	Major	11	Need for hands-on skills to operate VR systems, apply them clinically, manage patients, and optimize outcomes. Includes technical, procedural, and therapeutic competencies.	Chung 2022; Chung 2023; Elser 2024; Felnhofer 2025; Glegg & Levac 2018; Kouijzer 2024; Mondal 2025; Shiner 2024; Sarkar 2021; UCL & KCL 2023; Schreiter 2025
**Experiential learning and supervised practice**	Major	8	Importance of learning through direct experience, guided onboarding, mentorship, supervised sessions, and iterative practice to build confidence and competence.	Felnhofer 2025; Kouijzer 2024; Shiner 2024; UCL & KCL 2023; Schreiter 2025; Pereira Guerreiro 2025; Sarkar 2021; Lurtz 2024
**Time required for training and learning**	Major	5	Training is perceived as time-intensive, including time to learn, practice, educate patients, and integrate knowledge into routine care. Time constraints act as a barrier.	Alrashidi 2025; Kouijzer 2023; Kouijzer 2024; Lurtz 2024; Sarkar 2021
**Low confidence and self-efficacy in VR use**	Major	6	Lack of confidence among clinicians to use VR effectively, often linked to insufficient training, limited experience, or perceived technical complexity.	Kouijzer 2023; Glegg & Levac 2018; Schreiter 2025; Felnhofer 2025; Elser 2024; Shiner 2024
**Need for patient onboarding and education**	Major	6	Requirement to educate and guide patients in using VR, including orientation, expectation setting, and ongoing support during use.	Chung 2022; Kouijzer 2024; Lurtz 2024; Sarkar 2021; UCL & KCL 2023; Elser 2024
**Role of prior experience and exposure to VR**	Major	5	Previous exposure to VR facilitates adoption, while lack of prior experience acts as a barrier. Familiarity improves acceptance and capability.	Schreiter 2025; Shiner 2024; Felnhofer 2025; Glegg & Levac 2018; UCL & KCL 2023
**Availability of training resources and materials**	Minor	2	Use of supporting materials such as brochures, videos, and psychoeducational content to facilitate learning and adoption.	Kouijzer 2024; Shiner 2024

Criterion 8. Patient acceptability

Master Theme Label	Status	Count (N)	Description of Concept	Sources (Author, Year)
**Motivation, enjoyment, and willingness to engage with VR**	Major	12	VR was often perceived as appealing, enjoyable, motivating, or satisfying for patients. This included curiosity, playfulness, gaming elements, positive first impressions, recommendation to others, and greater willingness to try or continue use when the experience felt engaging.	Alrashidi, 2025; Chung, 2022; Elser, 2024; Felnhofer, 2025; Glegg & Levac, 2018; Kouijzer, 2024; Lurtz, 2024; Sarkar, 2021; Schreiter, 2025; Chung, 2023; Deason, 2025; Abbas, 2024
**Age, digital literacy, and prior familiarity shape acceptability**	Major	8	Patient openness to VR was frequently linked to age, technical confidence, prior exposure to technology, and broader digital capability. Younger users were sometimes perceived as more receptive, whereas older adults or less technically skilled users were more often seen as hesitant, although this was not consistent across all studies.	Chung, 2022; Chung, 2023; Glegg & Levac, 2018; Schreiter, 2025; Shiner, 2024; Mondal & Mondal, 2025; Lurtz, 2024; Sarkar, 2021

**Preservation of human connection and therapeutic relationship**	Major	6	Acceptability was reduced when VR was perceived as isolating, depersonalizing, or less authentic than interaction with a real clinician. Concerns included headset-related social disconnection, weaker rapport, and the sense that virtual interaction may not adequately replace human contact.	Felnhofer, 2025; Kouijzer, 2023; Kouijzer, 2024; Morgan, 2025; Shiner, 2024; Glegg & Levac, 2018
**Cultural, linguistic, and contextual fit of content**	Major	4	Patient acceptance depended in part on whether VR content was understandable, culturally appropriate, and relevant to diverse populations. Language barriers, limited cultural resonance, and insufficient tailoring could reduce usability and willingness to engage, whereas adapted content could facilitate uptake.	Sarkar, 2021; Mondal & Mondal, 2025; Shiner, 2024; Lurtz, 2024
**Communication, explanation, and expectation management**	Major	4	Clear explanation, education, and preparatory communication appeared to improve willingness to try VR and reduce aversion or mistrust. Acceptability was stronger when patients understood the purpose of VR, had realistic expectations, and were actively informed about its use.	Lurtz, 2024; Sarkar, 2021; Schreiter, 2025; Elser, 2024
**Perceived realism and emotional resonance of the virtual experience**	Major	3	Acceptance was influenced by whether patients experienced VR as believable, immersive, and emotionally meaningful. Reduced realism, difficulty responding to scenarios as if they were real, or limited resonance with the content could weaken engagement and perceived usefulness.	Kouijzer, 2024; Lurtz, 2024; Sarkar, 2021

Criterion 9. Institutional support

Master Theme Label	Status	Count (N)	Description of Concept	Sources (Author, Year)
**Leadership and management support**	Major	14	Organisational adoption is facilitated when senior leadership, managers, or decision-makers actively endorse XR, provide direction, and signal that implementation is a legitimate institutional priority. Lack of such support functions as a barrier.	Alrashidi, 2025; Kouijzer, 2023; Kouijzer, 2024; Lurtz, 2024; Sarkar, 2021; Schreiter, 2025; Shiner, 2024; UCL & KCL, 2023; Felnhofer, 2025; Terkildsen, 2024; Chung, 2023; Chung, 2022; Glegg & Levac, 2018; Elser, 2024
**Organisational culture, openness, and staff buy-in**	Major	13	Adoption depends on a local culture that is receptive to innovation, with clinicians and staff showing interest, openness, and willingness to engage. Resistance to change, fear of new approaches, lack of motivation, and negative staff attitudes undermine implementation.	Chung, 2022; Chung, 2023; Alrashidi, 2025; Elser, 2024; Felnhofer, 2025; Glegg & Levac, 2018; Lurtz, 2024; Mondal & Mondal, 2025; Sarkar, 2021; Schreiter, 2025; Shiner, 2024; Terkildsen, 2024; UCL & KCL, 2023
**Champions, ambassadors, and opinion leaders**	Major	8	Local advocates such as champions, ambassadors, mentors, or respected clinicians help normalise XR, influence peers, and sustain change efforts. Their absence can weaken diffusion within teams.	Chung, 2022; Lurtz, 2024; Sarkar, 2021; Shiner, 2024; Glegg & Levac, 2018; Schreiter, 2025; Kouijzer, 2024; UCL & KCL, 2023

**Collaborative governance and stakeholder planning**	Major	8	Successful implementation requires structured collaboration across stakeholders, including multidisciplinary planning, participatory decision-making, and forums where clinical, technical, and organisational perspectives can be aligned.	Chung, 2022; Deason, 2025; Glegg et al., 2018; Terkildsen, 2024; Schreiter, 2025; UCL & KCL, 2023; Kouijzer, 2023; Abbas, 2024
**Policy, strategy, and implementation governance**	Major	9	XR uptake is strengthened by formal policies, strategies, frameworks, committees, and systematic implementation processes. Absence of clear directives, national prioritisation, or governance structures creates institutional uncertainty and weakens scalability.	Chung, 2023; Deason, 2025; Felnhofer, 2025; Kouijzer, 2023; Mondal & Mondal, 2025; Morgan, 2025; Shiner, 2024; Terkildsen, 2024; Abbas, 2024
**Organisational readiness and support capacity**	Major	9	Adoption depends on whether the organisation is practically prepared, including digital readiness, infrastructure readiness, workforce capacity, IT support, and workplace support. Weak readiness or insufficient support capacity hinders implementation.	Abbas, 2024; Deason, 2025; Felnhofer, 2025; Kouijzer, 2023; Shiner, 2024; Terkildsen, 2024; UCL & KCL, 2023; Elser, 2024; Chung, 2023
**Reimbursement, insurance, and financial-system support**	Major	3	Broader institutional adoption is constrained when XR lacks reimbursement pathways, insurance approval, or public-sector financial recognition. System-level funding support is therefore a key implementation condition.	Felnhofer, 2025; Mondal & Mondal, 2025; Morgan, 2025
**Diffusion, scale-up, and cross-organisational spread**	Major	5	Some studies frame institutional support not only as local approval but as the ability to spread successful XR solutions across departments, sites, or the wider system through coordinated scaling and organisational learning.	Terkildsen, 2024; Deason, 2025; UCL & KCL, 2023; Schreiter, 2025; Chung, 2023
**Organisational incentives and strategic positioning**	Major	5	Institutions may support XR when it is seen as offering strategic value, such as service capacity gains, competitive differentiation, recruitment advantages, or role expansion for staff.	Chung, 2023; Lurtz, 2024; Schreiter, 2025; Terkildsen, 2024; Abbas, 2024

Criterion 10. Legal & ethical alignment

Master Theme Label	Status	Count (N)	Description of Concept	Sources (Author, Year)
**Data privacy, security and confidentiality**	Major	7	Concerns related to protection of patient data, confidentiality, cybersecurity, sensitive information handling, and secure management of digital or XR-generated data. Includes both barriers arising from privacy risk and facilitators when systems avoid storing identifiable data.	Chung, 2023; Glegg & Levac, 2018; Kouijzer, 2023; Morgan, 2025; Sarkar, 2021; Schreiter, 2025; Lurtz, 2024
**Regulatory uncertainty, standards and compliance burden**	Major	5	Lack of clear regulation, unclear standards, certification ambiguity, and compliance requirements that complicate implementation, increase development burden, or slow adoption. Includes the need for external clinical regulation and clearer accepted standards.	Chung, 2023; Lurtz, 2024; Morgan, 2025; Glegg & Levac, 2018; Schreiter, 2025

**Ethical and professional boundary concerns**	Major	4	Ethical issues linked to appropriate clinical conduct, professional boundaries, consent, patient-provider relationship, and prevention of harm during VR use. Includes concern about physical touch and maintaining safe therapeutic boundaries.	Chung, 2022; Chung, 2023; Glegg & Levac, 2018; Morgan, 2025
**Need for formal protocols, governance and institutional policy frameworks**	Major	3	Need for explicit protocols, governance structures, or institutional policy guidance to ensure safe, ethical, and operationally consistent implementation of VR in healthcare settings.	Chung, 2022; Lurtz, 2024; Morgan, 2025
**Reimbursement, tariff and policy fit**	Major	3	Misalignment between VR implementation and existing reimbursement or tariff systems, including uncertainty about service categorisation and the need for policy advocacy to enable sustainable adoption.	Lurtz, 2024; Morgan, 2025; Chung, 2023
**Consumer-healthcare boundary ambiguity**	Critical	1	Unclear distinction between healthcare-grade XR applications and consumer-facing tools, creating legal, regulatory, and strategic uncertainty for implementation and commercialization.	Morgan, 2025
**Combined AI and XR regulatory complexity**	Critical	1	Additional legal and ethical complexity introduced when XR is combined with AI, creating compounded compliance and governance challenges.	Morgan, 2025
