# Supplementary material for: The Multicriteria Decision Analysis for Extended Reality (MCDA-XR) Governance Framework for Health Care Adoption: Mixed Methods Development Study
Source: J Med Internet Res. 2026 Jul 31;28:e89801. doi: 10.2196/89801 (PMC13430000; doi:10.2196/89801)
Supplement: Multimedia Appendix 4 [file jmir-v28-e89801-s004.pdf]

Multimedia Appendix 4. Theoretical–Empirical Framework Alignment Underlying the Ten MCDA-XR Criteria

This table outlines the conceptual foundations of the MCDA-XR tool. Each of the ten criteria is linked to the structural domains of NASSS, the behavioural mechanisms of COM-B, the perceptual constructs of TAM, and the XR-specific descriptors of RATE-XR. The table clarifies how these frameworks jointly inform the conceptual meaning of each criterion and shows how they translate into practical evaluative questions for healthcare settings.

MCDA-XR Criterion	Relevant NASSS Domains	COM-B Mechanisms	TAM Constructs	RATE-XR Descriptors	Key Empirical Determinants (Synthesized from Extraction in SM2)	Example Indicators
**1. Relevance**	**The Condition**	Psychological capability	Perceived usefulness	Indication clarity; symptom relevance	Indication clarity, therapeutic value, alignment with patient goals, comparative advantage, ecological validity, breadth of use cases, cultural fit, therapist compatibility, expected patient demand, suitability across diverse groups, adjunctive role in care.	Clinical goal alignment; suitability for cognitive or sensory load
**2. Evidence & credibility**	**The Condition; The Wider System**	Reflective motivation	Perceived usefulness	Evidence transparency; methodological clarity	Weak evidence base, need for standardised methods, lack of guidelines, limited interpretability, poor generalisability, need for representative testing, ethical requirement for validation, transparency of vendor claims, objective measurable outcomes, safety concerns for specific populations.	Published evidence; safety documentation; clinical legitimacy
**3. Safety & comfort**	**The Technology**	Physical capability; automatic motivation	Perceived ease of use (comfort burden)	Cybersickness; sensory load; ergonomics	Cybersickness and physical symptoms, ergonomic discomfort, emotional distress, clinical contraindications, need for screening and supervision, perceived safety as enabler, symptom burden constraints.	Cybersickness incidence; tracking stability; tolerability thresholds
**4. Usability**	**The Technology**	Physical capability	Perceived ease of use	Interaction fidelity; setup burden; reliability	Ease of use, technical stability, software maturity, accessibility, realism, clarity of instructions, cognitive load, deployment constraints, need for customisation, influence of digital literacy.	SUS scores; task completion rates; setup time
**5. Integration in workflow**	**The Organisation**	Physical and social opportunity	Perceived usefulness (fit)	Workflow compatibility; environmental constraints	Space requirements, time burden, fit with routines, need for standardised protocols, portability constraints, IT compatibility, scheduling difficulties, competing priorities, connectivity barriers, potential for autonomous or home use.	Staff time burden; physical room layout; infection control procedures
**6. Resources & cost**	**The Value Proposition**	Physical opportunity	Perceived usefulness (cost-benefit)	Hardware requirements; maintenance needs	High costs, lack of reimbursement, infrastructure limitations, insufficient equipment and space, maintenance burden, need for technical support, staff time constraints, sustainability concerns, equity challenges, vendor lock-in risks, training as a resource burden.	Device cost; IT support needs; software licences and replacement cycles
**7. Training requirement**	**The Adopters**	Psychological capability	Perceived ease of use	Training descriptors; supervision needs	Limited VR skills, lack of time for learning, need for structured multi-stage training, influence of prior experience, importance of hands-on exposure, attitudinal barriers, need for clinical guidelines, need for educational materials, demands imposed by rapid technological change.	Staff familiarity; learning curve; required level of assistance
**8. Patient acceptability**	**The Adopters**	Reflective and automatic motivation	Perceived usefulness; attitude	User experience; emotional responses	Engagement and motivation, perceived benefit, influence of age and tech comfort, trust and realism, effects on therapeutic relationship, cultural and linguistic fit, need for personalisation and choice, risk of refusal, suitability for specific clinical populations, need for pre-use education.	Trust; comfort; engagement; willingness to repeat
**9. Institutional support**	**The Organisation**	Social opportunity	Perceived usefulness (institutional)	Readiness descriptors; organisational support	Leadership backing, organisational readiness, governance and procurement structures, IT and implementation support, external partnerships, financial viability, presence of champions, peer networks, competing policy priorities, data governance and monitoring infrastructures.	Leadership endorsement; IT support availability; innovation culture

**10. Legal & ethical alignment**	**The Wider System**	Social opportunity (structural constraints)	*External constraint (non-TAM)*	GDPR compliance; safety reporting; device classification	Privacy and data security concerns, regulatory uncertainty, ethical issues in consent and therapeutic boundaries, compliance burden, need for institutional advocacy for reimbursement, clarity of data-protection communication.	Regulatory conformity; privacy safeguards; ethical approval status
