# Supplementary material for: The Multicriteria Decision Analysis for Extended Reality (MCDA-XR) Governance Framework for Health Care Adoption: Mixed Methods Development Study
Source: J Med Internet Res. 2026 Jul 31;28:e89801. doi: 10.2196/89801 (PMC13430000; doi:10.2196/89801)
Supplement: Multimedia Appendix 5 [file jmir-v28-e89801-s005.pdf]

Multimedia Appendix 5. Participant Demographics and Professional Roles (Phase 2 Construction)

This table details the composition of the 33 stakeholders who participated in the construction and refinement sessions. Participants were drawn from four distinct groups representing hospital care, primary care, and academic research.

Group & Setting	ID	Professional Role / Category	Gender	Age Range	Prior Experience with XR
**Group 1: Hospital Specialists (Visiting)**<br><br>**(Professionals from various hospitals in the Barcelona area; session held at BSA Intermediate Care Center)**					
	P1	Psychology	Female	60–69	None
	P2	Medicine (Psychiatry)	Female	50–59	None
	P3	Medicine (Geriatrics)	Female	60–69	None
	P4	Medicine (Dementia Unit)	Female	40–49	Recreational only
	P5	Nursing (Dementia Unit)	Male	40–49	Recreational only
	P6	Medicine (Geriatrics)	Female	30–39	Recreational only
	P7	Medicine (Geriatrics)	Female	50–59	Recreational only
	P8	Nursing (Case Manager)	Female	60–69	None
	P9	Other (Healthcare Staff)	Male	50–59	Recreational only
	P10	Nursing (Dementia Unit)	Female	60–69	None
**Group 2: Primary Care Team**<br><br>**(CAP Lloret - Consorci Sanitari del Maresme i la Selva; RedocVR Project)**					
	P11	Physiotherapy	Female	30–39	Recreational only
	P12	Service Direction / Coordination	Female	40–49	None
	P13	Physiotherapy	Female	30–39	None
	P14	Administration / Admissions	Female	30–39	Yes (Clinical Pilot)
	P15	Nursing	Male	50–59	Recreational only
	P16	Service Direction / Coordination	Female	50–59	None
**Group 3: Academic Researchers**<br><br>**(Universitat Autònoma de Barcelona - UAB; Master's in Psychogerontology)**					
	P17	Postgraduate Psychologist (Student)	Female	18–29	None
	P18	Postgraduate Psychologist (Student)	Female	18–29	Recreational only
	P19	Postgraduate Psychologist (Student)	Female	18–29	None
	P20	Postgraduate Psychologist (Student)	Male	18–29	None
	P21	Postgraduate Psychologist (Student)	Female	18–29	Recreational only
	P22	Postgraduate Psychologist (Student)	Female	18–29	None
	P23	Postgraduate Psychologist (Student)	Female	18–29	Recreational only
	P24	Postgraduate Psychologist (Student)	Female	18–29	None
	P25	Postgraduate Psychologist (Student)	Female	18–29	Recreational only
	P26	Postgraduate Psychologist (Student)	Female	18–29	Recreational only
	P27	Postgraduate Psychologist (Student)	Male	18–29	Yes (Clinical Pilot)
	P28	Postgraduate Psychologist (Student)	Female	18–29	None
**Group 4: Rehabilitation Team**<br><br>**(Badalona Serveis Assistencials - BSA; Intermediate Care)**					
	P29	Neuropsychology	Female	40–49	Yes (Clinical Pilot)
	P30	Occupational Therapy	Female	40–49	None
	P31	Physiotherapy	Female	30–39	None
	P32	Occupational Therapy	Female	30–39	Yes (Clinical Pilot)
	P33	Physiotherapy	Female	40–49	Recreational only
**TOTAL SUMMARY**	**N=33**		**F: 28 (84.8%)**<br><br>**M: 5 (15.2%)**	**Median Group:**<br><br>**30–39 years**	**None: 16 (48.5%)**<br><br>**Recreational: 13 (39.4%)**<br><br>**Clinical Pilot: 4 (12.1%)**
